# Amino- and polyaminophthalazin-1(2*H*)-ones: synthesis, coordination properties, and biological activity

**DOI:** 10.3762/bjoc.17.50

**Published:** 2021-02-25

**Authors:** Zbigniew Malinowski, Emilia Fornal, Agata Sumara, Renata Kontek, Karol Bukowski, Beata Pasternak, Dariusz Sroczyński, Joachim Kusz, Magdalena Małecka, Monika Nowak

**Affiliations:** 1 Department of Organic Chemistry, Faculty of Chemistry, University of Lodz, Tamka 12, 91-403 Łódź, Poland; 2 Department of Pathophysiology, Medical University of Lublin, Jaczewskiego 8b, 20-090 Lublin, Poland; 3 Department of Molecular Biotechnology and Genetics, Faculty of Biology and Environmental Protection, University of Lodz, Banacha 12/16, 90-237 Łódź, Poland; 4 Laboratory of Molecular Spectroscopy, Faculty of Chemistry, University of Lodz, Tamka 12, 91-403 Łódź, Poland; 5 Department of Inorganic and Analytical Chemistry, Faculty of Chemistry, University of Lodz, Tamka 12, 91-403 Łódź, Poland; 6 Institute of Physics, University of Silesia, 75 Pułku Piechoty 1, 41-500 Chorzów, Poland; 7 Department of Physical Chemistry, Theoretical and Structural Chemistry Group, Faculty of Chemistry, University of Lodz, Pomorska 163/165, 90-236 Łódź, Poland

**Keywords:** amination, complexes, cytotoxicity, Pd cross-coupling, phthalazinone

## Abstract

Amino- and polyaminophthalazinones were synthesized by the palladium‐catalyzed amination (alkyl- and arylamines, polyamines) of 4-bromophthalazinones in good yields. The coordinating properties of selected aminophthalazinones towards Cu(II) ions were investigated and the participation of the nitrogen atoms in the complexation of the metal ion was shown. A biological screening of the potential cytotoxicity of selected synthesized compounds on HT-29 and PC-3 cell lines, as well as on the L-929 cell line, proved that some amino derivatives of phthalazinone show interesting anticancer activities. The detailed synthesis, spectroscopic data, and biological assays are reported.

## Introduction

Phthalazine and its analogs are an interesting group of pharmacologically active heterocycles [1–2], many of which possess, e.g., antimicrobial [3–4], antifungal [5], antidepressant [6–7], and antihistaminic [8–10] properties. Amino- and amidophthalazine derivatives have been examined, e.g., as inhibitors of PGE_2_ production [11] that plays an important role in the growth of various cancerous tissues (colon, lung, and breast cancer) and as antagonists of the human A_3_ adenosine receptor [12].

**Figure 1:**
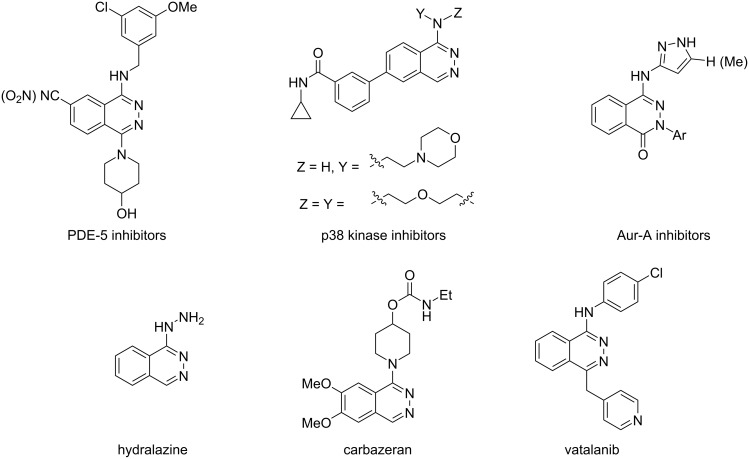
Structure of biologically active phthalazine derivatives.

Aminophthalazines have been also evaluated for their inhibitory activity toward phosphodiesterases such as PDE-5 (Figure 1) [13–14] and PDE-10 [15] for a potential use in the treatment of chronic pain and neurodegenerative or psychiatric disorders. Some of these derivatives are known to possess anti-inflammatory (p38 MAP kinase inhibitors, Figure 1) [16], cardiotonic [17], and anticancer (Aurora-A kinase inhibitors) properties [18–20].

The aminophthalazine and also hydrazinylphthalazine moiety can be also found in the core of many commercial drugs such as hydralazine [21–23], budralazine [24], and carbazeran [25] that are used for the treatment of heart failure as well as in the structure of the effective anticancer drug vatalanib [26–28] (Figure 1). On the other hand, aminophthalazinones can be prospective candidates as *N*- and *O*-donor ligands to form complexes with biological significant metal ions, such as copper or zinc [29].

**Scheme 1:**
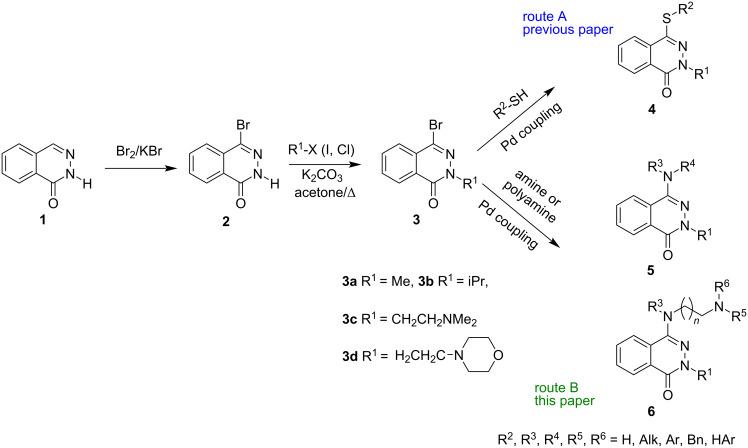
Synthetic route to aminophthalazinones **5** and **6**.

Recently, we have demonstrated a strategy for the synthesis of phthalazinone and phthalazine derivatives of type **4** containing an alkylsulfanyl functional group at the 4 position, that is based on the Pd-catalyzed coupling reaction between mercaptanes and 4-bromolactams (Scheme 1, route A) [30]. In continuing our efforts on the functionalization of phthalazinones and quinazolinones [30–31] and taking into consideration the biological importance of aminophthalazine derivatives, we decided to apply the methodology based on the palladium-catalyzed C–N-bond formation (Buchwald–Hartwig-type reaction) as a convenient and effective approach for the synthesis of the new phthalazinone derivatives **5** and **6** (Scheme 1, route B).

In the literature, the number of reported synthetic methods for 4-aminophthalazin-1(2*H*)-ones is limited to a few examples and they usually involve two main approaches: 1) the application of multicomponent reactions starting from, e.g., the available *o*-bromobenzoate via palladium-catalyzed isocyanide insertion [32–33] (a method that is limited to tertiary-substituted isocyanides) or 2) the palladium or copper-catalyzed coupling of bromolactams with amines (a method that requires the usually lengthy synthesis of the bromoprecursors) [19,34].

Therefore, the need to develop new and improve the existing methods for the synthesis of aminophthalazinones is important from the point of view of their properties and pharmaceutical industry interest.

In the present work, we report results of our research on the synthesis, application as ligands in complexes, and cytotoxic activity of amino- and polyaminophthalazinone derivatives.

## Results and Discussion

### Chemistry

#### Synthesis of aminophthalazinones

The synthetic route toward the aminophthalazinones **5** and **6** is shown in Scheme 1 (route B). A current literature review [19,34] and our experience [35] proved, that the direct Pd-coupling of bromophthalazinones of type **2** and also bromoquinazolinones, having an N–H moiety (acidic hydrogen atom) with amines does not yield satisfying results without an *N*-protection of the amide function.

Therefore, in the initial stage of our studies, the *N*-substituted phthalazin-1(2*H*)-ones **3a**–**d** were prepared in two steps starting from phthalazin-1(2*H*)-one (**1**). The 4-bromo-derivative **2** was synthesized directly from lactam **1**, which underwent the selective bromination at the 4-position using the combination of Br_2_ and KBr (KBr_3_) in acetate buffer, following the method previously reported by us [30]. It is known that the alkylation of phthalazinones depends on the reaction conditions and can proceed in two ways involving either the nitrogen or the oxygen atom (lactam–lactim tautomerism). It has been proven that potassium salts of phthalazinones or the bromo derivatives are selectively alkylated on the nitrogen atom [30,36]. For our purposes, the simple alkyl halides (MeI, iPrI), and 2-chloro-*N*,*N*-dimethylethylamine hydrochloride and 4-(2-chloroethyl)morpholine hydrochloride, were chosen as the alkylating agents. Thus, the desired *N*-methyl and *N*-isopropyl lactams **3a**,**b** (**3a** R^1^ = Me, 85%; **3b** R^1^ = iPr, 84%) were obtained by the direct alkylation of bromophthalazinone **2** with methyl or isopropyl iodide in the presence of K_2_CO_3_ in dry acetone as the solvent (conventional heating). In the similar way also the 2-aminoethyl lactams **3c** (R^1^ = CH_2_CH_2_NMe_2_) and **3d** (R^1^ = CH_2_CH_2_(morpholin-4-yl) were synthesized with 53% and 61% yields, respectively. The methodology turned out to be the most efficient out of the tested ones, especially for the products **3c** and **3d**. The formation of the *N*-alkylated products **3** was confirmed on the basis of their spectral analysis (see Supporting Information File 1).

In the next step, the bromolactams **3** were subjected to a thermal palladium cross-coupling reaction with various amines and polyamines. To optimize the reaction conditions, we investigated the reaction of 4-bromo-2-methylphthalazin-1(2*H*)-one (**3a**) with morpholine as the model substrates. Previously, we have observed that the coupling system involving Xantphos/Pd(OAc)_2_ (used in the ratio of 15 mol %/15 mol % or 30 mol %/30 mol %) and *t-*BuOK or DIPEA in 1,4-dioxane as the solvent was effective for the C–N or C–S bond formation [30–31,35].

Unfortunately, it turned out, that the application of Xantphos/Pd(OAc)_2_/*t*-BuOK and our standard procedure [30,35], in which the amine is added after the lactam **3a,** for the initial experiments ended with failure. In most cases, regardless of the used catalytic system (Pd source: Pd(OAc)_2_, Pd_2_(dba)_3_, ligand: DPEPhos, DavePhos, BINAP), solvent (1,4-dioxane, toluene) and base (*t*-BuOK, DIPEA, Cs_2_CO_3_), the ^1^H NMR spectra of the post-reaction mixtures indicated the presence of unchanged substrate **3a**. Also, the variation of the reactant quantities (ligand/Pd source = 15:15, 23:15, 23:7.5, 20:20 mol %) and the reaction time did not have a positive effect on the course of the reaction and in several cases resulted in the formation of 2-methylphthalazin-1(2*H*)-one, i.e., the debromination product of bromophthalazinone **3a**.

The commonly adopted view on the mechanism of the Pd-mediated C–N-bond formation (Buchwald–Hartwig-type coupling) [37–40] assumes that the coordination of the amine takes place after the oxidative addition of the organic halide to the palladium–ligand complex. Based on the analysis of our results we have concluded that in the case of systems **3**, the order of reagent addition could have a significant impact on a proper course of the cross-coupling reaction. To confirm this idea, we carried out an experiment in which to the in situ-generated (BINAP)Pd complex (Pd_2_(dba)_3_/*rac*-BINAP or (*R*)-BINAP; 15:15 mol %) morpholine was added prior to the addition of lactam **3a**. As a result, we obtained the target 4-(morpholin-4-yl) derivative **5a** in 77% yield. Moreover, it turned out that this amination reaction also proceeded with a reduced amount of Pd_2_(dba)_3_ from 15 mol % to 2 mol % without loss of the product yield.

**Figure 2:**
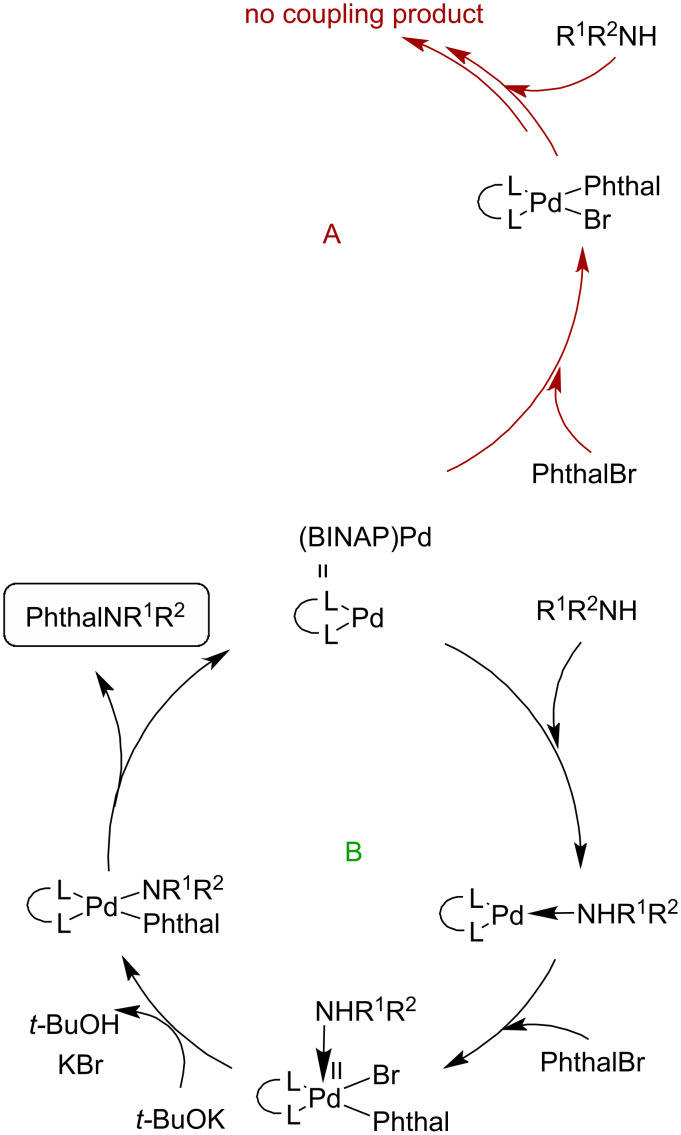
Proposed catalytic cycles for the amination of 4-bromophthalazinones of type **3** (Phthal: phthalazinone, PhthalBr: 4-bromophthalazinone, PhthalNR^1^R^2^: 4-aminophthalazinone).

These results showed that the coordination of the amine to the (BINAP)Pd complex probably leads to the formation of a (BINAP)Pd–amine system (Figure 2), facilitating the oxidative addition of the bromolactam **3a** and is the crucial step in the amination procedure of 4-bromophthalazinones [40].

**Scheme 2:**
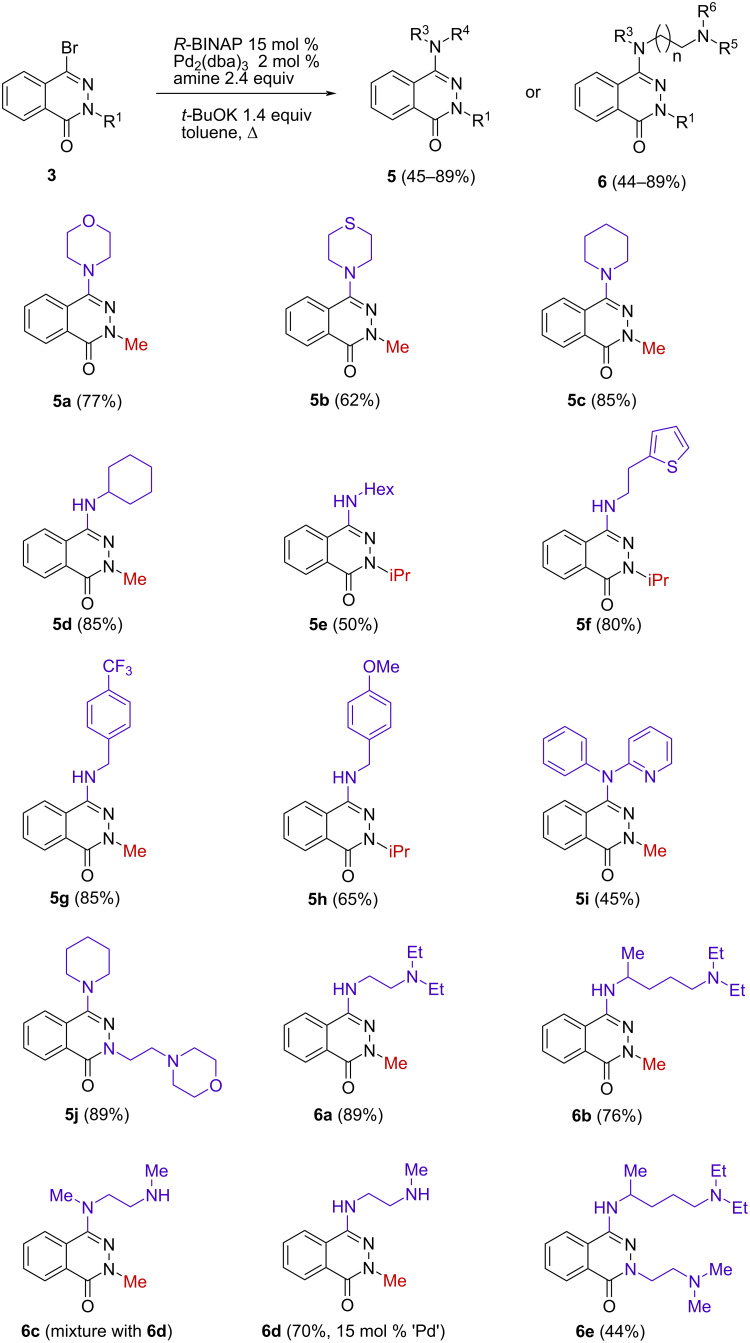
Synthesis of 4-amino- and 4-polyaminophthalazinones **5** and **6** (the yields refer to the isolated compounds).

Based on the results of our initial studies, we next examined the reaction scope of the bromolactams **3** with various amines and polyamines. As can be seen from Scheme 2 the yields of the amination products **5** and **6** were dependent on the nature of the amine as well as on the used phthalazinone. Efficient results of coupling were received using, inter alia, cyclic, aromatic or benzylamines. The reaction of **3a** with piperidine gave a higher yield of the target product **5c** (85%) compared to the use of morpholine (**5a**, 77%), and especially thiomorpholine (**5b**, 62%). In turn, the use of 1-aminohexane (NH_2_Hex) in the reaction with **3b** resulted in a significant decrease in the yield of product **5e** (50%) in comparison with 2-(thiophen-2-yl)ethan-1-amine (**5f**, 80%), cyclohexylamine (**5d**, 85%), and also with the 4-CF_3_ and 4-OMe-benzylamines (**5g**, 85%; **5h**, 65%). Additionally, when using primary amines in the reaction the formation of polysubstituted products was not observed.

On the other hand, *N*-phenylpyridin-2-amine afforded the appropriate compound **5i** in a lower yield as compared with aliphatic amines. Moreover, it turned out that an aminoalkyl substituent attached to the lactam scaffold did not disturb its functionalization at the 4 position by a simple amine. The corresponding product **5j** was obtained in 89% yield.

It was found that also polyamines are suitable reagents in this Pd-coupling reaction. Only the reaction of **3c** with *N*^1^,*N*^1^-diethylpentane-1,4-diamine proceeded in a lower yield and the target derivative **6e** was isolated with 44% yield. In the case of *N*,*N*′-dimethylethylenediamine the substitution of both nitrogen atoms was not observed. Instead, the partial demethylation of the amine occurred. As a result, a mixture of the products **6c** and **6d** (molar ratio **6c**/**6d** = 1:4) was obtained. With the increase in the amount of palladium (15 mol %) the phthalazin-1(2*H*)-one derivative **6d** was obtained as the main product (70%).

#### Complexation behavior of aminophthalazinones with Cu(II) ions

**Figure 3:**
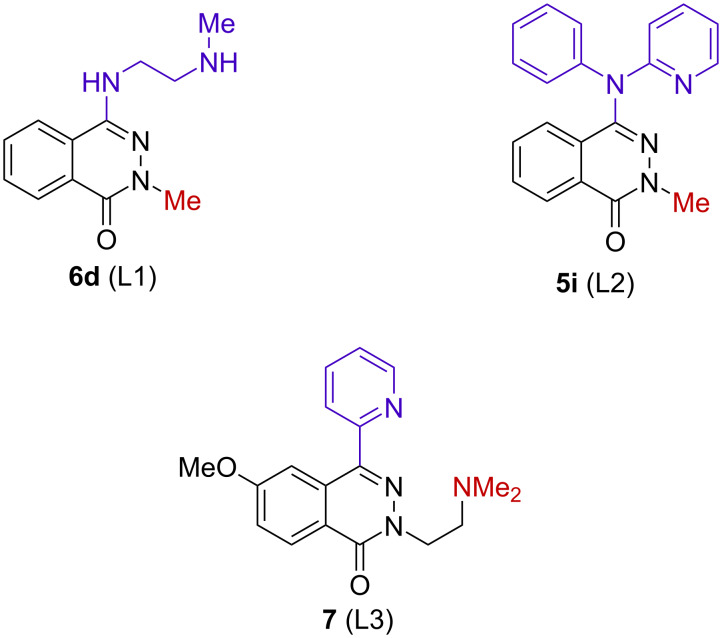
The phthalazinone derivatives that were used to test the complexation of Cu(II) ions.

To conclude the synthetic research, we looked at the potential of aminophthalazinones as polydentate ligands for the synthesis of complexes with metal ions. Examples of the use of phthalazinone and its derivatives in the synthesis of coordination compounds with La(III), Co(II), Cu(II), Mn(II) were extremely interesting [41–42]. Despite the fact that the phthalazinone molecule is potentially a tridentate NNO ligand, so far, in the examples described in the literature the coordination takes place through the oxygen atom [41–42]. Having synthesized 4-aminophthalazinones, we decided to investigate how these compounds behave towards Cu(II) ions. For testing, we chose the derivatives of ethylenediamine **6d**, *N*-phenylpyridin-2-amine **5i**, and additionally the pyridin-2-yl derivative **7** (Figure 3). Compound **7** was synthesized according to the method described by us [43].

All selected compounds (Figure 3) contain some specific structural elements (e.g., an ethylenediamine moiety, similarity to bipyridyl or even to isocyclam skeleton) allowing them to act as ligands and form complexes. In our tests, we hoped that putting in the 4-position of the skeleton a substituent with a donor nitrogen atom could result in the formation of complexes following a different way of coordination with the metal ion than described in the literature [41].

The complexing properties of compounds **5i**, **6d**, and **7** were investigated by mass spectrometry (ESIMS). The spectra were recorded after time intervals of 0 h, 1 h, and 24 h starting from mixing the solutions containing equimolar amounts of the ligand and CuCl_2_. Unexpectedly, compound **6d** (L1) did not show any complexing properties. In all spectra a signal of the same ligand, despite long reaction times, was only observed (ESIMS, negative ion mode, *m*/*z* = 231.1 Da; ESIMS, positive ion mode, *m*/*z* = 233.1 Da (100%)).

In the case of compound **5i** (L2), the formation of two types of complexes was detected: 1) with *m*/*z* = 426.2 Da [(L2)^63^Cu(II)Cl]^+^, 428.2 Da [(L2)^65^Cu(II)Cl]^+^ and 2) with *m*/*z* = 719.5 Da and 721.5 Da, which corresponded to the ^63^Cu^2+^/^65^Cu^2+^ ion complex containing two ligands L2. However, a significant amount of unchanged ligand was also visible. For the ion at *m*/*z* = 719.5 Da, tandem spectra were recorded. The first fragmentation gave ions at *m*/*z* = 329.3 Da (L2 + H^+^) and 391.4 Da (ligand L2 and copper). The further fragmentation of the ion at *m*/*z* = 391.4 Da gave rise to an ion series: 362.4 Da (probably after elimination of HCO), 311.4 Da, 286.3 Da (100%), and 235.4 Da. We did not observe in the tandem spectra the signal after the elimination of copper alone.

**Scheme 3:**
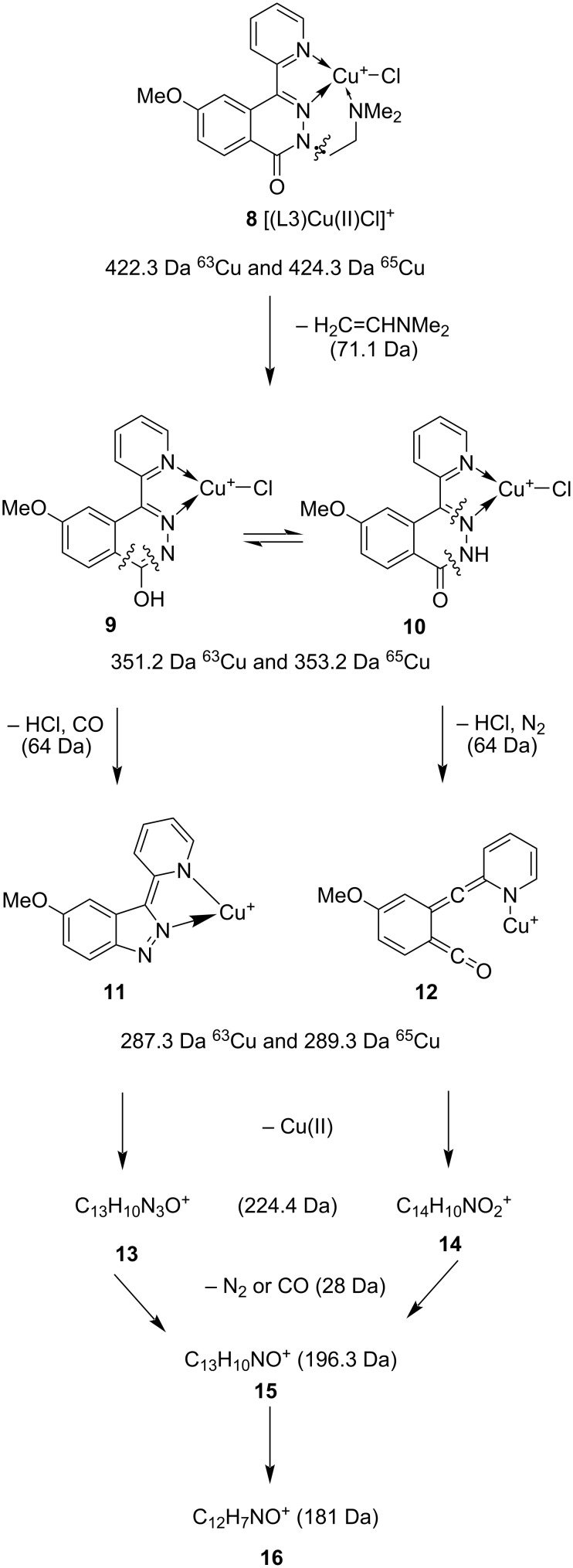
The proposal of the fragmentation pathway of the Cu(II) complex with compound **7**.

**Figure 4:**
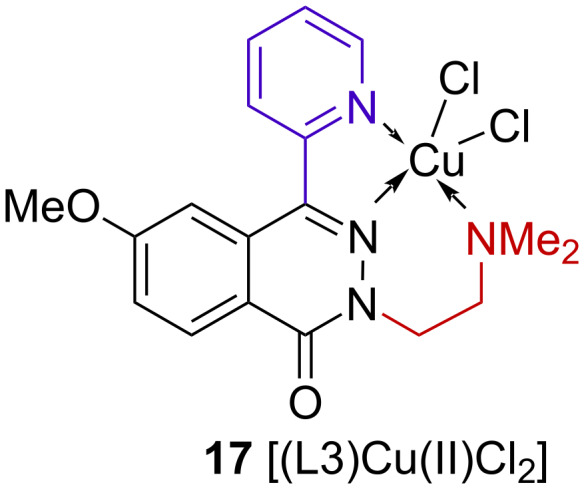
Structure of complex **17**.

Similarly, the ESIMS spectrum of an equimolar mixture of compound **7** (L3) and CuCl_2_ showed the presence of two types of complexes containing one and two phthalazinone ligands: [(L3)Cu(II)Cl]^+^ and [(L3)_2_Cu_2_(II)Cl_3_]^+^. The most abundant peak at *m*/*z* = 422.3 Da corresponded to the complex [(L3)Cu(II)Cl]^+^. The MS/MS fragmentation of the ions at *m*/*z* = 422.3 Da for ^63^Cu and 424.3 Da for ^65^Cu followed the same fragmentation pattern for both ions. The proposal of the fragmentation pathway, based on the X-ray crystal structure of the Cu(II) complex with **7** (L3) (Figure 4, vide infra), is shown in Scheme 3. The MS/MS fragmentation analysis of the [(L3)Cu(II)Cl]^+^ complex **8** (*m*/*z* = 422.3 and 424.3 Da) showed at the first step the loss of an aminoalkyl fragment (C_2_H_3_NMe_2_ = 71.1 Da) to form the ions **9** ⇌ **10** (*m*/*z* = 351.2 and 353.2 Da). Because of the lactam–lactim tautomerism the further complex decomposition can proceed through two fragmentation routes: 1) with the loss of HCl/CO or 2) with the loss of HCl/N_2_ (≈64 Da). In both cases, the pyridazinone moiety undergoes degradation to different ions **11**, **12** with the same *m*/*z* = 287.3 and 289.3 Da. In the next stage, the copper cation is detached to form ions **13** and **14** (224.3 Da) indicating that the copper is well fitted into compound moiety.

Based on the results of X-ray structural analysis of the Cu(II) complex with **7**, it can be assumed, that also in the case of ligand **5i** (L2) the nitrogen atoms of the pyridin-2-yl and azomethin moiety participate in the coordination with Cu(II) ions.

#### Crystallography of complex **17**

**Figure 5:**
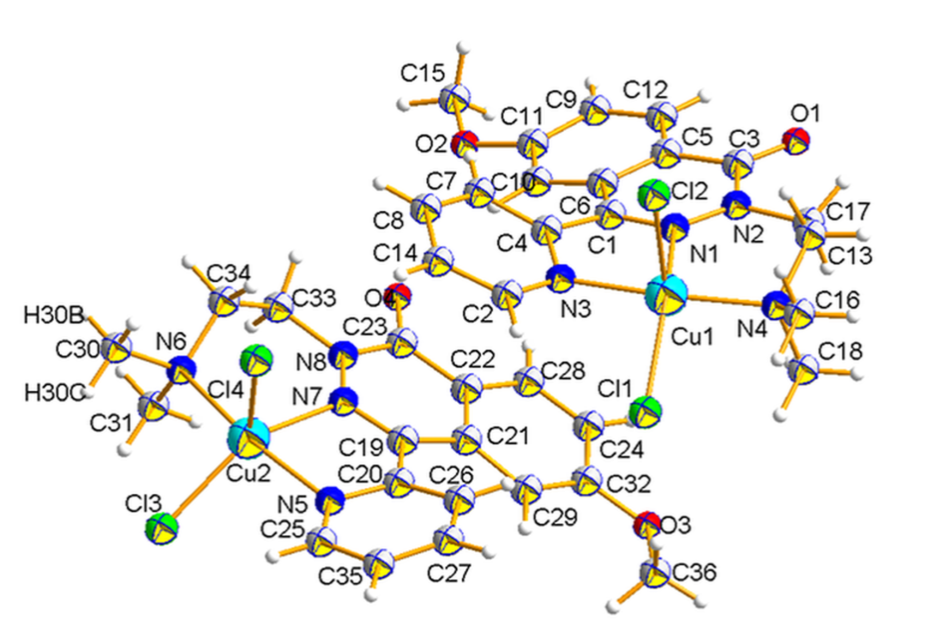
Molecular structure of complex **17** with atom numbering scheme. The anisotropic displacement parameters are shown at the 50% probability level.

The copper(II) complex **17** [(L3)Cu(II)Cl_2_] was synthesized and characterized by X-ray analysis, FTIR and vis–NIR spectroscopy (for details see Supporting Information File 2). The molecular structure of the complex **17** is shown in Figure 4 and Figure 5.

The basic experimental details and selected crystallographic data are summarized in Table 1. For full details on the comparison of experimental and calculated bond lengths and bond angles of complex **17** are provided in Supporting Information File 2, Table S1. The complex **17** crystallizes in the monoclinic *Pc* space group with two molecules A and B in one asymmetric unit. Their geometry was fully optimized in vacuum using the DFT method with the crystal structure coordinates as the input geometry (optimized at the CAM-B3LYP/6–311++G(d,p)/LanL2DZ(Cu) level of theory). However, due to the convergence failure during the geometry optimization of the dimer of molecules A and B, the geometry optimization was performed for the isolated molecules A and B.

**Table 1:** Crystallographic data for complex **17**.

crystal data	complex **17**

empirical formula	C_18_H_20_Cl_2_CuN_4_O_2_
formula weight	458.83
crystal system	monoclinic
space group	*Pc*
*unit cell dimensions*	
*a* (Å)	11.5529(4)
*b* (Å)	11.4432(4)
*c* (Å)	14.3523(5)
α (°)	
β (°)	97.337(3)
γ (°)	
*V* (Å^3^)	1881.87(11)
*Z*	4
*T* (K)	100(2)
*F*(000)	940
*D_x_* (g cm^−3^)	1.620
μ (mm^−1^)	1.466
scan method	Ω-scan
θ range (°)	2.9, 26.5
measured reflections	13642
unique reflections	5023
observed reflections [*I*>2σ(*I*)]	4548
completeness to θ_max_ (%)	99.7
*R* [*I*>2σ(*I*)]	0.0408
*wR* (all data)	0.0943
*S*	1.04
largest diff. peak, hole (e Å^−3^)	−0.45, 0.93

In a solid phase the Cu(II) central ion is five-coordinated by three nitrogen atoms of **7** and two chloride anions. The CuN_3_Cl_2_ coordination center adopts the strongly distorted square pyramidal geometry with the five-coordinate geometry index (τ_5_) of 0.45 and 0.42 for molecules A and B, respectively. In particular, in molecules A and B, the pyridin-2-yl nitrogen atoms (N3 and N5), the azomethine nitrogen atoms (N1 and N7), and the tertiary nitrogen atom of the (2-(dimethylamino)ethyl group (N4 and N6) act as donors of coordination bonds. The coordination sphere of the Cu1 and Cu2 central ions is completed with Cl1, Cl2 and Cl3, Cl4 chloride anions, respectively. In the molecules A and B the pyridin-2-yl substituents are twisted in opposite directions relative to the mean plane of the 1,2-diazine moiety with the dihedral angles equal to 27.61 and 28.71°, respectively, and moreover, the methyl groups of the methoxy substituents are directed opposite and towards to the pyridin-2-yl substituents, respectively. The crystal structure of complex **17** is stabilized with a 3D intermolecular hydrogen bond network (Figure S1a, Supporting Information File 2). Additionally, the complex **17** in a solid state is also stabilized with π–π stacking interactions between the 1,2-diazine moieties and the pyridin-2-yl substituents of the ligands (Figure S1b, Supporting Information File 2).

The FTIR spectrum recorded for complex **17** confirmed the coordination of copper(II) through nitrogen atoms of the pyridin-2-yl substituent, the pyridazin-3-one moiety, and the 2-(dimethylamino)ethyl group. In the vis–NIR spectrum recorded in methanol, based on TD-DFT calculations, the *d*–*d*, metal–ligand charge transfer (MLCT), ligand–metal charge transfer (MLCT), and intraligand charge transfer (ILCT) transitions were identified. The vis–NIR spectroscopy revealed that in complex **17** the metal-to-ligand stoichiometry is equal to 1:1. In turn, cyclic voltammetry was used to investigate the electrochemical behavior of complex **17**. The measurements on a platinum disc electrode suggested that the [(L3)Cu(II)Cl_2_]/[(L3)Cu(I)Cl_2_] redox couple was formed. For more details, see Supporting Information File 2.

### Bioactivity

#### Cytotoxicity analysis – MTT assay

**Figure 6:**
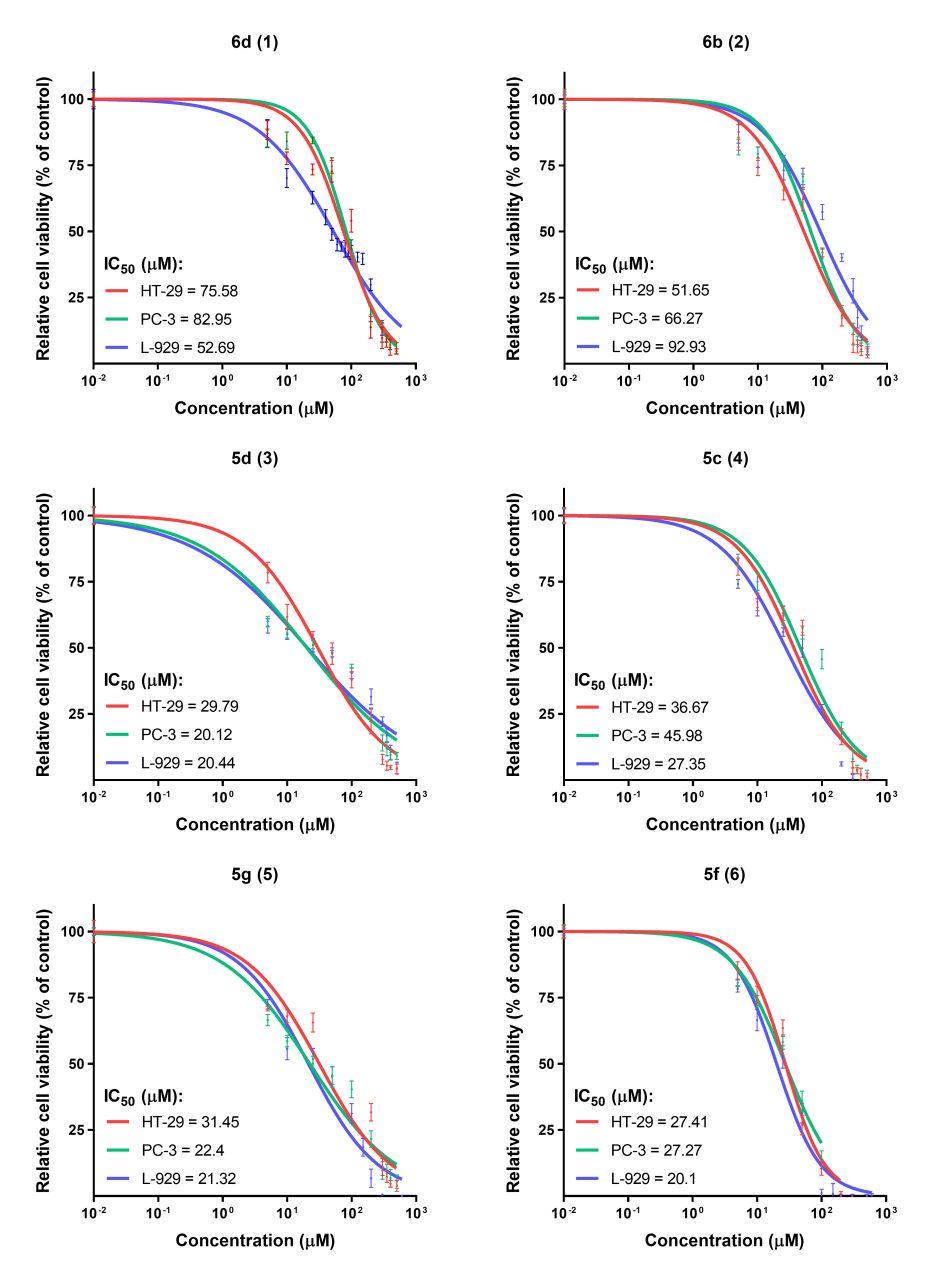
Determination of relative cell viability (% of control) in different cell lines (HT-29; PC-3 and L-929) treated with compounds **6d** (1), **6b** (2), **5d** (3), **5c** (4), **5g** (5), **5f** (6) using the MTT assay. The SEM value is shown for each tested concentration value.

**Figure 7:**
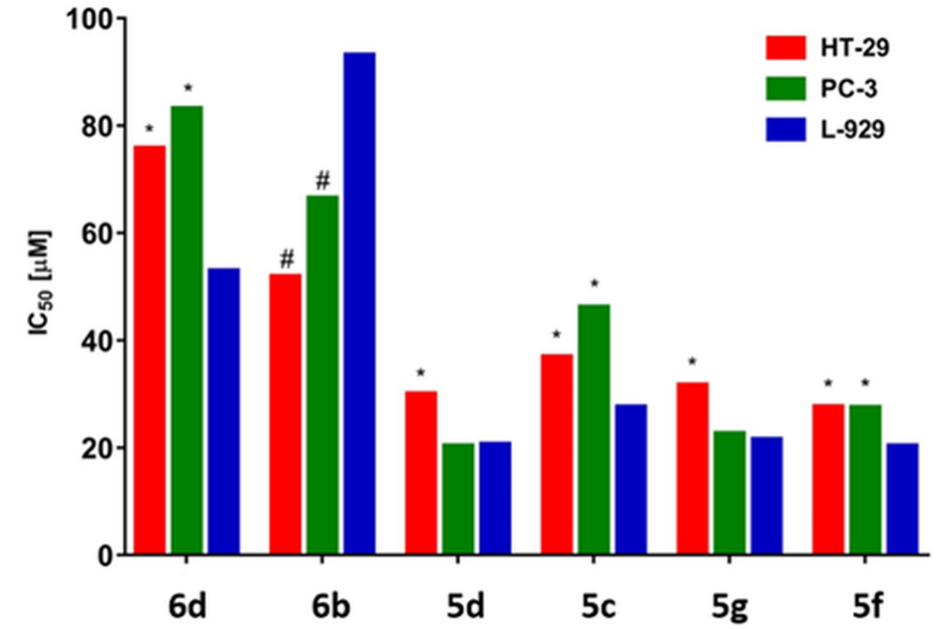
Cytotoxic properties of the phthalazinone derivatives expressed as IC_50_ after 72 h of cell treatment. Values significantly differing from the control cell line (L-929) by ANOVA and Dunnett’s test: * increase and # decrease.

We used the MTT assay to evaluate compounds **5c**, **5d**, **5f**, **5g**, **6b**, and **6d** for their potential activity on cytotoxicity, proliferation and growth of HT-29 (human colon adenocarcinoma cell line), PC-3 (human prostate cancer cell line) and L-929 cells (mouse fibroblast cell line). The results obtained from the MTT assay revealed that the tested phthalazinone derivatives were cytotoxic towards the tested cell lines in the concentration range from 20.1 μM to 92.93 μM (Figure 6 and Figure 7).

The compounds **5d**, **5g** and **5f** exhibited the strongest cytotoxic effects as compared to the other examined compounds. The IC_50_ values for the tested cell lines were respectively: HT-29 (IC_50_ = 29.79 µM; 31.45 µM; 27.41 µM), PC-3 (IC_50_ = 20.12 µM; 22.4 µM; 27.27 µM) and L-929 (IC_50_ = 20.44 µM; 21.32 µM; 20.1µM). However, derivatives **6d** and **5c** appeared to be less cytotoxic against all tested cell lines. The IC_50_ values for the tested cell lines were respectively: HT-29 (IC_50_ = 75.58 µM; 36.67 µM), PC-3 (IC_50_ = 82.95 µM; 45.98 µM) and L-929 (IC_50_ = 52.69 µM; 27.35 µM). Despite the fact that the compounds were cytotoxic to tumor cell lines, they were also similarly or even more cytotoxic to the control line (L-929), which is not a desirable feature for any kind of a new potential pharmaceutical drug.

Among the analyzed compounds only compound **6b** significantly influenced the viability of the tumor cell lines HT-29 (IC_50_ = 51.65 μM) and PC-3 (IC_50_ = 66.27 μM) compared to normal cells L-929 (IC_50_ = 92.93 μM). However, in order to determine further biological utility of compound **6b**, additional research has to be done,

## Conclusion

In conclusion, we have demonstrated an efficient synthesis of 2-substituted (alkyl, aminoalkyl) 4-aminophthalazinones **5** and **6** via the direct bromination of phthalazin-1(2*H*)-one (**1**) with potassium tribromide, followed by the alkylation of 4-bromophthalazinone **2** with methyl iodide, isopropyl iodide or 2-chloro-*N*,*N*-dimethylethylamine and 4-(2-chloroethyl)morpholine and further palladium-catalyzed amination of lactams **3** with aliphatic, aromatic, benzylic, cyclic amines and polyamines. Furthermore, we have demonstrated that some of the phthalazinone derivatives act as ligands and form stable coordination compounds with Cu(II) ions. The results of biological tests showed that the compounds containing an amino- or polyamino-substituent at the 4-position of the phthalazinone moiety could have potential applications as new anticancer agents.

## Supporting Information

File 1 Experimental details, synthetic procedures, and characterization data of new compounds including copies of spectra.

File 2 Experimental details, computational details, X-ray crystallographic data, FTIR and vis–NIR spectra, and cyclic voltammetry for compounds **7** and **17**.

## References

[1] Vila N, Besada P, Costas T, Costas-Lago M C, Terán C (2015). Eur J Med Chem.

[2] Asif M (2019). Chem Int.

[3] Sridhara A M, Reddy K R V, Keshavayya J, Vadiraj S G, Bose P, Ambika D S, Raju C K, Shashidhara S, Raju N H J (2011). J Pharm Res (Gurgaon, India).

[4] Moustafa A H, El-Sayed H A, Abd El-Hady R A, Haikal A Z, El-Hashash M (2016). J Heterocycl Chem.

[5] El-Wahab A H F A, Mohamed H M, El-Agrody A M, El-Nassag M A, Bedair A H (2011). Pharmaceuticals.

[6] Cashman J R, Voelker T, Johnson R, Janowsky A (2009). Bioorg Med Chem.

[7] Cashman J R, Voelker T, Zhang H-T, O’Donnell J M (2009). J Med Chem.

[8] Yamaguchi M, Kamei K, Koga T, Akima M, Kuroki T, Ohi N (1993). J Med Chem.

[9] Procopiou P A, Browning C, Gore P M, Lynn S M, Richards S A, Slack R J, Sollis S L (2012). Bioorg Med Chem.

[10] Procopiou P A, Ford A J, Gore P M, Looker B E, Hodgson S T, Holmes D S, Vile S, Clark K L, Saunders K A, Slack R J (2017). ACS Med Chem Lett.

[11] Medda F, Sells E, Chang H-H, Dietrich J, Chappeta S, Smith B, Gokhale V, Meuillet E J, Hulme C (2013). Bioorg Med Chem Lett.

[12] Poli D, Catarzi D, Colotta V, Varano F, Filacchioni G, Daniele S, Trincavelli L, Martini C, Paoletta S, Moro S (2011). J Med Chem.

[13] Watanabe N, Adachi H, Takase Y, Ozaki H, Matsukura M, Miyazaki K, Ishibashi K, Ishihara H, Kodama K, Nishino M (2000). J Med Chem.

[14] Bollenbach M, Lugnier C, Kremer M, Salvat E, Megat S, Bihel F, Bourguignon J-J, Barrot M, Schmitt M (2019). Eur J Med Chem.

[15] Humphrey J M (2007). Aminophthalazine derivative compounds. WO Pat. Appl..

[16] Herberich B, Cao G-Q, Chakrabarti P P, Falsey J R, Pettus L, Rzasa R M, Reed A B, Reichelt A, Sham K, Thaman M (2008). J Med Chem.

[17] Nomoto Y, Obase H, Takai H, Teranishi M, Nakamura J, Kubo K (1990). Chem Pharm Bull.

[18] Li J, Zhao Y-F, Yuan X-Y, Xu J-X, Gong P (2006). Molecules.

[19] Prime M E, Courtney S M, Brookfield F A, Marston R W, Walker V, Warne J, Boyd A E, Kairies N A, von der Saal W, Limberg A (2011). J Med Chem.

[20] Wang W, Feng X, Liu H-X, Chen S-W, Hui L (2018). Bioorg Med Chem.

[21] Reece P A (1981). Med Res Rev.

[22] Leiro J M, Álvarez E, Arranz J A, Cano E, Orallo F (2004). Int Immunopharmacol.

[23] Graça I, Sousa E J, Costa-Pinheiro P, Vieira F Q, Torres-Ferreira J, Martins M G, Henrique R, Jerónimo C (2014). Oncotarget.

[24] Tanaka S, Tanaka M, Akashi A (1989). Stroke.

[25] Moroi R, Ono K, Saito T, Akimoto T, Sano M (1977). Chem Pharm Bull.

[26] Jost L M, Gschwind H-P, Jalava T, Wang Y, Guenther C, Souppart C, Rottmann A, Denner K, Waldmeier F, Gross G (2006). Drug Metab Dispos.

[27] Dragovich T, Laheru D, Dayyani F, Bolejack V, Smith L, Seng J, Burris H, Rosen P, Hidalgo M, Ritch P (2014). Cancer Chemother Pharmacol.

[28] Wang F, Molina J, Satele D, Yin J, Lim V-S, Adjei A A (2019). Invest New Drugs.

[29] Holló B, Magyari J, Živković-Radovanović V, Vučković G, Tomić Z D, Szilágyi I M, Pokol G, Mészáros Szécsényi K (2014). Polyhedron.

[30] Malinowski Z, Fornal E, Sierocińska B, Czeczko R, Nowak M (2016). Tetrahedron.

[31] Malinowski Z, Fornal E, Nowak M, Kontek R, Gajek G, Borek B (2015). Monatsh Chem.

[32] Vlaar T, Ruijter E, Znabet A, Janssen E, de Kanter F J J, Maes B U W, Orru R V A (2011). Org Lett.

[33] Vlaar T, Mampuys P, Helliwell M, Maes B U W, Orru R V A, Ruijter E (2013). J Org Chem.

[34] Krishnananthan S, Smith D, Wu D-R, Yip S, Gunaga P, Mathur A, Li J (2016). J Org Chem.

[35] Nowak M, Malinowski Z, Jóźwiak A, Fornal E, Błaszczyk A, Kontek R (2014). Tetrahedron.

[36] Patel N R, Castle R N (1973). Phthalazines. Condensed Pyridazines Including Cinnolines and Phthalazines.

[37] Heravi M M, Kheilkordi Z, Zadsirjan V, Heydari M, Malmir M (2018). J Organomet Chem.

[38] Schlummer B, Scholz U (2004). Adv Synth Catal.

[39] Shekhar S, Ryberg P, Hartwig J F, Mathew J S, Blackmond D G, Strieter E R, Buchwald S L (2006). J Am Chem Soc.

[40] Singh U K, Strieter E R, Blackmond D G, Buchwald S L (2002). J Am Chem Soc.

[41] Wang Y, Yang Z-Y, Wang Q, Cai Q-K, Yu K-B (2005). J Organomet Chem.

[42] Öztürk N, Bahçeli S (2012). Suleyman Demirel Univ Fen Bilimleri Enst Derg.

[43] Pakulska W, Malinowski Z, Szcześniak A K, Czarnecka E, Epsztajn J (2009). Arch Pharm (Weinheim, Ger).

