# Supplementary material for: Amino- and polyaminophthalazin-1(2H)-ones: synthesis, coordination properties, and biological activity
Source: Beilstein J Org Chem. 2021 Feb 25;17:558–68. doi: 10.3762/bjoc.17.50 (PMC7934800; doi:10.3762/bjoc.17.50)
Supplement: File 1 — Experimental details, synthetic procedures, and characterization data of new compounds including copies of spectra. [file Beilstein_J_Org_Chem-17-558-s001.pdf]

BEILSTEIN JOURNAL OF ORGANIC CHEMISTRY

Supporting Information

for

Amino- and polyaminophthalazin-1(2*H*)-ones: synthesis, coordination properties, and biological activity

Zbigniew Malinowski, Emilia Fornal, Agata Sumara, Renata Kontek, Karol Bukowski, Beata Pasternak, Dariusz Sroczyński, Joachim Kusz, Magdalena Małecka and Monika Nowak

Beilstein J. Org. Chem. **2021,** *17,* 558–568. doi:10.3762/bjoc.17.50

Experimental details, synthetic procedures, and characterization data of new compounds including copies of spectra

License and Terms: This is a supporting information file under the terms of the Creative Commons Attribution License (https://creativecommons.org/licenses/by/4.0). Please note that the reuse, redistribution and reproduction in particular requires that the author(s) and source are credited and that individual graphics may be subject to special legal provisions.
The license is subject to the *Beilstein Journal of Organic Chemistry* terms and conditions: (https://www.beilstein-journals.org/bjoc/terms)

Table of contents

1. Synthesis S2

1.1 General information S2

1.2 Synthesis of 2-alkylated 4-bromophthalazinones **3** S3

1.3 Synthesis of 4-amino- and 4-polyamino- phthalazinones **5**, **6** S5

2. Biology S13

2.1 Materials and methods S13

2.2 Cell lines S13

2.3 Cytotoxicity analysis – MTT assay S14

2.4 Data analyse S15

3. ESI-MS investigation of phthalazinone complexes S16

3.1 General information S16

3.2 Experimental S16

3.3 ESI-MS spectra S17

4. References S21

5. ^{1}H, ^{13}C NMR and FT-IR spectra of compounds **3**, **5**, **6** S21

1. Chemical syntheses

1.1 General information

Melting points were determined on a Boetius hot stage apparatus and are uncorrected. ^{1}H and ^{13}C NMR spectra were recorded on a Bruker Advance III spectrometer at 600 MHz and 150 MHz, respectively. Chemical shifts (δ_H, δ_C) were quoted in parts per million (ppm), referenced to the signal of TMS or to the appropriate residual solvent peak ($CDCl_3$ at 7.26 ppm or DMSO-d_6 at 2.50 ppm for ^{1}H NMR and $CDCl_3$ at 77.16 ppm or DMSO-d_6 at 39.52 ppm for ^{13}C NMR). Coupling constants (J) were quoted in hertz (Hz). 2D Homonuclear ^{1}H, ^{1}H COSY spectra were used to assign the proton signals. IR spectra were recorded on a Nexus FT-IR spectrometer. LC/HRMS analyses were performed using an Agilent Technologies HPLC 1290 coupled to an Agilent Technologies 6550 Accurate Mass Q-TOF LC–MS mass spectrometer equipped with a JetStream Technology ion source housed in the Department of Pathophysiology, Medical University of Lublin, Poland. Internal mass calibration was enabled; reference ions of m/z = 121.0509 and 922.0098 were used.

ESIMS and ESIMS/MS (used for the investigation of complexing properties) were recorded using a Varian 500-MS LC ion-trap mass spectrometer (Palo Alto, CA, USA). The sample was introduced into the ESIMS source by continuous infusion by means of the instrument syringe pump at a rate of 10 µL min^{-1}. The ESI source was operated at 5.00 kV and the capillary heater was set to 350 °C and the cone voltage within the range 50–150 V. Scanning was performed from m/z = 200 to 1000. Nitrogen (N_2) was used as the drying gas and the nebulizer gas. For fragmentation experiments, mass-selected monoisotopic molecular ions were isolated in the ion trap and collisionally activated using Helium damping gas. All sample solutions were prepared in methanol by Baker.

The analytical thin layer chromatography tests (TLC) were carried out on Sigma-Aldrich (Supelco) silica gel plates (Kieselgel 60 F254, layer thickness 0.2 mm) and the spots were visualized using a UV lamp. The flash column chromatography purifications were performed on Fluka silica gel (Silica gel 60, 0.040–0.063 mm).

All reactions with organopalladium compounds were performed under an argon atmosphere using standard Schlenk techniques. Toluene and 1,4-dioxane were distilled from sodium benzophenone ketyl prior to use. Commercially available reagents: 2-formylbenzoic acid, hydrazine monohydrate, methyl iodide (MeI), isopropyl iodide (iPrI), 2-chloro-*N*,*N*-dimethylethylamine hydrochloride, 4-(2-chloroethyl)morpholine hydrochloride, *N*,*N*-diisopropylethyl amine (DIPEA), *t*-BuOK, Cs_2CO_3, palladium(II) acetate ($Pd(OAc)_2$), 4,5-bis-(diphenylphosphino)-9,9-dimethylxanthene (XantPhos), 2-dicyclohexylphosphino-20-(*N*,*N*-dimethylamino)biphenyl (DavePhos), (oxydi-2,1-phenylene)-bis(diphenylphosphine) (DPEPhos), 2,2′-bis(diphenylphosphino)-1,1′-binaphthalene (*rac*-BINAP), and (*R*)-(+)-2,2′-bis(diphenylphosphino)-1,1′-binaphthalene ((*R*)-BINAP) were purchased from Sigma-Aldrich and used without further purification.

Compounds **1**, **2**, **3a** were obtained as described previously and their characterization data were in agreement with already reported analysis [1].

1.2 Synthesis of 2-alkylated 4-bromophthalazinones **3**

General procedure.

Method A: Analogous as described in [1].

A mixture of 4-bromophthalazin-1(2*H*)-one (**2**, 0.50 g, 2.22 mmol), potassium carbonate (3 equiv) in dry acetone (20 mL) was heated to boiling for 30 min and next alkyl iodide (1.2 equiv) was added. The reaction mixture was heated and stirred under

reflux for 15 h. After cooling to room temperature the formed solid was collected by filtration and washed with DCM (15 mL). The filtrate was concentrated under reduced pressure and then the product was purified by flash chromatography (**3a**) or used in the next step reaction without further purification (**3b**).

Method B: A mixture of 4-bromophthalazin-1(2*H*)-one (**2**, 0.40 g, 1.78 mmol), potassium carbonate (5 equiv) in dry acetone (20 mL) was heated to boiling for 30 min and next the appropriate aminoethyl chloride hydrochloride (1.5 equiv) and sodium iodide (0.1 equiv) were added. The reaction mixture was heated and stirred under reflux for 21 h. After cooling to room temperature the formed solid was collected by filtration and washed with DCM (15 mL). The filtrate was concentrated under reduced pressure and then the product was used in the next step reaction without further purification.

4-Bromo-2-methylphthalazin-1(2*H*)-one (**3a**) [1]. White solid; Yield: 451 mg, 85%
The spectral data is in agreement with the literature values [1].

4-Bromo-2-(propan-2-yl)phthalazin-1(2*H*)-one (**3b**). Beige solid; Yield: 500 mg, 84% (Method A); mp: 93–91 ^{o}C; FT-IR (KBr): ν = 3063, 2981, 2927, 2875, 1655 (C=O), 1575, 1539, 1470, 1331, 1315 cm^{-1}; ^{1}H NMR (600 MHz, $CDCl_3$): δ = 8.44 (dd, *J* = 7.9, 0.8 Hz, 1H, 8 Ar-H), 7.94 (dd, *J* = 8.0, 0.5 Hz, 1H, 5 Ar-H), 7.87–7.84 (m, 1H, 7 Ar-H), 7.81–7.79 (m, 1H, 6 Ar-H), 5.36 (hept, *J* = 6.6 Hz, 1H, C*H*), 1.42 (d, *J* = 6.6 Hz, 6H, 2×Me) ppm; ^{13}C NMR (150 MHz, $CDCl_3$): δ = 158.4 (C=O), 133.6, 132.3, 130.0, 129.0, 128.6, 127.6, 127.6, 49.2, 21.0 ppm; HRMS (ESI) m/z: calcd for $C_{11}H_{12}BrN_2O$ $[M+H]^+$ 267.0127, found 267.0130.

4-Bromo-2-[2-(dimethylamino)ethyl]phthalazin-1(2*H*)-one (**3c**). Beige solid; Yield: 279 mg, 53% (Method B); mp: 39–40 °C; FT-IR (KBr): ν = 3062, 3034, 2980, 2946, 2852, 2818, 2767, 1659 (C=O), 1575, 1542, 1467, 1450, 1435, 1344 cm^{-1}; ^{1}H NMR (600 MHz, $CDCl_3$): δ = 8.43 (d, *J* = 7.9 Hz, 1H, 8 Ar-H), 7.93 (d, *J* = 8.0 Hz, 1H, 5 Ar-H), 7.87–7.85 (m, 1H, Ar-H), 7.82–7.79 (m, 1H, Ar-H), 4.34 (t, *J* = 6.8 Hz, 2H, CH_2), 2.78 (t, *J* = 6.8 Hz, 2H, CH_2), 2.33 (s, 7H, 2×Me) ppm; ^{13}C NMR (150 MHz, $CDCl_3$): δ = 159.0 (C=O), 133.9, 132.6, 130.3, 129.5, 128.5, 127.8, 127.5, 57.3, 48.7, 45.6 ppm; HRMS (ESI) m/z: calcd for $C_{12}H_{15}BrN_3O$ $[M+H]^+$ 296.0393, found 296.0397.

4-Bromo-2-[2-(morpholin-4-yl)ethyl]phthalazin-1(2*H*)-one (**3d**). White solid; Yield: 367 mg, 61% (Method B); mp: 92–94 °C; FT-IR (KBr): ν = 3070, 2993, 2959, 2940, 2892, 2850, 2821, 2794, 1655 (C=O), 1574, 1540, 1470, 1459, 1446, 1428, 1347 cm^{-1}; ^{1}H NMR (600 MHz, $CDCl_3$): δ = 8.39 (dd, *J* = 7.9, 0.9 Hz, 1H, 8 Ar-H), 7.92 (dd, *J* = 8.0, 0.5 Hz, 1H, 5 Ar-H), 7.87–7.84 (m, 1H, Ar-H), 7.81–7.78 (m, 1H, Ar-H), 4.35 (t, *J* = 6.8 Hz, 2H, CH_2), 3.66 (t, *J* = 4.6 Hz, 4H, 2×CH_2), 2.82 (t, *J* = 6.8 Hz, 2H, CH_2), 2.57 (br. s, 4H, 2×CH_2) ppm; ^{13}C NMR (150 MHz, $CDCl_3$): δ = 159.0 (C=O), 133.9, 132.6, 130.2, 129.3, 128.4, 127.9, 127.5, 67.0, 56.5, 53.6, 47.8 ppm; HRMS (ESI) m/z: calcd for $C_{14}H_{17}BrN_3O_2$ $[M+H]^+$ 338.0499, found 338.0502.

1.3 Synthesis of 4-amino- and 4-polyamino- phthalazinones **5**, **6**

General procedure:

The reaction was carried out under an argon atmosphere in an oven-dried sealable Schlenk flask. The flask was charged with $Pd_2(dba)_3$ (2 mol %), (*R*)-BINAP (15 mol %) and freshly distilled toluene (3 mL). The contents of the flask were stirred for 1 minute, then the appropriate amine (2.4 equiv) was added (mixture changed color) and the

reaction mixture was stirred and heated at 90-100 °C for 20 minutes. After this time the mixture was cooled and 4-bromophthalazinone **3** (0.05 g), *t*-BuOK (1.2 equiv) and toluene (2 mL) were added. The resulting mixture was then stirred and heated at 90–100 °C for 20 h. After this time, the mixture was cooled and diluted with chloroform (5 mL). The solid was filtered off (if it appeared), washed with chloroform (2 mL) and the filtrate concentrated. The product was purified by flash chromatography.

2-Methyl-4-(morpholin-4-yl)phthalazin-1(2*H*)-one (**5a**). Yellow solid; Yield: 0.040 g, 77%; mp: 135–136 °C; R_f (AcOEt/hexane 10:0.5) = 0.58; FT-IR (KBr): ν = 3072, 2987, 2959, 2887, 2867, 2849, 1646 (C=O), 1578, 1538, 1451, 1350, 1311 cm^{-1}; ^{1}H NMR (600 MHz, $CDCl_3$): δ = 8.45 (dd, *J* = 7.8, 1.1 Hz, 1H, 8 Ar-H), 7.89–7.87 (m, 1H, 5 Ar-H), 7.78-7.75 (m, 1H, Ar-H), 7.75-7.72 (m, 1H, Ar-H), 3.93–3.92 (m, 4H, 2×CH_2), 3.77 (s, 3H, Me), 3.20–3.19 (m, 4H, 2×CH_2) ppm; ^{13}C NMR (150 MHz, $CDCl_3$): δ = 159.0 (C=O), 149.0, 132.3, 131.2, 129.3, 127.5, 126.5, 124.4, 66.9, 51.6, 39.0 ppm; HRMS (ESI) m/z: calcd for $C_{13}H_{16}N_3O_2$ $[M+H]^+$ 246.1237, found 246.1240.

2-Methyl-4-(thiomorpholin-4-yl)phthalazin-1(2*H*)-one (**5b**). Orange solid; Yield: 0.034 g, 62%; mp: 132–134 °C; R_f (Hexane/AcOEt 2:1) = 0.30; FT-IR (KBr): ν = 2967, 2959, 2914, 2844, 1645 (C=O), 1578, 1540, 1448, 1452, 1348 cm^{-1}; ^{1}H NMR (600 MHz, $CDCl_3$): δ = 8.44 (d, *J* = 7.6 Hz, 1H, 8 Ar-H), 7.82 (d, *J* = 8.0 Hz, 1H, 5 Ar-H), 7.78–7.76 (m, 1H, Ar-H), 7.75–7.72 (m, 1H, Ar-H), 3.76 (s, 3H, Me), 3.46–3.45 (m, 4H, 2×NCH_2), 2.89–2.87 (m, 4H, 2×SCH_2) ppm; ^{13}C NMR (150 MHz, $CDCl_3$): δ = 159.0 (C=O), 149.8, 132.4, 131.2, 129.2, 127.5, 126.8, 124.3, 53.3, 39.1, 27.8 ppm; HRMS (ESI) m/z: calcd for $C_{13}H_{16}N_3OS$ $[M+H]^+$ 262.1009, found 262.1012.

2-Methyl-4-(piperidin-1-yl)phthalazin-1(2*H*)-one (**5c**). Cream solid; Yield: 0.043 g, 85%; mp: 92–93 ^{o}C (lit. 95-96 ^{o}C [4]); R_f (AcOEt/hexane 10:0.5) = 0.80; FT-IR (KBr): ν = 3069, 2992, 2968, 2940, 2919, 2847, 2822, 1649 (C=O), 1581, 1542, 1465, 1442, 1342 cm^{-1}; ^{1}H NMR (600 MHz, $CDCl_3$): δ = 8.42 (dd, *J* = 7.8, 0.8 Hz, 1H, 8 Ar-H), 7.87 (d, *J* = 7.9 Hz, 1H, 5 Ar-H), 7.76–7.73 (m, 1H, Ar-H), 7.72–7.69 (m, 1H, Ar-H), 3.75 (s, 3H, Me), 3.13–3.11 (m, 4H, 2×NCH_2), 1.80–1.77 (m, 4H, 2×CH_2), 1.67–1.64 (m, 2H, CH_2) ppm; ^{13}C NMR (150 MHz, $CDCl_3$): δ = 159.0 (C=O), 150.4, 132.1, 130.9, 129.2, 127.3, 127.1, 124.8, 52.4, 39.0, 26.1, 24.5 ppm; HRMS (ESI) m/z: calcd for $C_{14}H_{18}N_3O$ $[M+H]^+$ 244.1444, found 244.1447.

4-(Cyclohexylamino)-2-methylphthalazin-1(2*H*)-one (**5d**). White solid; Yield: 0.030 g, 85%; mp: 84–86 ^{o}C; R_f (AcOEt/hexane 1:1) = 0.46; FT-IR (KBr): ν = 3354 (N-H), 2926, 2850, 1633 (C=O), 1622, 1560, 1537, 1480, 1448, 1359 cm^{-1}; ^{1}H NMR (600 MHz, $CDCl_3$): δ = 8.47 (d, *J* = 7.2 Hz, 1H, 8 Ar-H), 7.78–7.68 (m, 2H, 6,7 Ar-H), 7.57 (d, *J* = 7.0 Hz, 1H, 5 Ar-H), 4.22 (d, *J* = 5.7 Hz, 1H, NH), 3.82–3.77 (m, 1H, C*H*), 3.72 (s, 3H, Me), 2.16 (d, *J* = 11.3 Hz, 2H, CH_2), 1.79 (d, *J* = 13.4 Hz, 2H, CH_2), 1.69 (d, *J* = 12.9 Hz, 1H, CH_2), 1.45 (q, *J* = 11.9 Hz, 2H, CH_2), 1.30–1.23 (m, 3H, CH_2) ppm;
^{13}C NMR (150 MHz, $CDCl_3$): δ = 157.8 (C=O), 143.2, 132.1, 130.9, 128.7, 127.7, 124.8, 120.8, 49.9, 39.0, 33.1, 26.0, 25.0 ppm; HRMS (ESI) m/z: calcd for $C_{14}H_{20}N_3O$ $[M+H]^+$ 258.1601, found 258.1605.

4-(Hexylamino)-2-isopropylphthalazin-1(2*H*)-one (**5e**). White solid; Yield: 0.025 g, 50%; mp: 86–87 ^{o}C; R_f (AcOEt/hexane 3:1) = 0.68; FT-IR (KBr): ν = 3345 (N-H), 3075, 2993, 2981, 2954, 2956, 2853, 1631 (C=O), 1571, 1527, 1475, 1454, 1363, 1344, 1331 cm^{-1}; ^{1}H NMR (600 MHz, $CDCl_3$): δ = 8.49 (dd, *J* = 7.1, 2.3 Hz, 1H, 8 Ar-H), 7.76–7.71

(m, 2H, 6,7 Ar-H), 7.59 (dd, J = 7.0, 1.8 Hz, 1H, 5 Ar-H), 5.43–5.36 (m, 1H, CH), 4.38 (br. s, 1H, NH), 3.40 (t, J = 7.2 Hz, 2H, CH_2), 1.71 (p, J = 7.3 Hz, 2H, CH_2), 1.44 (p, J = 6.8 Hz, 2H, CH_2), 1.37 (d, J = 6.7 Hz, 6H, 2×Me), 1.32–1.27 (m, 4H, CH_2), 0.89 (t, J = 6.8 Hz, 3H, Me) ppm; ^{13}C NMR (150 MHz, $CDCl_3$): δ = 157.0 (C=O), 143.9, 132.2, 130.8, 128.7, 128.0, 124.2, 120.6, 47.7, 42.2, 31.8, 29.5, 29.3, 27.3, 22.7, 20.7, 14.1 ppm; HRMS (ESI) m/z: calcd for $C_{17}H_{26}N_3O$ $[M+H]^+$ 316.2383, found 316.2387.

2-Isopropyl-4-{[2-(thiophen-2-yl)ethyl]amino}phthalazin-1(2*H*)-one (**5f**). Yellow solid; Yield: 0.047 g, 80%; mp: 121–123 °C; R_f (AcOEt/hexane 10:0.5) = 0.56; FT-IR (KBr): ν = 3368 (N-H), 3107, 3070, 2970, 2931, 1633 (C=O), 1570, 1535, 1477, 1449, 1431, 1344, 1315 cm^{-1}; ^{1}H NMR (600 MHz, $CDCl_3$): δ = 8.50–8.48 (m, 1H, Ar-H), 7.74–7.71 (m, 2H, Ar-H), 7.54–7.53 (m, 1H, Ar-H), 7.18 (dd, J = 5.1, 0.9 Hz, 1H, Thio-H), 6.97 (dd, J = 5.0, 3.5 Hz, 1H, Thio-H), 6.89 (d, J = 2.7 Hz, 1H, Thio-H), 5.41 (hept, J = 6.6 Hz, 1H, CH), 4.67 (t, J = 5.4 Hz, 1H, NH), 3.72–3.69 (m, 2H, CH_2), 3.26 (t, J = 6.7 Hz, 2H, CH_2), 1.40 (d, J = 6.6 Hz, 6H, 2×Me) ppm; ^{13}C NMR (150 MHz, $CDCl_3$): δ = 157.0 (C=O), 143.4, 142.0, 132.3, 130.9, 128.8, 128.0, 127.0, 125.3, 124.1, 123.9, 120.6, 47.7, 43.5, 29.3, 20.8.ppm; HRMS (ESI) m/z: calcd for $C_{17}H_{20}N_3OS$ $[M+H]^+$ 314.1322, found 314.1326.

2-Methyl-4-{[4-(trifluoromethyl)benzyl]amino}phthalazin-1(2*H*)-one (**5g**). White solid; Yield: 0.030 g, 85%; mp: 216–218 °C; R_f (AcOEt/hexane 10:0.5) = 0.58; FT-IR (KBr): ν = 3360 (N-H), 3075, 2931, 1631 (C=O), 1571, 1535, 1480, 1431, 1351, 1335 cm^{-1}; ^{1}H NMR (600 MHz, CDCl3): δ = 8.49–8.48 (m, 1H, 8 Ar-H), 7.77–7.75 (m, 2H, Ar-H), 7.64–7.61 (m, 3H, Ar, Ph-H), 7.55 (d, J = 7.8 Hz, 2H, Ph-H), 4.77 (s, 1H, NH), 4.63 (d, J = 5.2 Hz, 2H, CH_2), 3.71 (s, 3H, Me) ppm; ^{13}C NMR (150 MHz, $CDCl_3$): δ = 158.0

(C=O), 143.6, 143.3, 132.4, 131.3, 129.7 (q, J = 32.4 Hz), 128.6, 128.3, 127.8, 125.5 (q, J = 3.8 Hz) 124.5, 124.2 (q, J = 271.9), 121.0, 45.7, 38.9 ppm; HRMS (ESI) m/z: calcd for $C_{17}H_{15}F_3N_3O$ $[M+H]^+$ 334.1162, found 334.1163.

2-Isopropyl-4-[(4-methoxybenzyl)amino]phthalazin-1(2*H*)-one (**5h**). Light yellow solid; Yield: 0.025 g, 65%; mp: 108–110 °C; R_f (AcOEt/hexane 3:1) = 0.64; FT-IR (KBr): ν = 3339 (N-H), 3104, 3076, 2968, 2931, 2832, 1629 (C=O), 1568, 1530, 1514, 1468, 1426, 1349, 1330 cm^{-1}; ^1H NMR (600 MHz, $CDCl_3$): δ = 8.48 (dd, J = 6.3, 3.0 Hz, 1H, 8 Ar-H), 7.72–7.71 (m, 2H, Ar-H), 7.60 (dd, J = 6.3, 2.9 Hz, 1H, 5 Ar-H), 7.37 (d, J = 8.6 Hz, 2H, Ph-H), 6.90 (d, J = 8.6 Hz, 2H, Ph-H), 5.38 (hept, J = 6.6 Hz, 1H, C*H*), 4.67 (t, J = 4.6 Hz, 1H, NH), 4.51 (d, J = 5.2 Hz, 2H, C*H*$_2$), 3.81 (s, 3H, OMe), 1.35 (d, J = 6.6 Hz, 6H, 2×Me) ppm; ^{13}C NMR (150 MHz, $CDCl_3$): δ = 159.0 (C=O), 157.1, 143.6, 132.2, 131.4, 130.9, 129.4, 128.8, 128.0, 124.1, 120.8, 114.0, 55.3, 47.9, 45.9, 20.7 ppm; HRMS (ESI) m/z: calcd for $C_{19}H_{22}N_3O_2$ $[M+H]^+$ 324.1707, found 324.1712.

2-Methyl-4-[phenyl(pyridin-2-yl)amino]phthalazin-1(2*H*)-one (**5i**). Yellow solid; Yield: 0.011 g, 45%; mp: 160–163 °C; R_f (AcOEt/hexane 3:1) = 0.44; FT-IR (KBr): ν = 3062, 2925, 1648 (C=O); 1578, 1539, 1470, 1431, 1345, 1332 cm^{-1}; ^1H NMR (600 MHz, DMSO-d_6): δ = 8.45 (d, J = 8.0 Hz, 1H, 8 Ar-H), 8.15 (d, J = 4.4 Hz, 1H, 6 Py-H), 7.73–7.71 (m, 1H, Ar-H), 7.66 – 7.61 (m, 2H, Py-H), 7.52–7.50 (m, 1H, Py-H), 7.37–7.34 (m, 2H, Ph-H), 7.31 (d, J = 7.8 Hz, 2H, Ph-H), 7.20–7.18 (m, 1H, Ph-H), 6.85 (d, J = 8.4 Hz, 1H), 6.83–6.81 (m, 1H, Py-H), 3.78 (s, 3H, Me) ppm; ^{13}C NMR (150 MHz, $CDCl_3$): δ = 159.7 (C=O), 158.5, 148.5, 144.6, 144.0, 137.7, 133.0, 131.4, 129.6, 129.5, 129.3, 127.3, 125.5, 125.3, 125.1, 116.6, 111.9, 39.6 ppm; HRMS (ESI) m/z: calcd for $C_{20}H_{17}N_4O$ $[M+H]^+$ 329.1397, found 329.1401.

2-[2-(Morpholin-4-yl)ethyl[-4-(piperidin-1-yl)phthalazin-1(2*H*)-one (**5j**). Cream solid; Yield: 0.015 g, 89%; mp: 103–104 °C; R_f (AcOEt/MeOH 10:1) = 0.40; FT-IR (KBr): ν = 3073, 2979, 2956, 2939, 2919, 2895, 2869, 2851, 2838, 2816, 1649 (C=O), 1583, 1544, 1459, 1437, 1350, 1331 cm^{-1}; ^1H NMR (600 MHz, $CDCl_3$): δ = 8.42 (d, *J* = 7.8 Hz, 1H, 8 Ar-H), 7.87 (d, *J* = 8.0 Hz, 1H, 5 Ar-H), 7.77–7.75 (m, 1H, Ar-H), 7.73–7.70 (m, 1H, Ar-H), 4.30 (t, *J* = 6.9 Hz, 2H, $CH_{2\ (ethylene)}$), 3.69 (t, *J* = 4.5 Hz, 4H, CH_2), 3.12 (br. s, 4H, CH_2), 2.81 (t, *J* = 6.9 Hz, 2H, $CH_{2\ (ethylene)}$), 2.57 (br. s, 4H, CH_2), 1.80–1.77 (m, 4H, CH_2), 1.68–1.66 (m, 2H, CH_2) ppm; ^{13}C NMR (150 MHz, $CDCl_3$): δ = 158.8 (C=O), 150.3, 132.2, 130.9, 129.2, 127.4, 126.9, 124.7, 67.0, 56.4, 53.6, 52.4, 47.4, 26.0, 24.5 ppm; HRMS (ESI) m/z: calcd for $C_{19}H_{27}N_4O_2$ $[M+H]^+$ 343.2128, found 343.2134.

4-{[2-(Diethylamino)ethyl]amino}-2-methylphthalazin-1(2*H*)-one (**6a**). Yellow solid; Yield: 0.062 g, 89%; mp: 118–119 °C; R_f (AcOEt/MeOH 1:1) = 0.1; FT-IR (KBr): ν = 3344 (N-H), 3066, 2971, 2932, 2804, 1635 (C=O), 1575, 1555, 1537, 1478, 1432, 1360, 1333 cm^{-1}; ^1H NMR (600 MHz, CDCl3): δ = 8.47 (dd, *J* = 7.8, 1.3 Hz, 1H, 8 Ar-H), 7.77–7.72 (m, 2H, Ar-H), 7.62 (d, *J* = 7.7 Hz, 1H, 5 Ar-H), 5.48 (br. s, 1H, NH), 3.73 (s, 3H, Me), 3.38–3.35 (m, 2H, CH_2), 2.77 (t, *J* = 5.8 Hz, 2H, CH_2), 2.61 (q, *J* = 7.1 Hz, 4H, 2×CH_2), 1.07 (t, *J* = 7.1 Hz, 6H, 2×Me) ppm; ^{13}C NMR (150 MHz, $CDCl_3$): δ = 158.0 (C=O), 144.8, 132.5, 131.2, 128.6, 127.6, 125.0, 121.7, 51.2, 47.0, 39.0, 38.7, 11.5 ppm; HRMS (ESI) m/z: calcd for $C_{15}H_{23}N_4O$ $[M+H]^+$ 275.1866, found 275.1870.

4-{[5-(Diethylamino)pentan-2-yl]amino}-2-methylphthalazin-1(2*H*)-one (**6b**). Light yellow solid; Yield: 0.050 g, 76%; mp: 84–86 °C; R_f (AcOEt/MeOH 10:0.5) = 0.06; FT-

IR (KBr): ν = 3352 (N-H), 3106, 3072, 2968, 2928, 2867, 2796, 2804, 1634 (C=O), 1572, 1556, 1530, 1478, 1453, 1367, 1335 cm^{-1}; ^{1}H NMR (600 MHz, $CDCl_3$): δ = 8.47–8.45 (m, 1H, 8 Ar-H), 7.74–7.70 (m, 2H, Ar-H), 7.63–7.60 (m, 1H, 5 Ar-H), 4.58 (br. s, 1H, NH), 4.03–3.99. (m, 1H, C*H*), 3.72 (s, 3H, Me), 2.52 (q, *J* = 7.1 Hz, 4H, 2×CH_2), 2.47–2.45 (m, 2H, CH_2), 1.69–1.56 (m, 4H, 2×CH_2), 1.28 (d, *J* = 6.4 Hz, 3H, Me), 1.01 (t, *J* = 7.2 Hz, 6H, 2×Me) ppm; ^{13}C NMR (150 MHz, $CDCl_3$): δ = 157.8 (C=O), 143.7, 132.2, 131.0, 128.8, 127.8, 125.0, 121.1, 53.0, 47.0, 47.0, 39.1, 35.0, 24.0, 20.7, 11.6 ppm; HRMS (ESI) m/z: calcd for $C_{18}H_{29}N_4O$ [M+H]$^+$ 317.2336, found 317.2340.

2-Methyl-4-{methyl[2-(methylamino)ethyl]amino}phthalazin-1(2*H*)-one (**6c**) (The crude mixture with **6d** after reaction. Molar ratio **6c**/**6d** ca. 1:4).^{1}H NMR (600 MHz, $CHCl_3$): δ = 8.47–8.46 (m, 1H, Ar-H **6d**), 8.44 (d, *J* = 8.0 Hz, 1H, 8Ar-H, **6c**), 8.03 (d, *J* = 8.3 Hz, 1H, 5 Ar-H, **6c**), 7.78–7.70 (m, 3H, Ar-H, **6c**, **6d**), 7.67 (d, *J* = 8.2 Hz, 1H, 5 Ar-H, **6d**), 5.18 (s, 1H, NH, **6d**), 3.76 (s, 1H, Me, **6c**), 3.73 (s, 3H, Me, **6d**), 3.46–3.43 (m, 2H, CH_2, **6d**), 3.30 (t, *J* = 6.2 Hz, 1H, CH_2, **6c**), 2.94–2.92 (m, 2H, CH2, **6d**), 2.89–2.87 (m, 2H, CH2, Me, **6c**), 2.49 (s, 3H, Me, **6d**), 2.47 (s, 1H, Me, **6c**) ppm.

2-Methyl-4-{[2-(methylamino)ethyl]amino}phthalazin-1(2*H*)-one (**6d**). Orange solid; Yield: 0.034 g, 70%; mp: 153–155 ^{o}C; R_f (MeOH) = 0.10; FT-IR (KBr): ν = 3277, 3229 (N-H); 3080, 2974, 2932, 2883, 2841, 2795, 1636 (C=O), 1578, 1538, 1470, 1437, 1351, 1335 cm^{-1}; ^{1}H NMR (600 MHz, $CDCl_3$): δ = 8.47–8.45 (m, 1H, 8 Ar-H), 7.76–7.71 (m, 2H, Ar-H), 7.68–7.67 (m, 1H, 5 Ar-H), 5.18 (br. s, 1H, NH), 3.73 (s, 3H, Me), 3.46–3.43 (m, 2H, CH_2), 2.98–2.89 (m, 2H, CH_2), 2.49 (s, 3H, Me), 1.59 (br. s, 1H, NH + H_2O) ppm; ^{13}C NMR (150 MHz, $CDCl_3$): δ = 158.1, 144.7, 132.3, 131.2, 128.7, 127.7,

124.9, 121.4, 50.4, 41.1, 39.0, 36.2 ppm; HRMS (ESI) m/z: calcd for $C_{12}H_{17}N_4O$ $[M+H]^+$ 233.1397, found 233.1400.

4-{[5-(Diethylamino)pentan-2-yl]amino}-2-[2-(dimethylamino)ethyl]phthalazin-1(2*H*)-one (**6e**). Cream solid; Yield: 0.0275 g, 44%; mp: 58–60 °C; R_f (MeOH) = 0.10; FT-IR (KBr): ν = 3342 (N-H), 3075, 2965, 2815, 2778, 1629 (C=O), 1570, 1525, 1477, 1452, 1359 cm^{-1}; ^1H NMR (600 MHz, $CDCl_3$): δ = 8.47–8.45 (m, 1H, 8 Ar-H), 7.74–7.70 (m, 2H, Ar-H), 7.60 (d, *J* = 7.7 Hz, 1H, 5 Ar-H), 4.59 (d, *J* = 6.6 Hz, 1H, NH), 4.33–4.29 (m, 1H, CH_2), 4.23–4.18 (m, 1H, CH_2), 4.01–3.96 (m, 1H, C*H*), 2.79–2.76 (m, 2H, CH_2), 2.53 (q, *J* = 7.1 Hz, 4H, 2×CH_2), 2.45 (t, *J* = 6.9 Hz, 2H, CH_2), 2.34 (s, 6H, 2×Me), 1.70–1.57 (m, 4H, CH_2), 1.28 (d, *J* = 6.4 Hz, 3H, Me), 1.01 (t, *J* = 7.1 Hz, 6H, 2×Me) ppm; ^{13}C NMR (150 MHz, $CDCl_3$): δ = 157.6, 143.5, 132.1, 130.9, 128.7, 127.8, 124.7, 120.9, 57.3, 52.9, 48.1, 46.9, 46.8, 45.7, 34.7, 23.9, 20.6, 11.4 ppm; HRMS (ESI) m/z: calcd for $C_{21}H_{36}N_5O$ $[M+H]^+$ 374.2914, found 374.2920.

2. Biology

2.1 Materials and methods:

Trypsin-EDTA and all media (RPMI 1640, DMEM/F-12K and IMDM) were purchased from Biowest (CytoGen, Poland). Buffered saline (PBS), penicillin-streptomycin solution stabilized, fetal bovine serum (FBS), amino acids solution (MEM), β-mercaptoethanol, dimethyl sulfoxide (DMSO) and MTT 3-(4,5-dimethylthiazol-2-yl)-2,3-diphenyltetrazolium bromide were supplied by Sigma Aldrich Chemical Co (USA).

2.2 Cell lines

All tested cell lines were provided by American Type Culture Collection (ATCC, Rockville, Manassas,VA, USA). Two adherent human tumor cell lines were employed to perform the analyses: colorectal adenocarcinoma (HT-29; ATCC® HTB-38™) and prostate cancer (PC3; ATCC® CRL-1435™). In addition, the mouse fibroblast cell line (L-929; ATCC® CCL-1™) was used as a model for non-cancer cells.

The HT-29 cells were cultured in RPMI-1640 medium containing 2 mM L-glutamine, 10 mM HEPES, 1 mM sodium pyruvate, 4500 mg/L glucose and 1500 mg/L sodium bicarbonate, supplemented with 10% (v/v) fetal bovine serum (FBS), 1% (v/v) penicillin/streptomycin and 1% (v/v) MEM amino acid’s solution.

The PC-3 cells were cultivated in DMEM/F12 medium containing 2.5 mM L-glutamine, 15 mM HEPES, 0.5 mM sodium pyruvate and 1200 mg/L sodium bicarbonate glutamine, supplemented with 1% (v/v) penicillin/streptomycin and 10% (v/v) fetal bovine serum (FBS).

The L-929 was cultivated in IMDM Medium with 4 mM L-glutamine, 4500 mg/L glucose, and 1500 mg/L sodium bicarbonate supplemented with 10% (v/v) fetal bovine serum (FBS), 1% (v/v) penicillin/streptomycin and 1% (v/v) β-mercaptoethanol.

Cells were maintained in a humidified incubator at 37 °C in a 5% CO_2 atmosphere and were regularly screened for mycoplasma contamination. Exponential growth of the cells was controlled by their routine passaging at 90% confluence three times a week using 0.025% trypsin/EDTA.

2.3 Cytotoxicity analysis – MTT assay:

The viability of the cells is associated with their mitochondrial integrity and capacity. For that reason, in the MTT microplate assay with 3-(4,5-dimethylthiazol-2-yl)-2,3-diphenyltetrazolium bromide, first described by Mosmann [5], only viable cells with intact metabolism have the ability to incorporate the yellow, water-soluble tetrazolium salt which is subsequently reduced by the mitochondrial enzyme succinate dehydrogenase to the violet-blue formazan compound. After dissolving the formazan crystals in an organic solvent, e.g., dimethyl sulfoxide (DMSO), the absorbance of the formazan solution can be measured spectrophotometrically at 570 nm [6].

In our research, the MTT assay was used to describe the effect of the tested compounds on cytotoxicity, proliferation and growth of HT-29, PC-3 and L-929 cells. Before treatments with the tested compounds the suspension of 8×10^3 cells in 100 µL medium was transferred to each well of a standard 96-well microplate. Subsequently, the plates were incubated for 24 h (5% CO_2; 37 °C) to ensure cell growth.

Afterwards, the examined substances at 10 different concentrations were added in 2–11 columns (5; 10; 25; 50; 100; 200; 300; 350; 400 and 500 µM, respectively) in a volume of 100 µL medium per well. These concentrations were obtained diluting the compounds first in DMSO and then in medium, which were subsequently used to treat cells cultures in a final DMSO concentration between 0.1 and 1% per well [6] (Da Violante et. al. 2002). As control, the first and last columns were filled only with medium

without studied compounds. Furthermore, blanks (wells without cells) were also included in each column.

The plates were then incubated for 72 h (37 °C; 5% CO_2). Subsequently, 25 µL of fresh MTT solution (5 mg/mL in PBS) was added to each well and the microplates were incubated in a humidified atmosphere for additional 3 h (37 °C; 5% CO_2). After the incubation, the MTT solution was replaced by 100 µL of DMSO in order to dissolve formazan complexes and absorbance was immediately measured (microplate reader PowerWaveXS BioTek Instruments, Inc., USA) at 570 nm.

2.4 Data analysis:

The IC_{50} value (concentration of an inhibitor which reduces the absorbance level in the treated cells in comparison to untreated cells by half) representing the cytotoxicity of a substance was evaluated for each studied compound using the GraphPad Prism 7.0 software system (GraphPad Prism Software Inc., USA). To determinate statistical significance between the results one-way variance analysis (ANOVA) was used. The samples homogeneity was tested using Bartlett's test, followed by Dunnett's test. All data are presented as the mean of the replicates from three independent experiments, with a significance level of $p \leq 0.05$.

3. ESIMS investigation of phthalazinone complexes

3.1 General Information

ESIMS and ESIMS/MS (used for the investigation of complexing properties) were recorded using a Varian 500-MS LC ion-trap mass spectrometer (Palo Alto, CA, USA). The sample was introduced into the ESIMS source by continuous infusion by means of the instrument syringe pump at a rate of 10 μL min^{-1}. The ESI source was operated at 5.00 kV and the capillary heater was set to 350 °C. The cone voltage within the range 50–150 V. Scanning was performed from *m/z* = 200 to 1000. Nitrogen (N_2) was used as the drying gas and nebulizer gas. For fragmentation experiments, mass-selected monoisotopic molecular ions were isolated in the ion trap and collisionally activated using Helium damping gas. All sample solutions were prepared in methanol by Baker. The experiments were performed in the positive ion-mode.

3.2 Experimental

Methanolic solutions of the ligands **5i** or **6d** or **7** and $CuCl_2{\cdot}2H_2O$ (c = 10^{-3} mol/dm^3 each) were freshly prepared prior to analysis. Next, the solutions were diluted with methanol up to 10^{-4} mol/dm^3. The solutions prepared this way were then mixed in 1:1 (v/v, ligand / copper salt) at ambient temperature and analyzed by ESIMS. In case of ligand **6d** absence of complex was observed, due to that the sample was further mixed for 24 hours.

3.3 ESIMS spectra

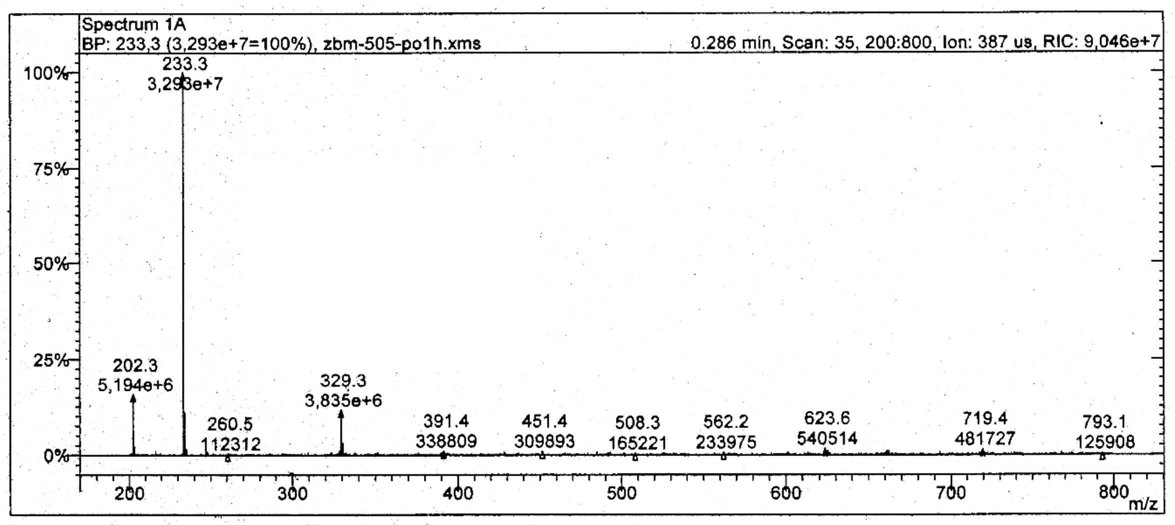

a)

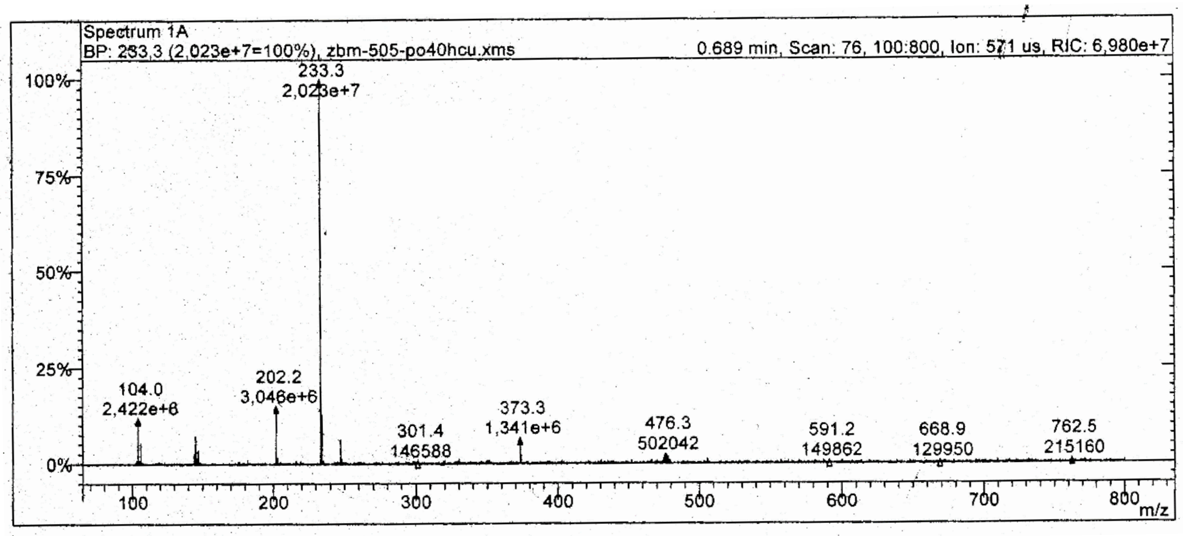

b)

Figure S1: ESIMS spectrum of the **6d** and $CuCl_2$ mixture.

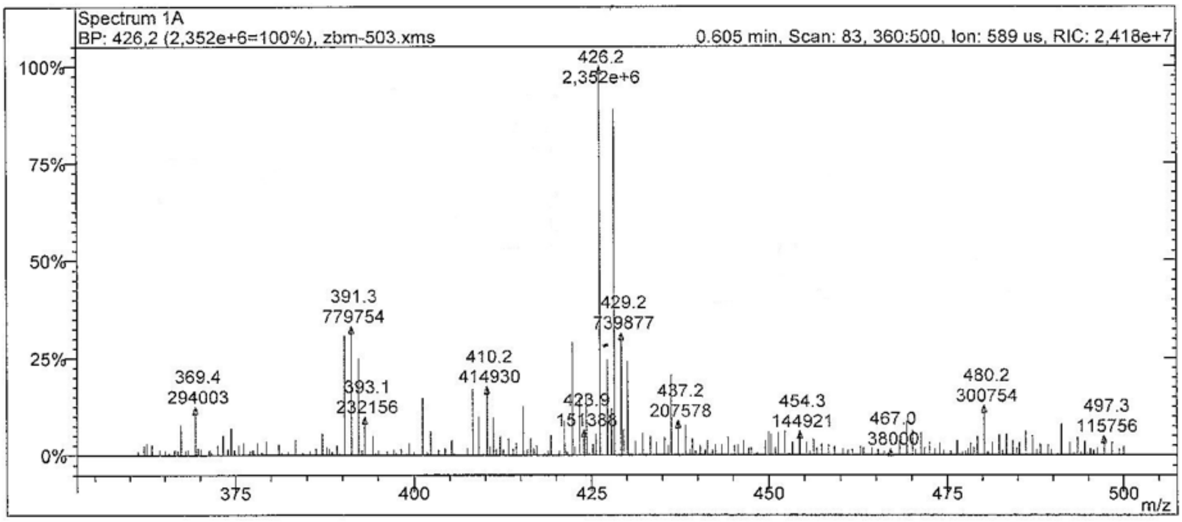

a)

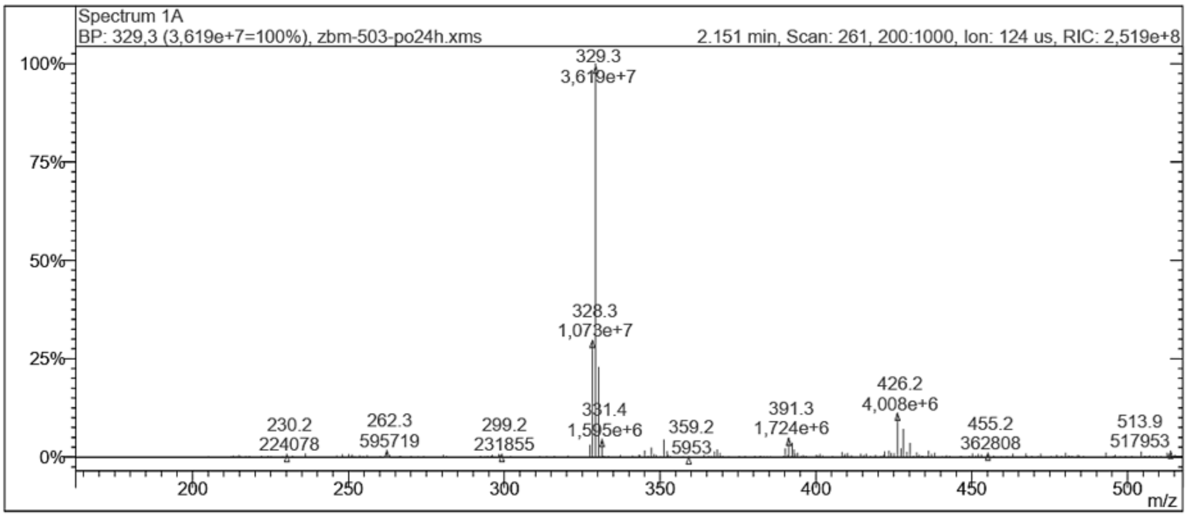

b)

Figure S2: ESIMS spectra of the **5i** and $CuCl_2$ mixture:
a) Spectrum recorded immediately after mixing the solutions of **5i** and $CuCl_2$;
b) Spectrum recorded after 24 h.

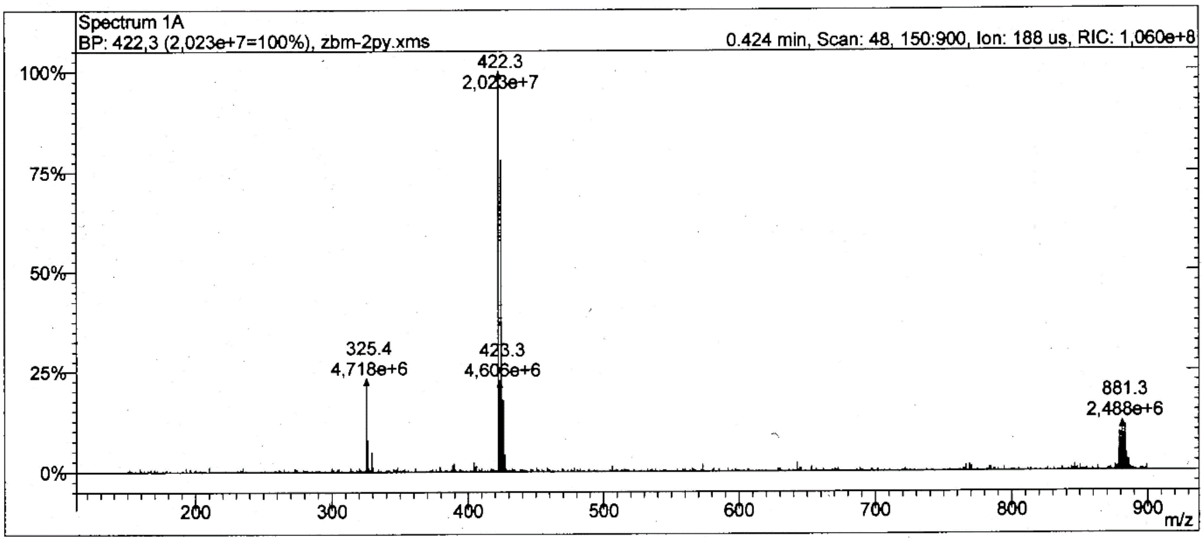

a)

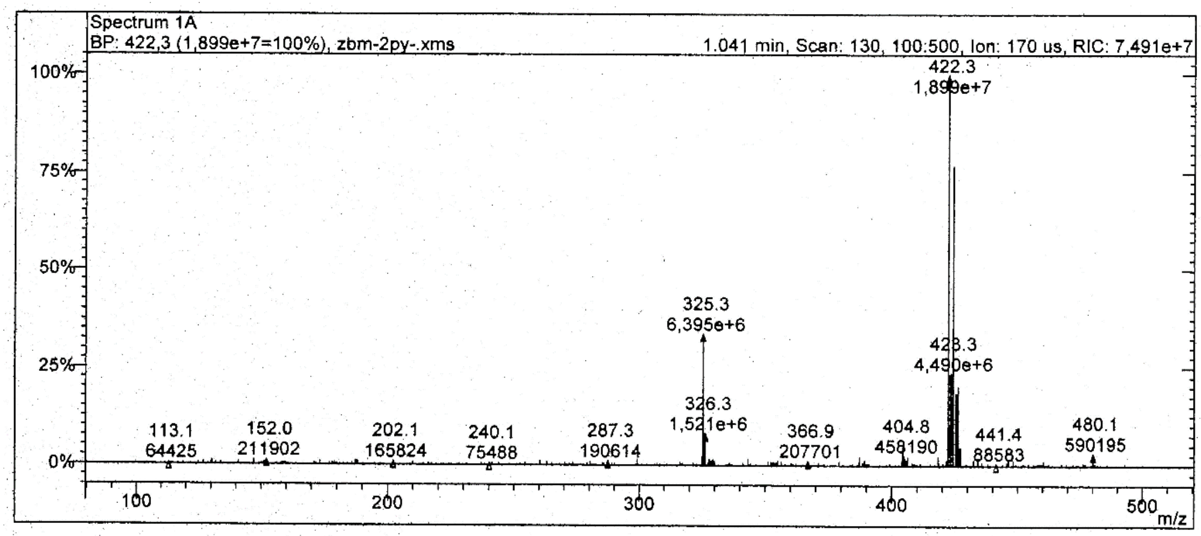

b)

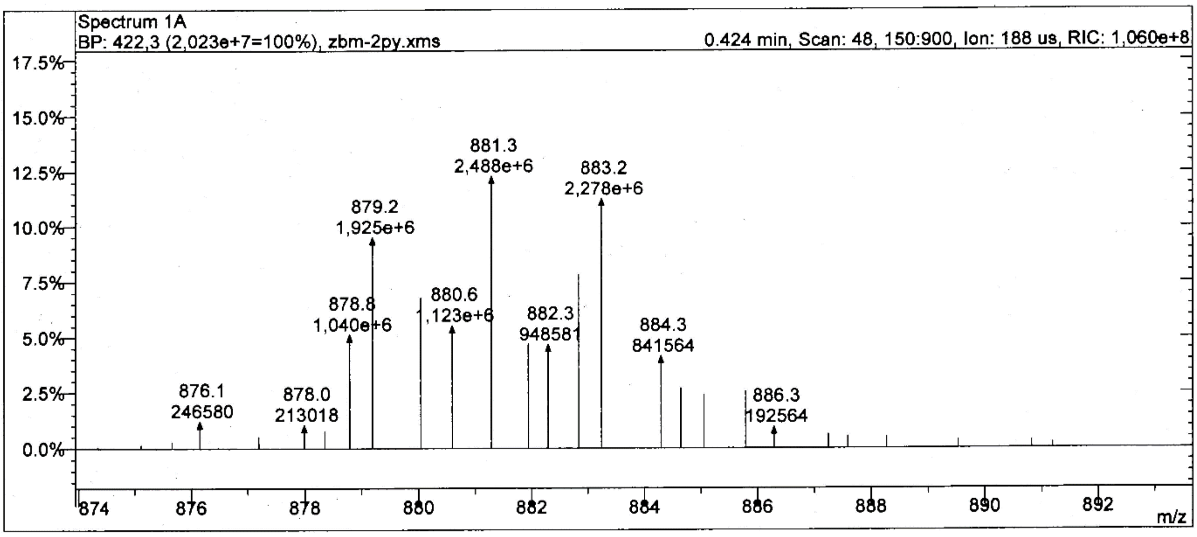

c)

Figure S3: ESIMS spectrum of the **7** and $CuCl_2$ mixture a) and expansions b), c).

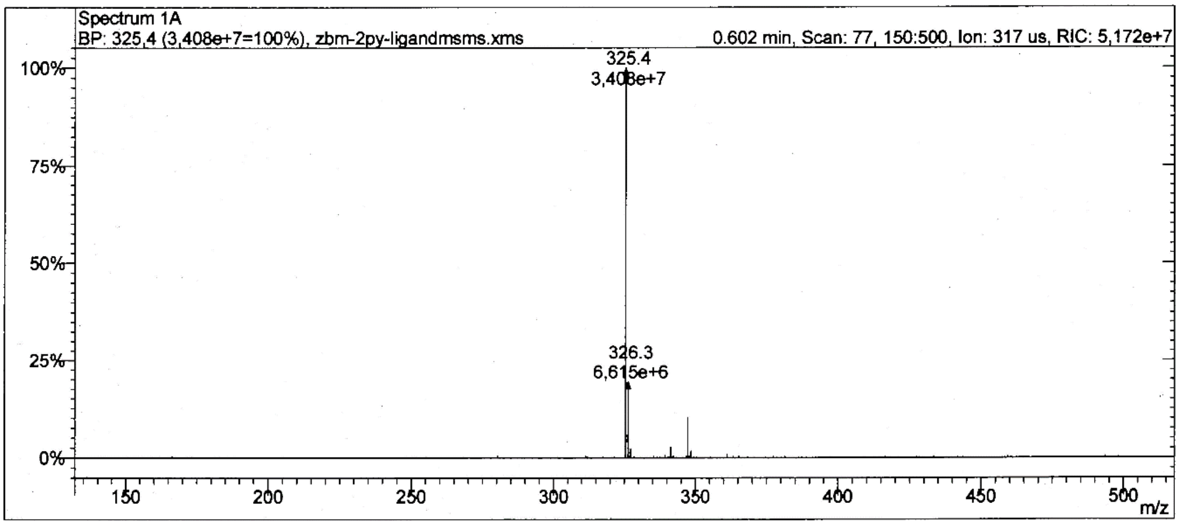

Figure S4: ESIMS spectrum of the **7**.

4. References

1. Malinowski, Z.; Fornal, E.; Sierocińska, B.; Czeczko, R.; Nowak, M. *Tetrahedron* **2016**, *72*, 7942–7951. doi: 10.1016/j.tet.2016.10.022.

2. Outerbridge, V. M.; Landge, S. M.; Tamaki, H.; Török, B. *Synthesis* **2009**, *11*, 1801–1806. doi: 10.1055/s-0028-1088074.

3. Krishnananthan, S.; Smith, D.; Wu, D.-R.; Yip, S.; Gunaga, Pr.; Mathur, A.; Li, J. *J. Org. Chem.* **2016**, *81*, 1520–1526. doi: 10.1021/acs.joc.5b02652.

4. Koermendy, K.; Ruff, F. *Acta Chimica Academiae Scientiarum Hungaricae*, **1981**, *106*, 155–166.

5. Mosmann T. *J. Immunol. Methods* **1983**, *65*, 55–63.
doi: https://doi.org/10.1016/0022-1759(83)90303-4.

6. Stockert, J. C.; Horobin, R. W.; Colombo, L. L.; Blázquez-Castro, A. *Acta Histochem.* **2018**, *120*,159–167. doi: https://doi.org/10.1016/j.acthis.2018.02.005.

7. Da Violante, G.; Zerrouk, N.; Richard, I.; Provot, G.; Chaumeil, J. C.; Arnaud, P. *Biol. Pharm. Bull.* **2002**, *25*,1600-1603.
doi: https://doi.org/10.1248/bpb.25.1600.

5. ^{1}H, ^{13}C NMR, and FTIR spectra of compounds **3**, **5**, **6**

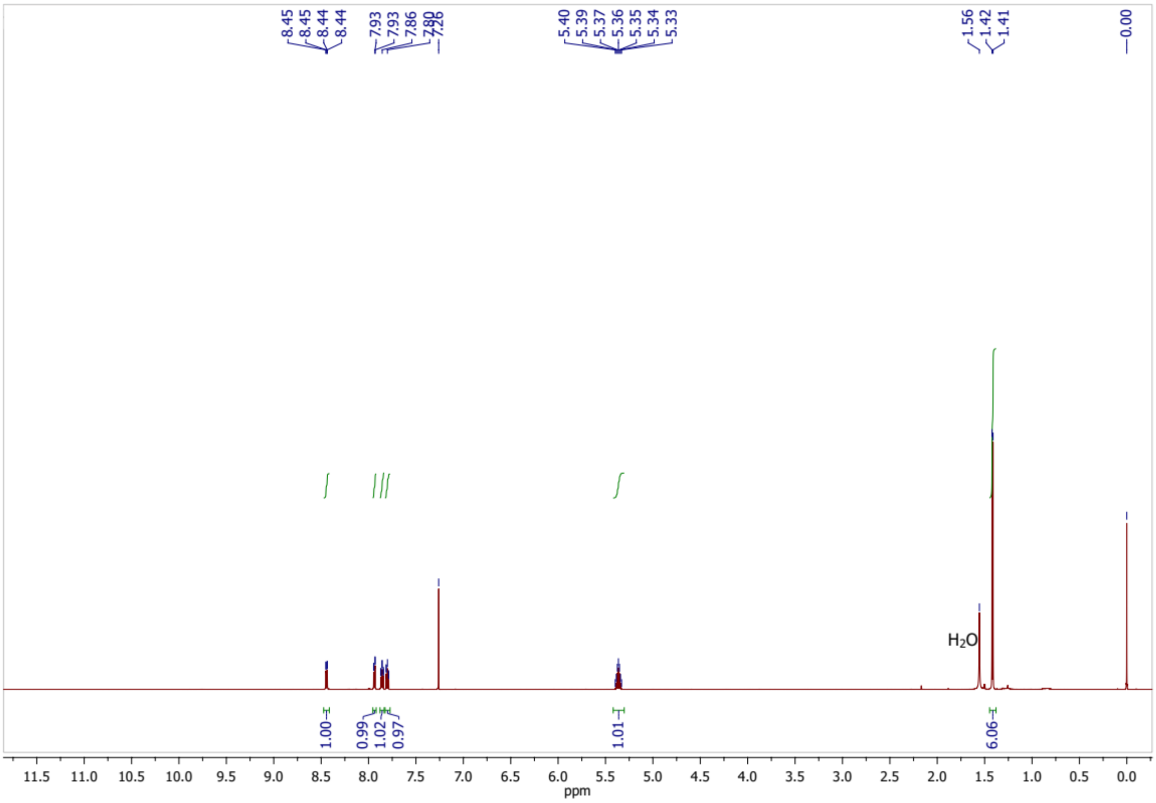

Figure S5: ^{1}H NMR spectrum of **3b**.

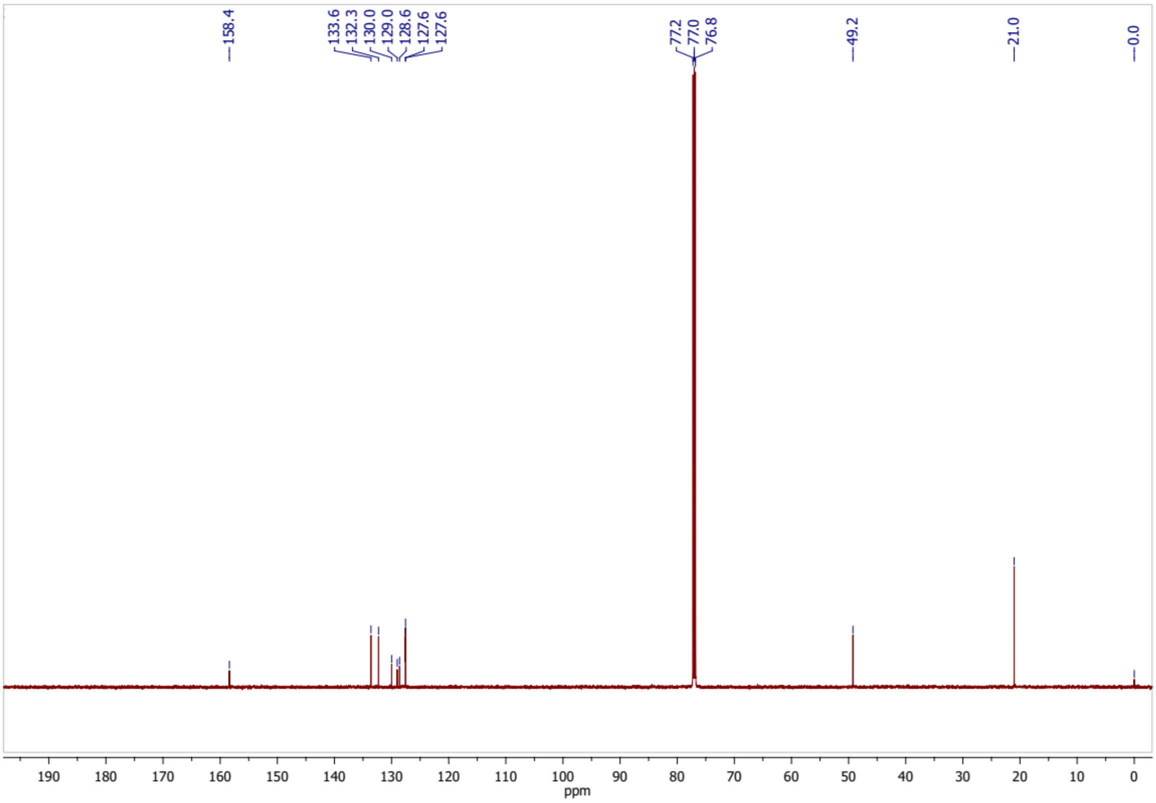

Figure S6: ^{13}C NMR spectrum of **3b**.

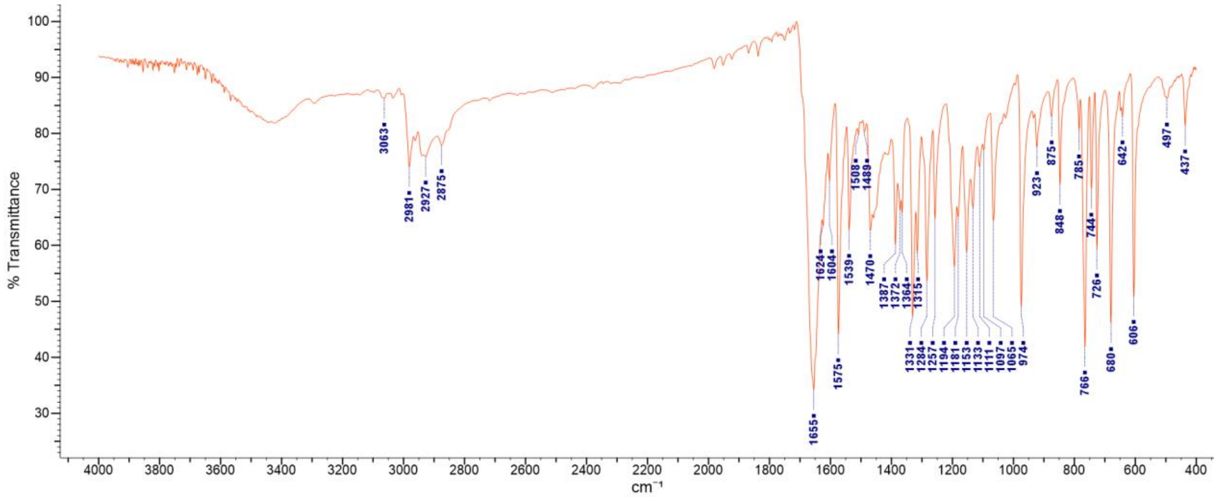

Figure S7: FTIR spectrum of **3b**.

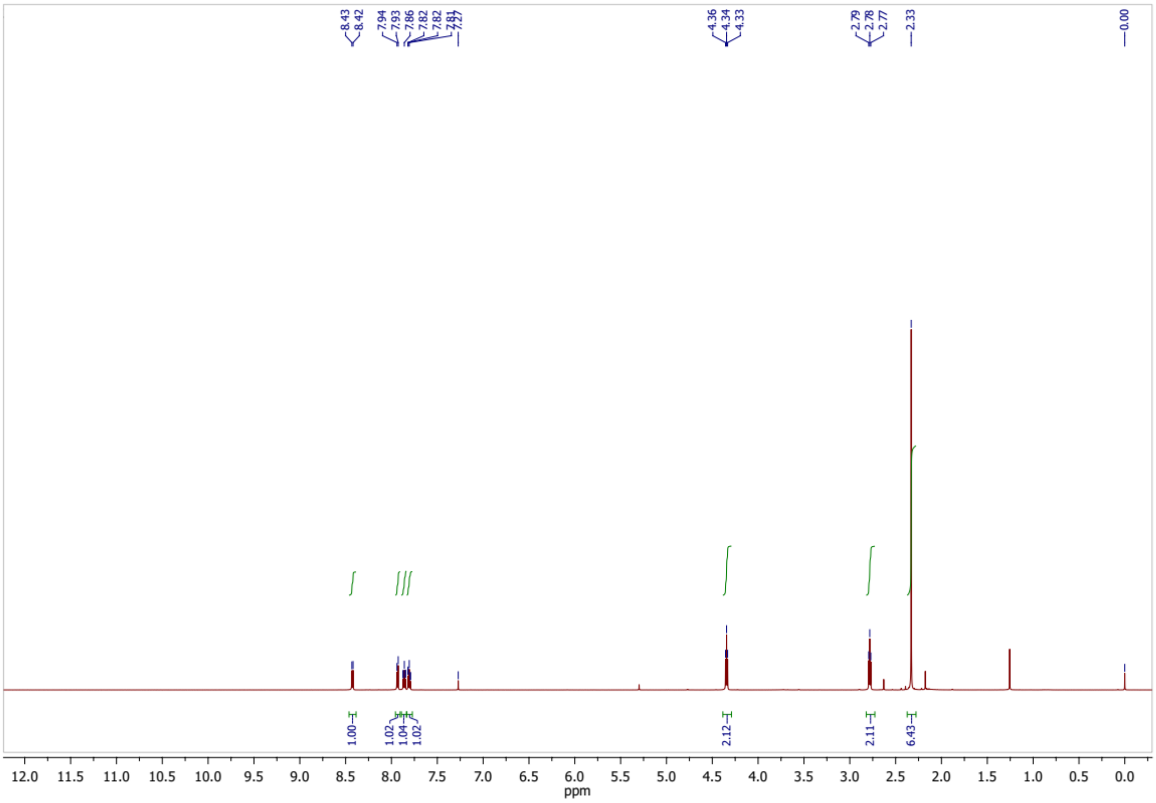

Figure S8: ^{1}H NMR spectrum of **3c**.

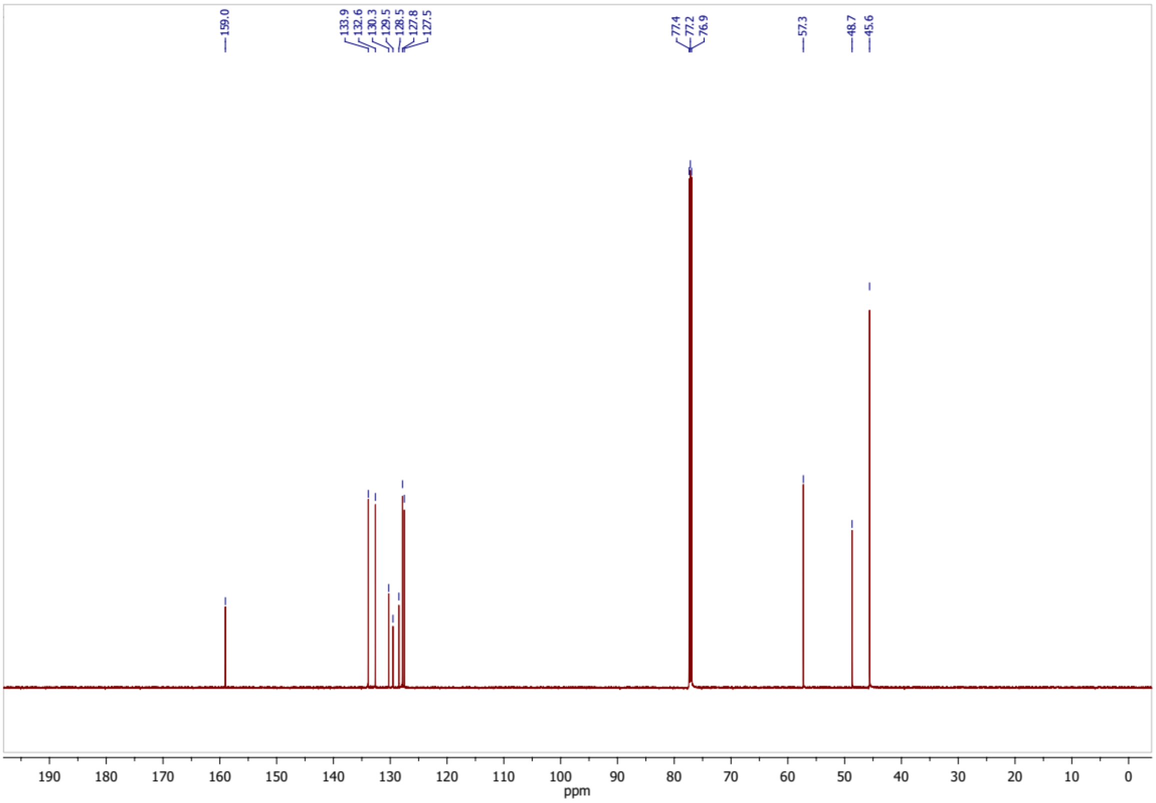

Figure S9: ^{13}C NMR spectrum of **3c**.

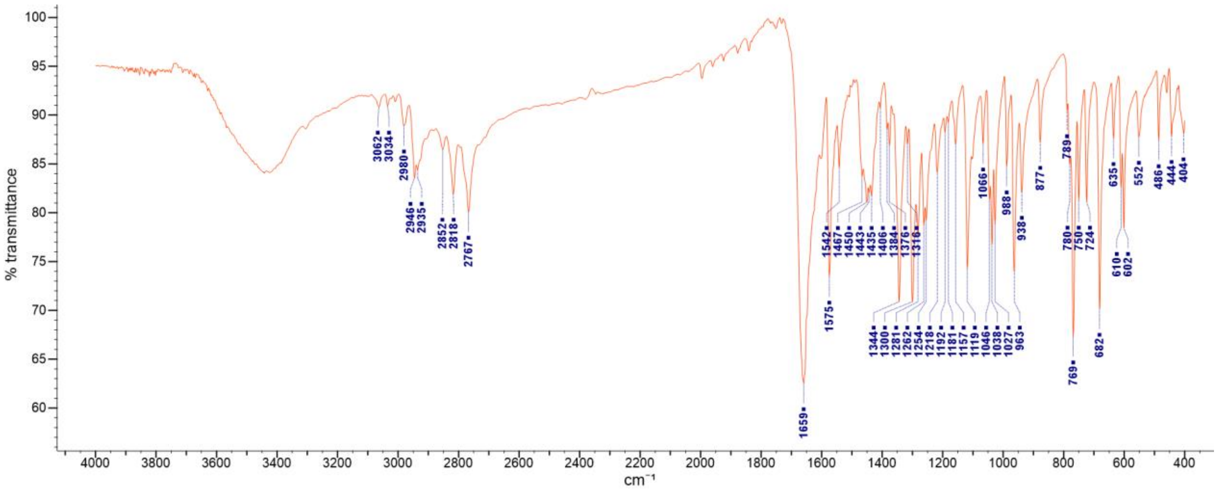

Figure S10: FTIR spectrum of **3c**.

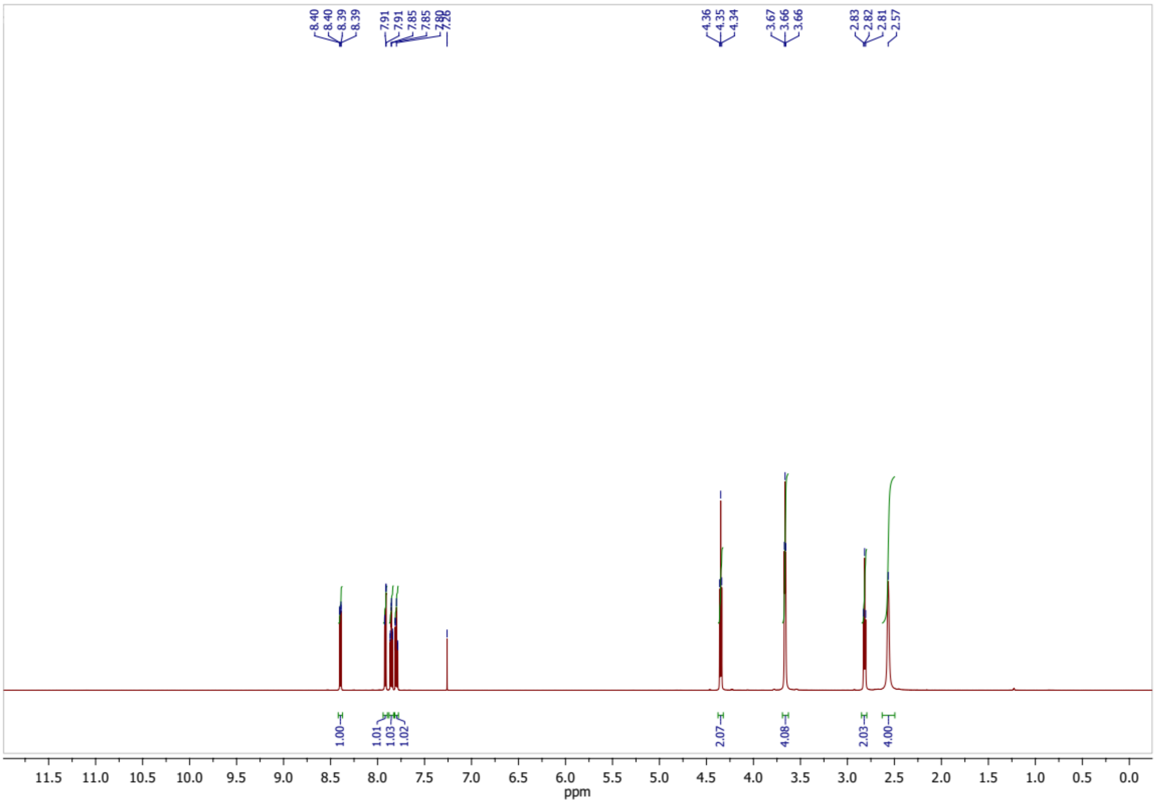

Figure S11: ^{1}H NMR spectrum of **3d**.

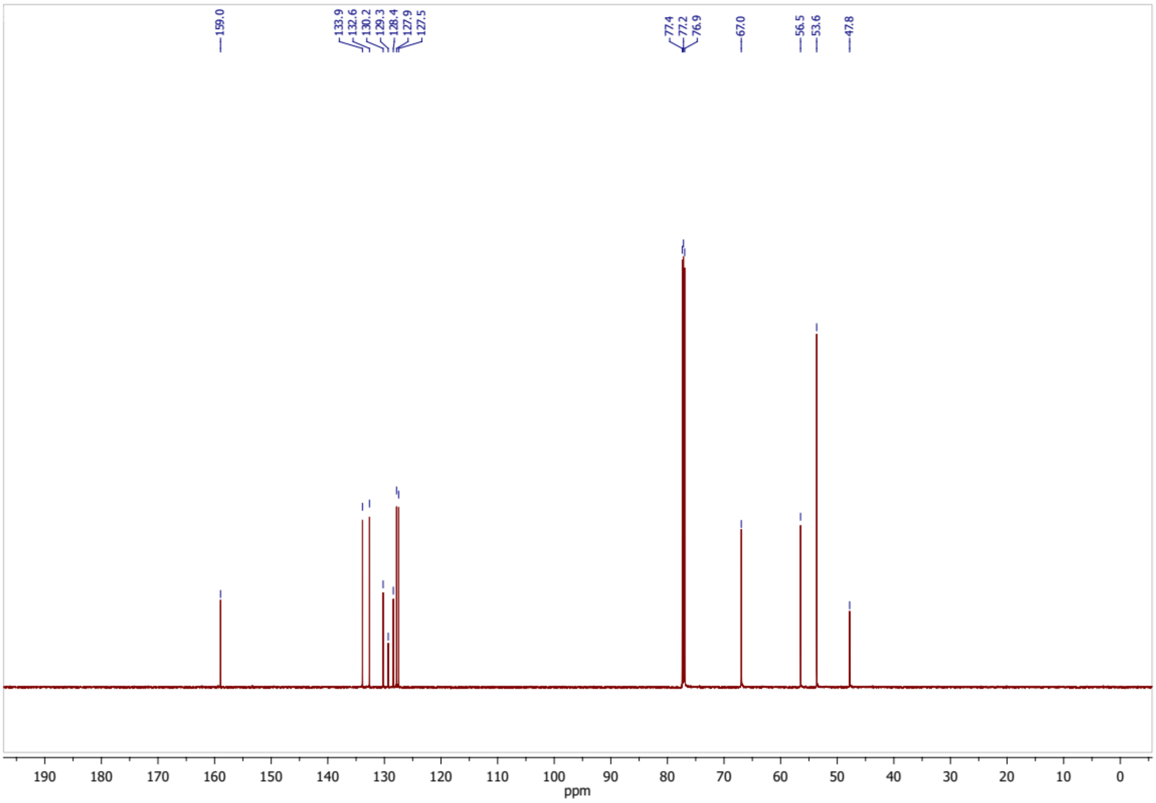

Figure S12: ^{13}C NMR spectrum of **3d**.

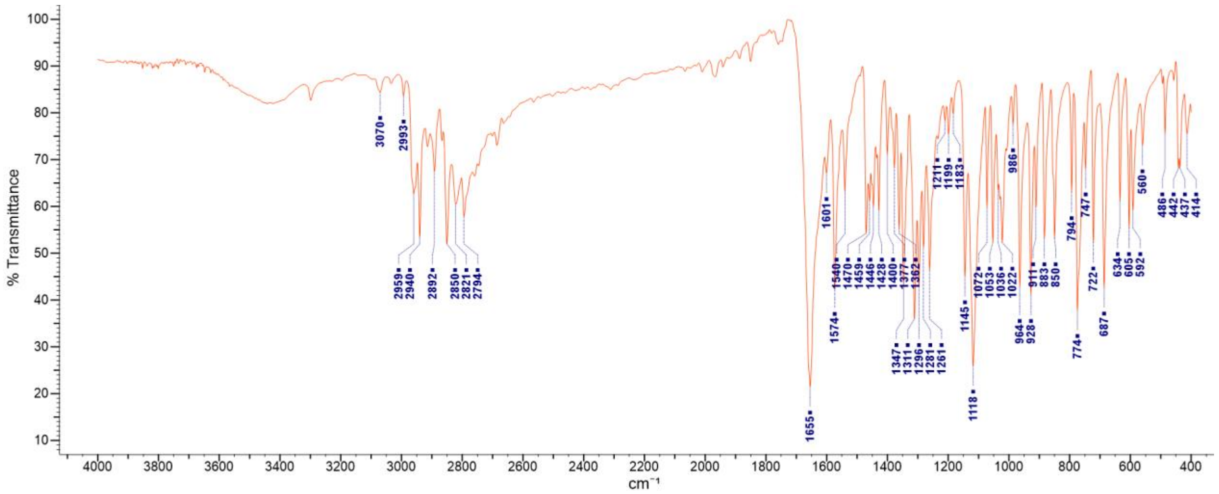

Figure S13: FTIR spectrum of **3d**.

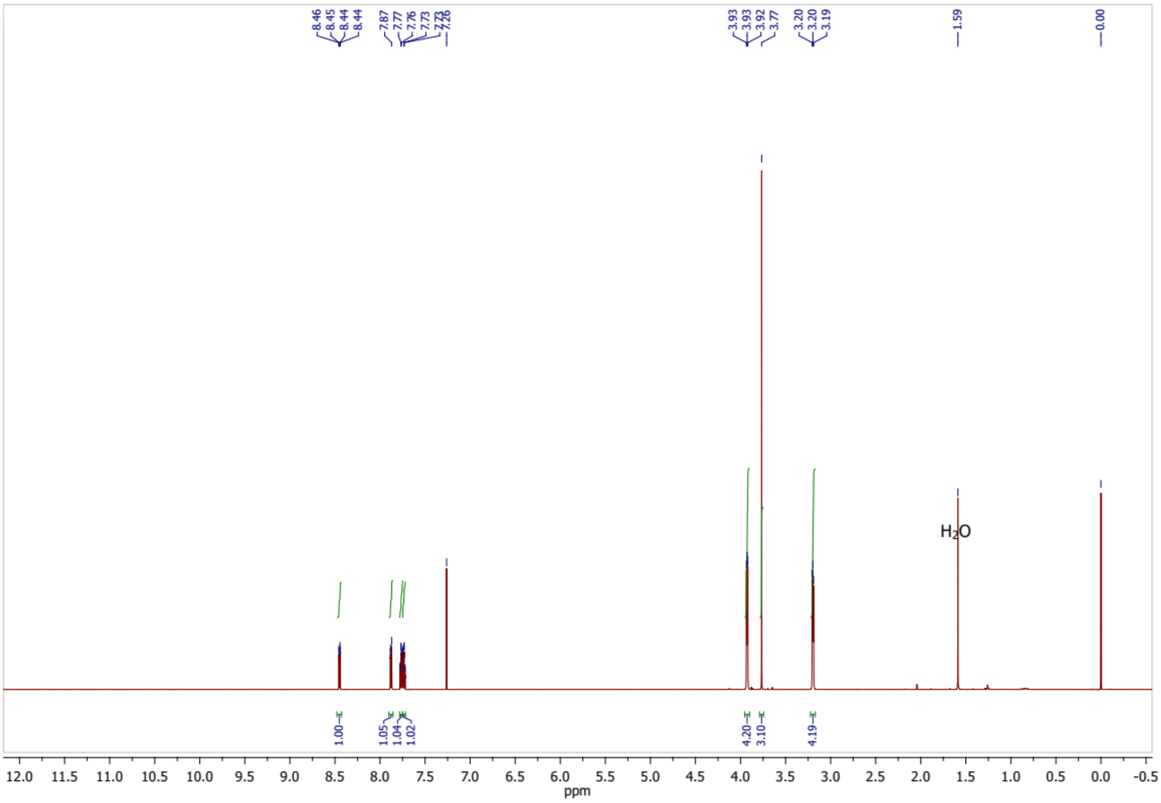

Figure S14: ^{1}H NMR spectrum of **5a**.

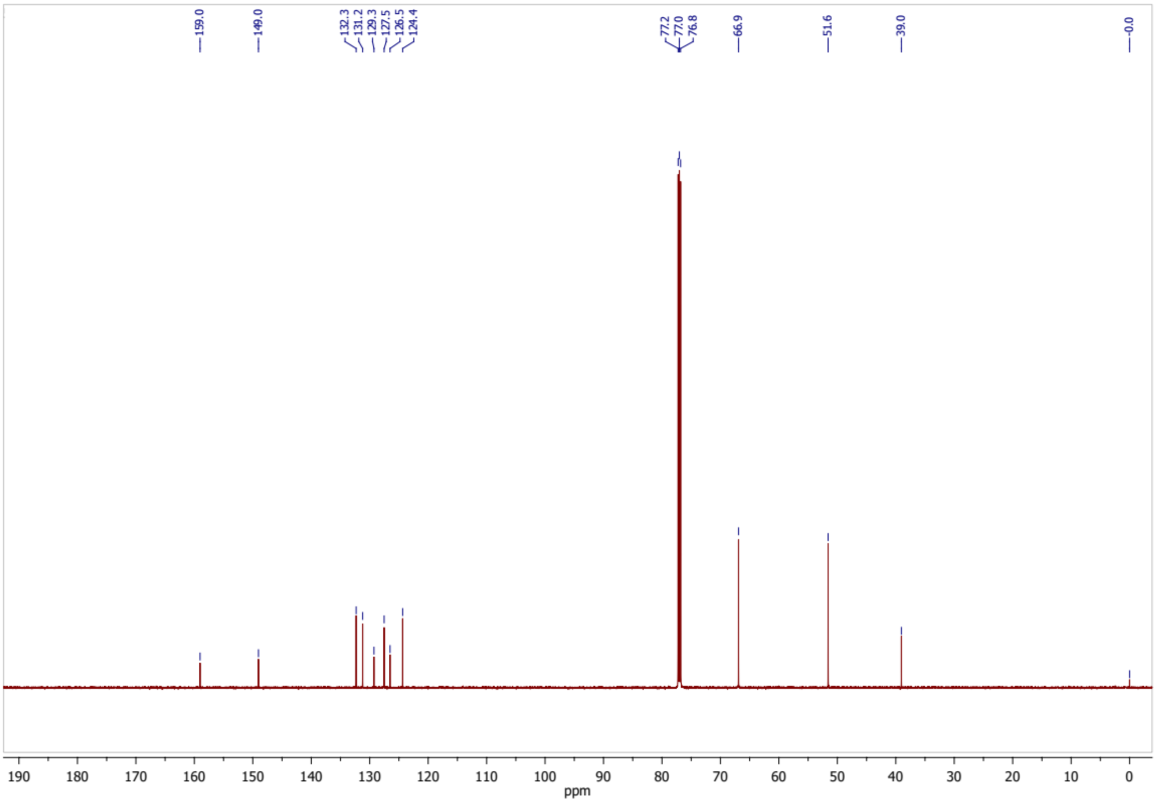

Figure S15: ^{13}C NMR spectrum of **5a**.

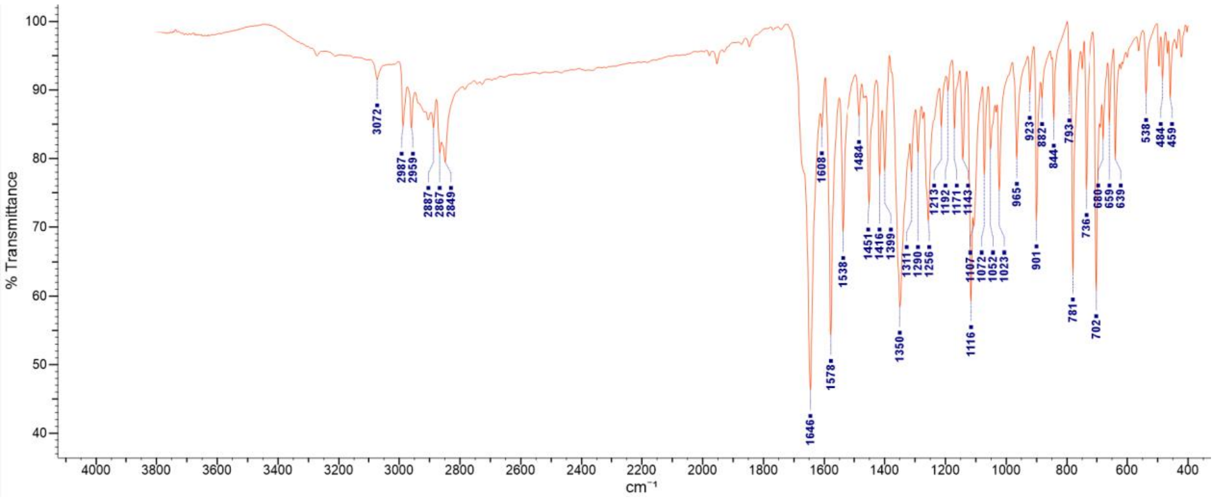

Figure S16: FTIR spectrum of **5a**.

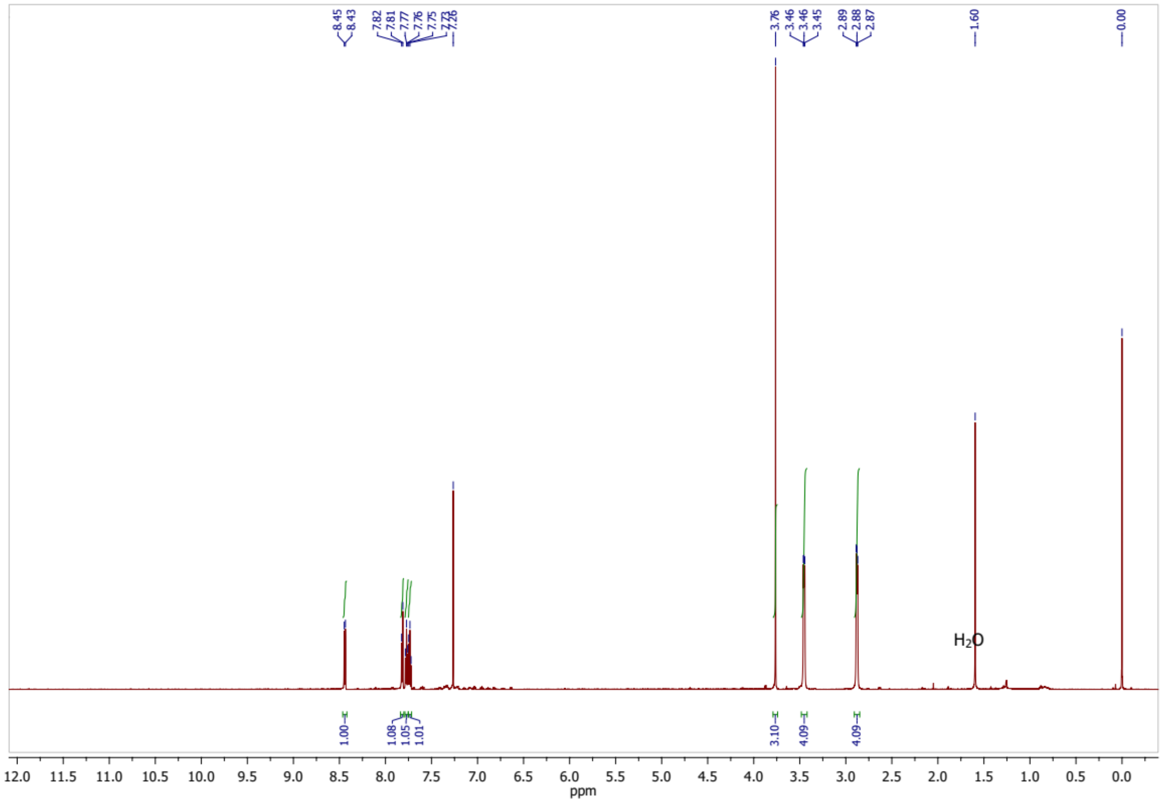

Figure S17: ^{1}H NMR spectrum of **5b**.

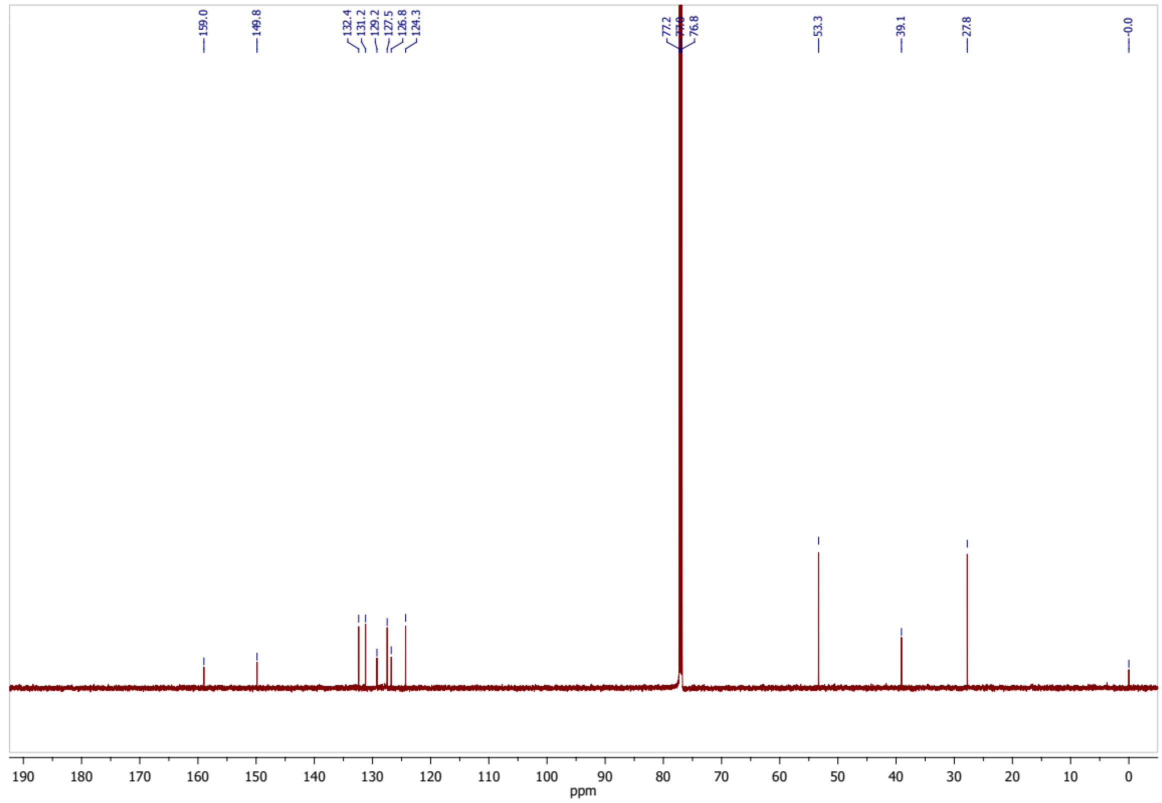

Figure S18: ^{13}C NMR spectrum of **5b**.

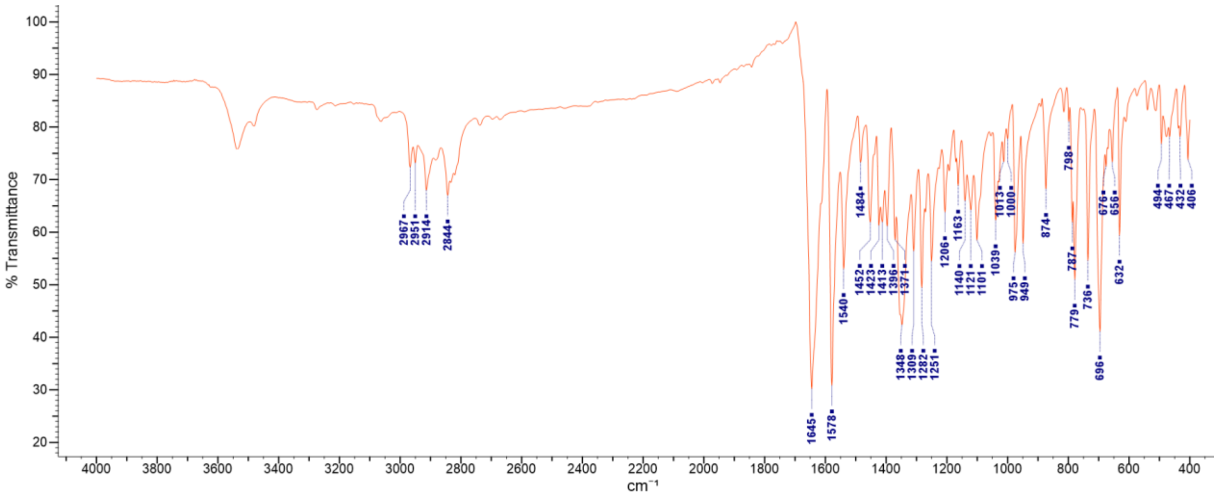

Figure S19: FTIR spectrum of **5b**.

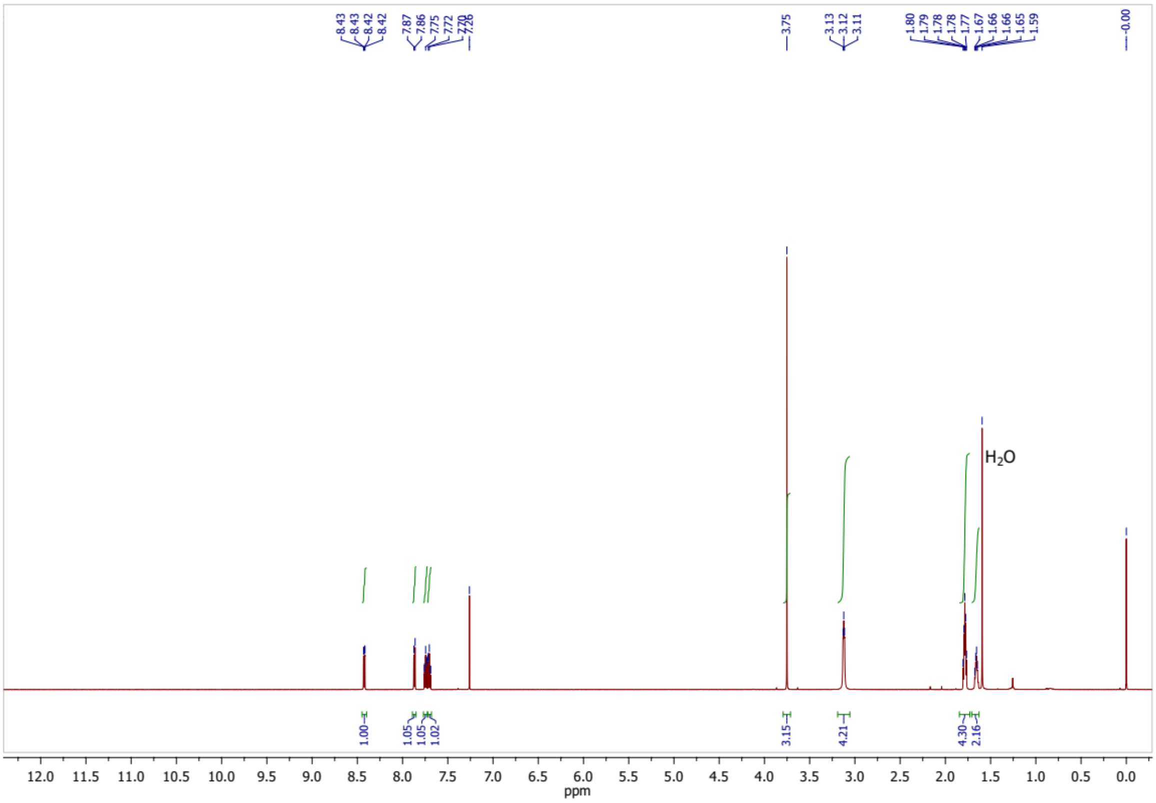

Figure S20: ^{1}H NMR spectrum of **5c**.

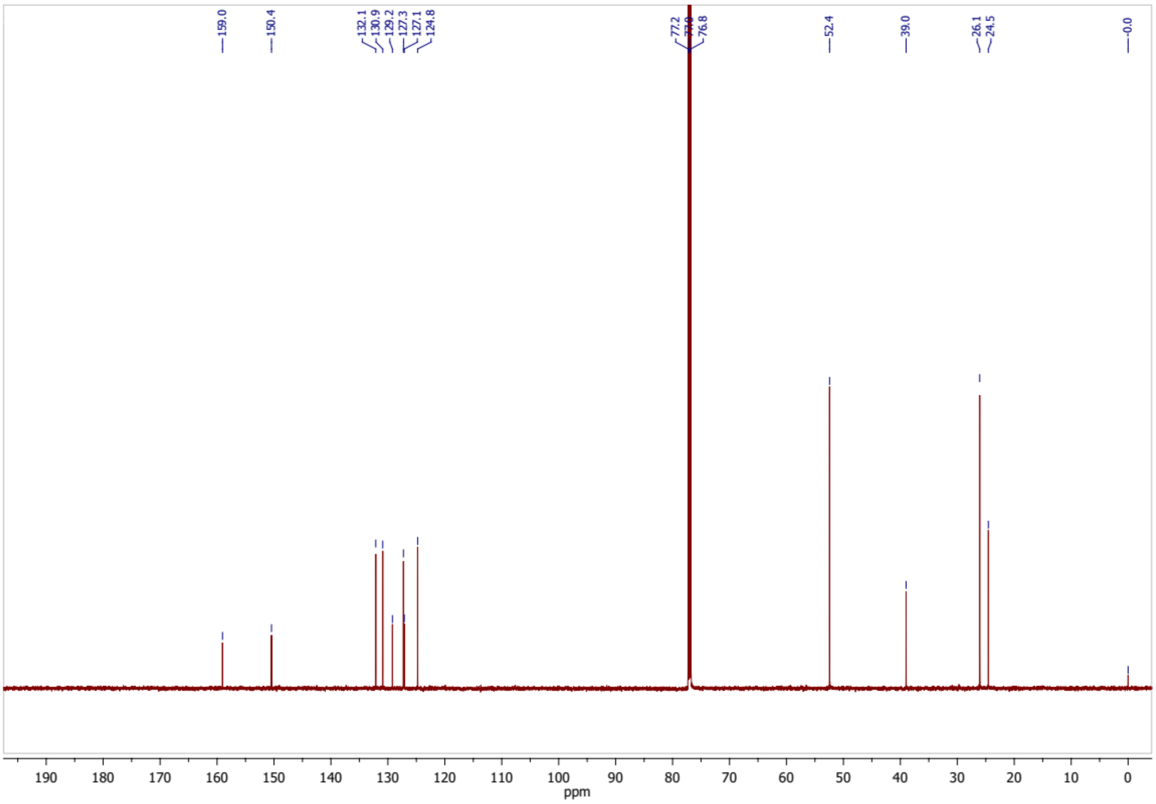

Figure S21: ^{13}C NMR spectrum of **5c**.

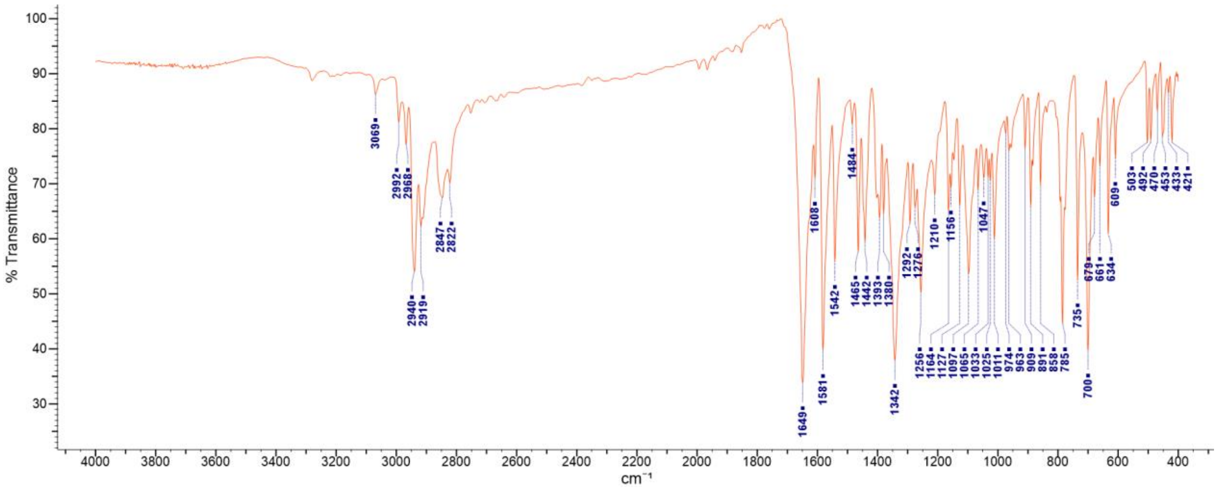

Figure S22: FTIR spectrum of **5c**.

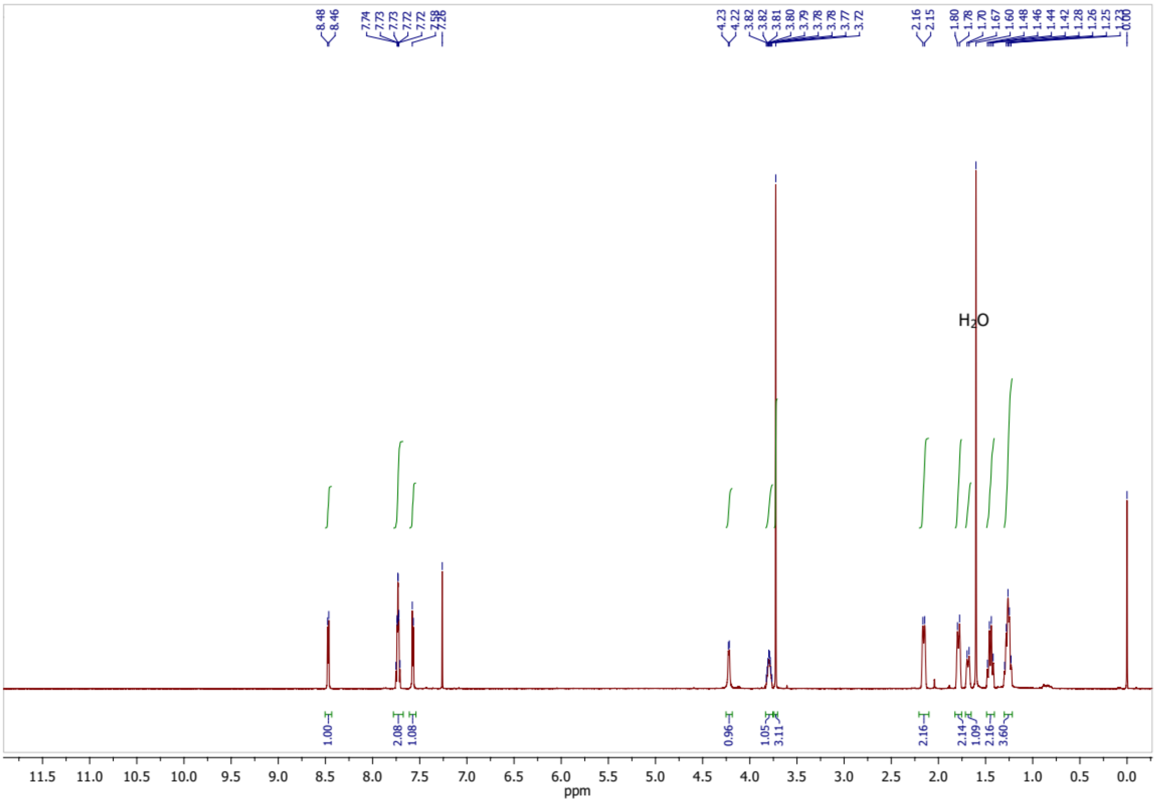

Figure S23: ^{1}H NMR spectrum of **5d**.

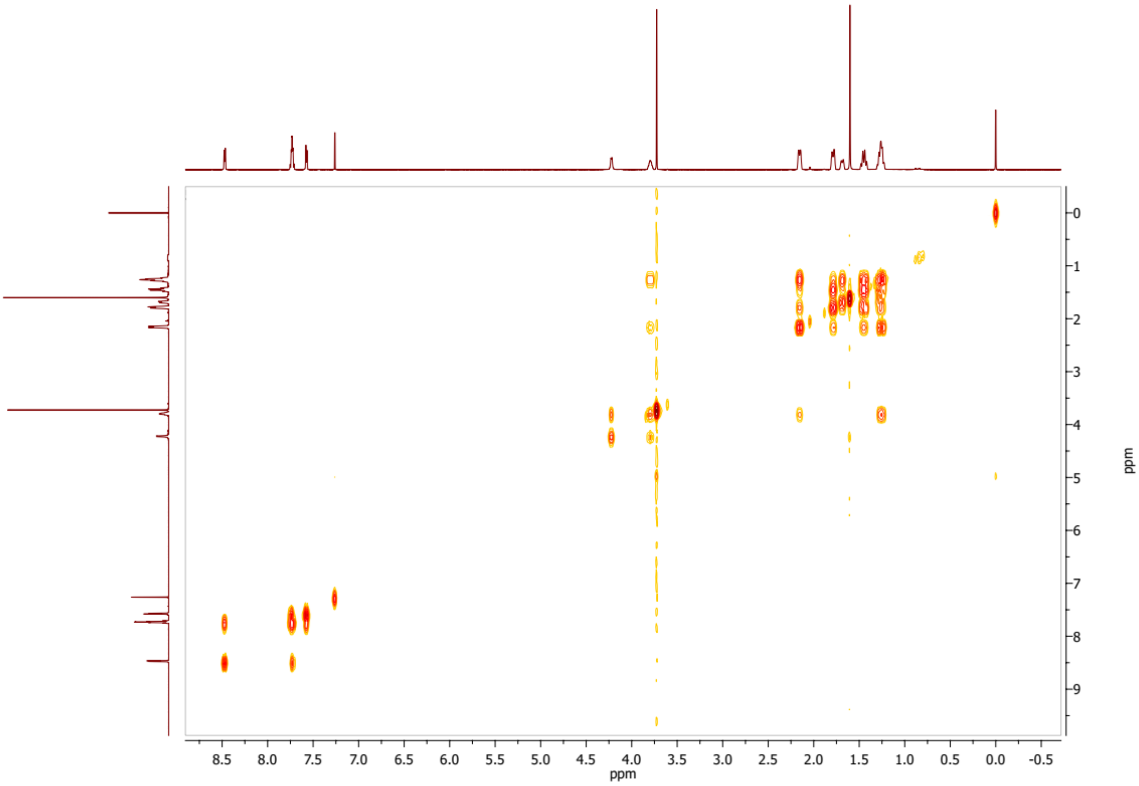

Figure S24: ^{1}H, ^{1}H COSY NMR spectrum of **5d**.

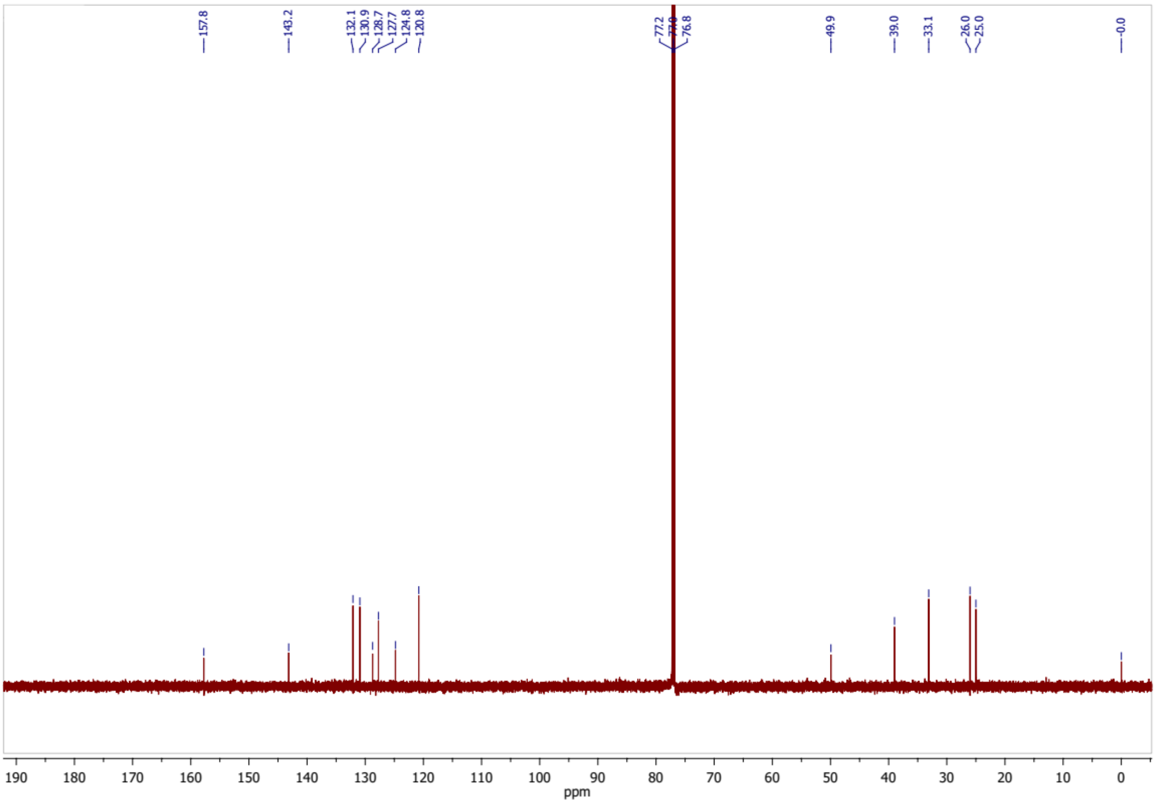

Figure S25: ^{13}C NMR spectrum of **5d**.

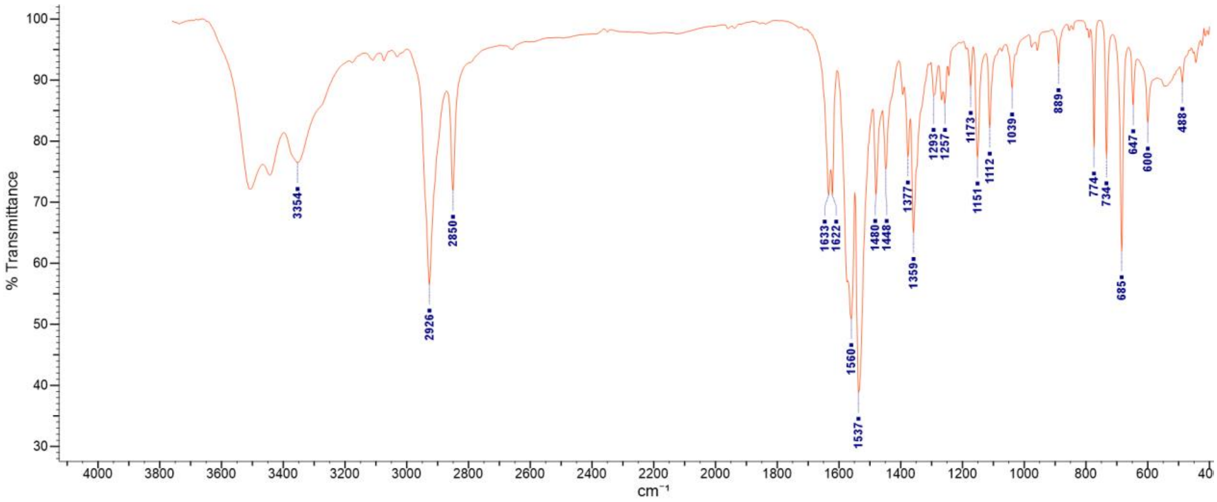

Figure S26: FTIR spectrum of **5d**.

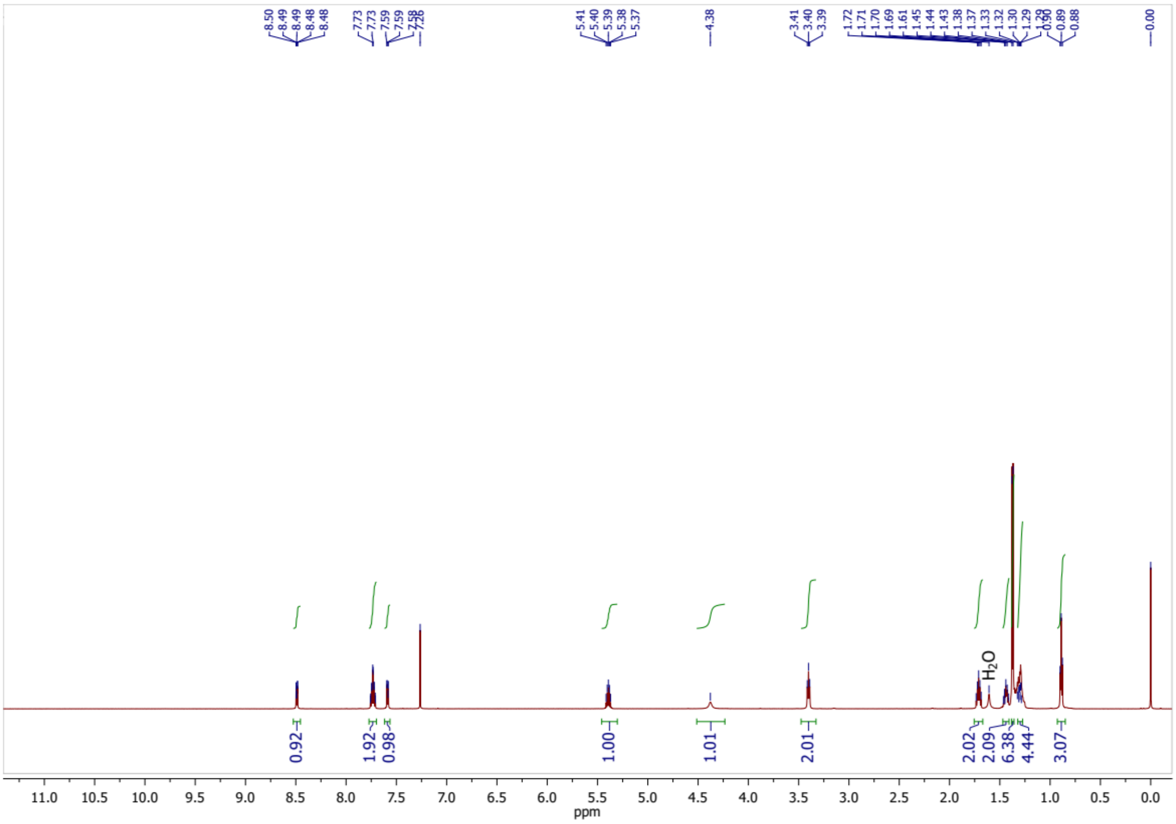

Figure S27: ^{1}H NMR spectrum of **5e**.

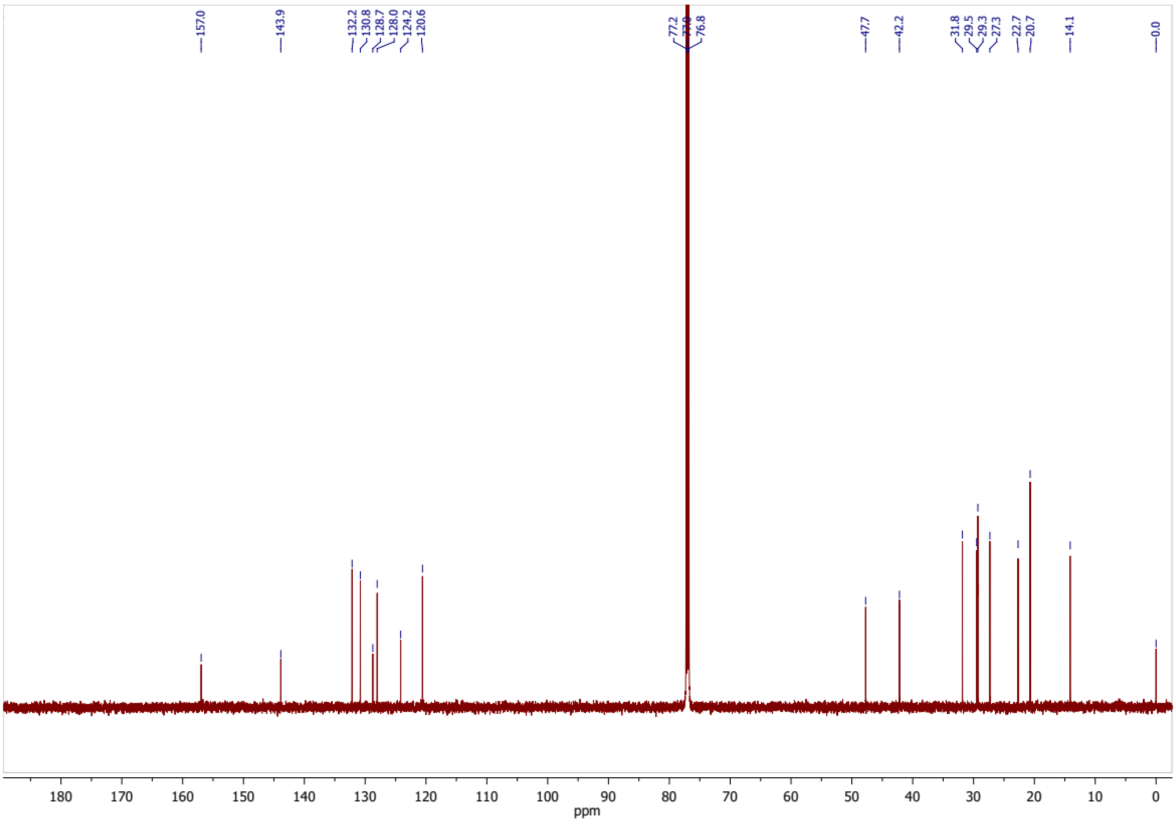

Figure S28: ^{13}C NMR spectrum of **5e**.

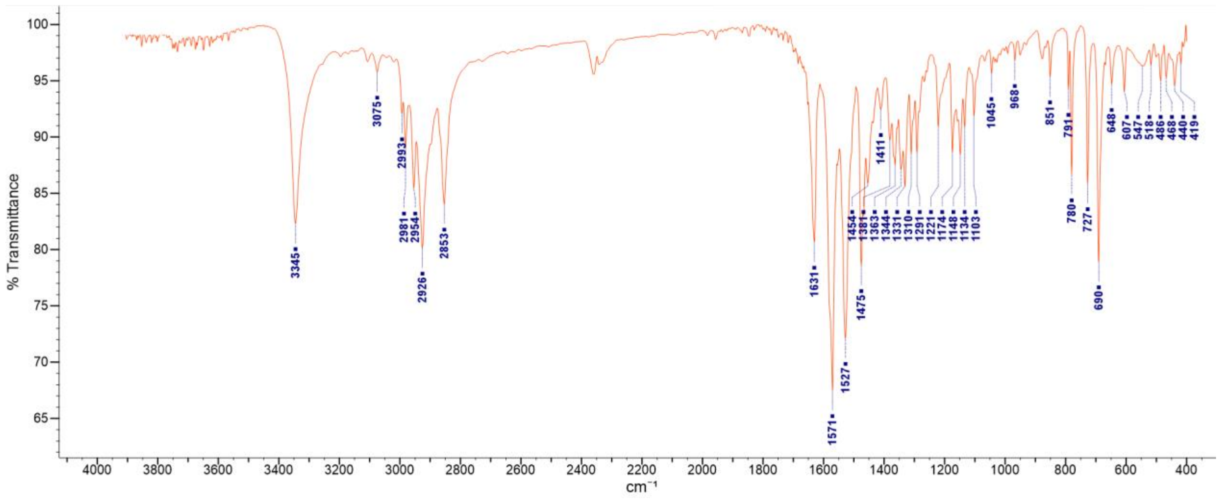

Figure S29: FTIR spectrum of **5e**.

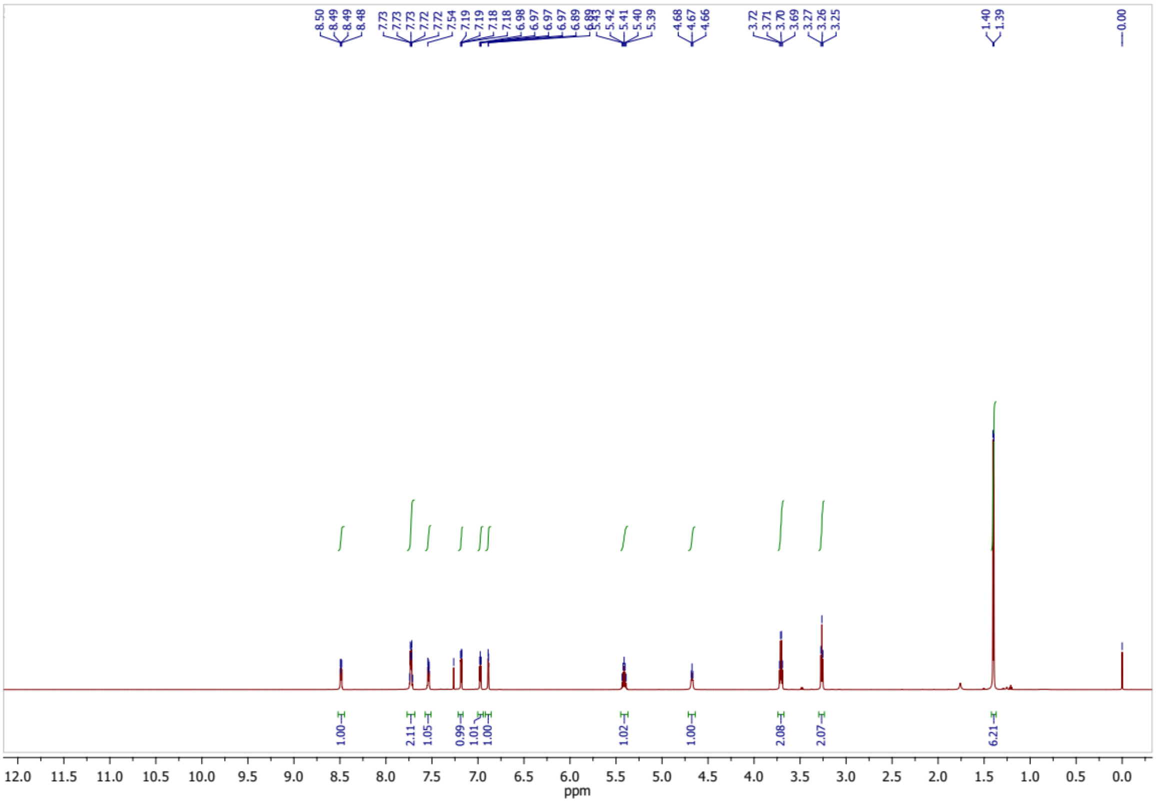

Figure S30: ^{1}H NMR spectrum of **5f**.

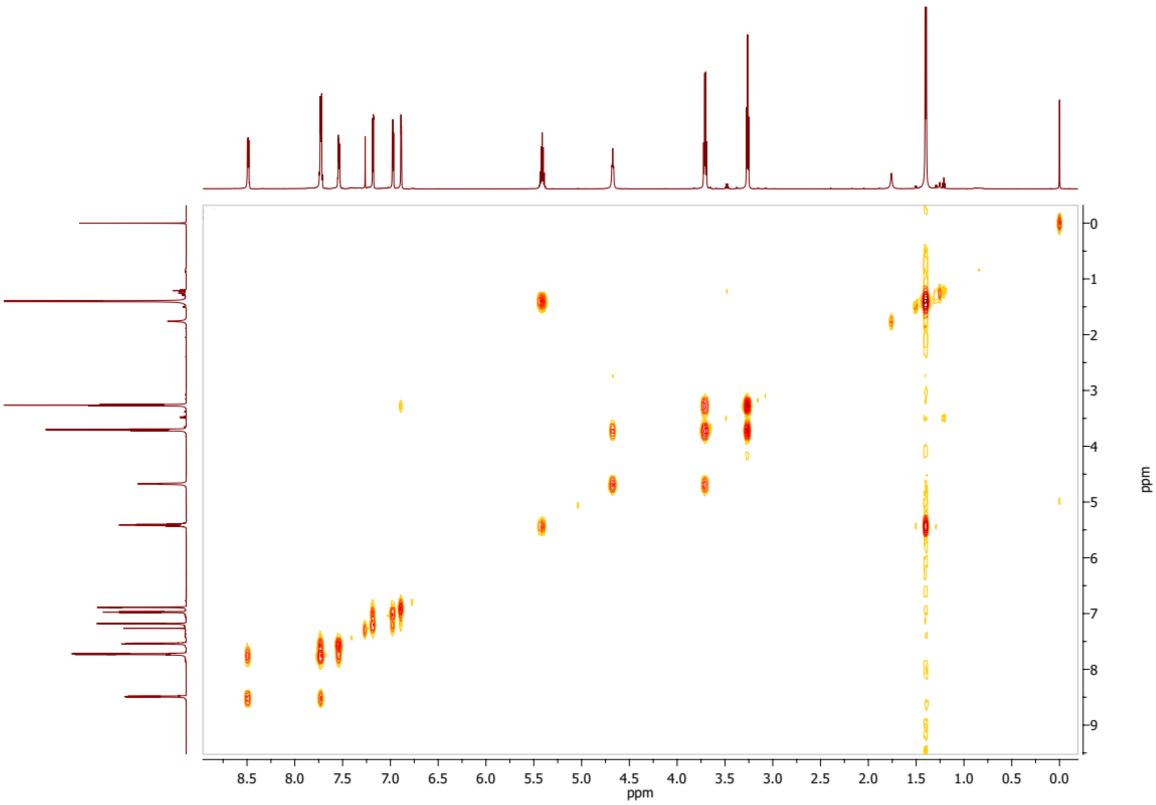

Figure S31: ^{1}H, ^{1}H COSY NMR spectrum of **5f**.

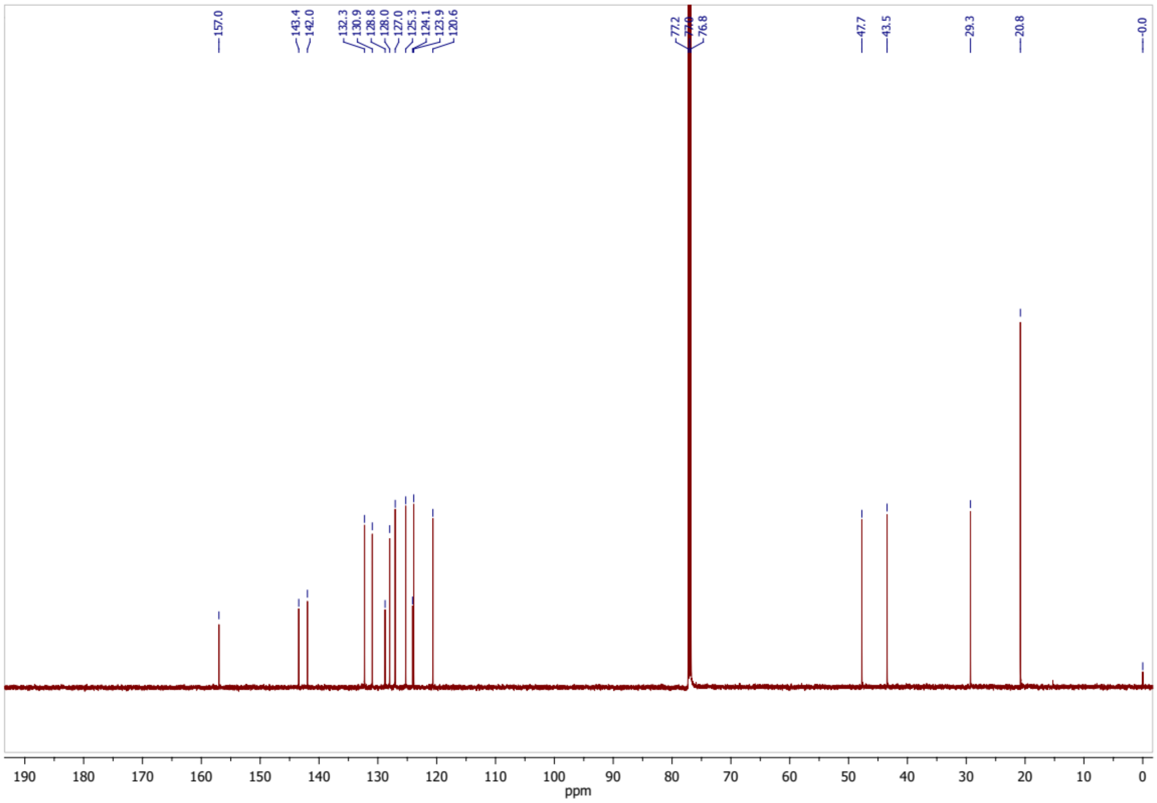

Figure S32: ^{13}C NMR spectrum of **5f**.

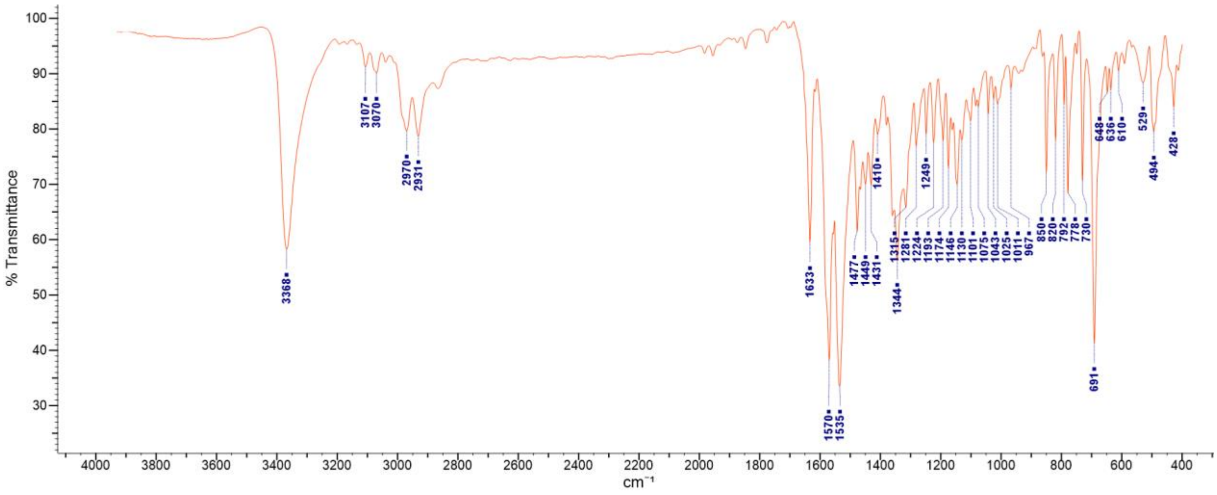

Figure S33: FTIR spectrum of **5f**.

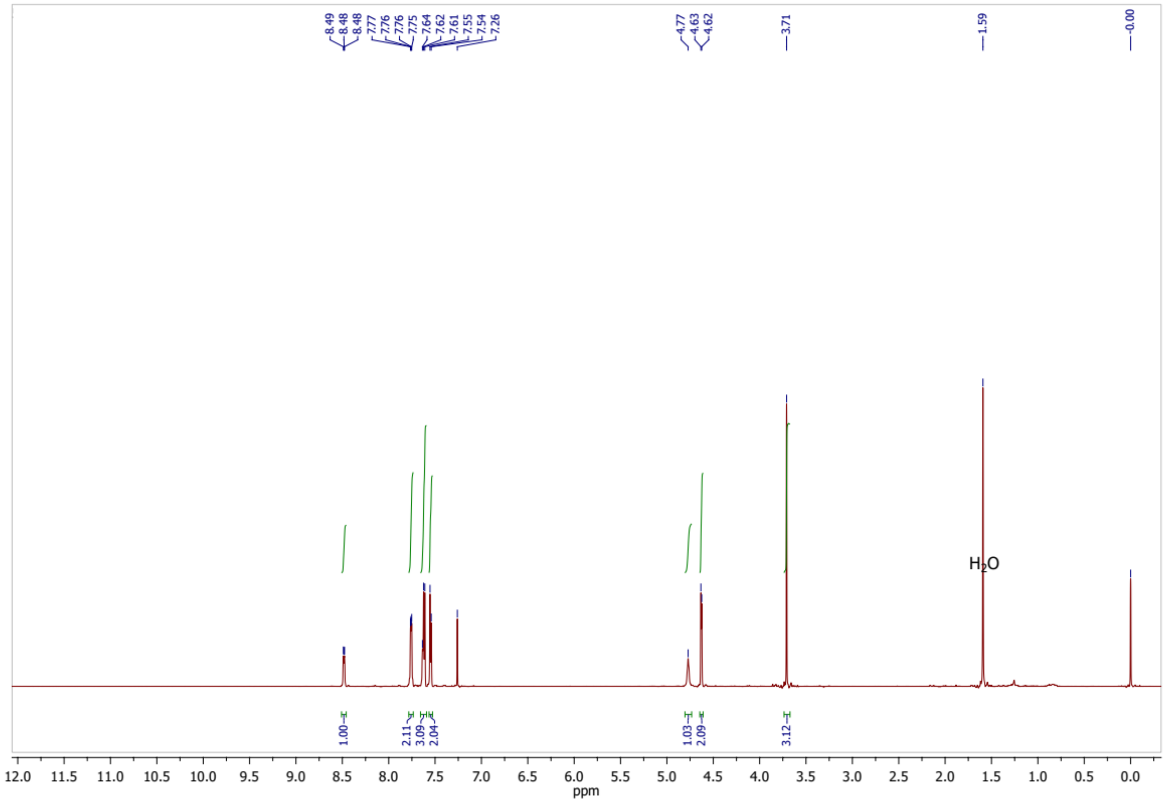

Figure S34: ^{1}H NMR spectrum of **5g**.

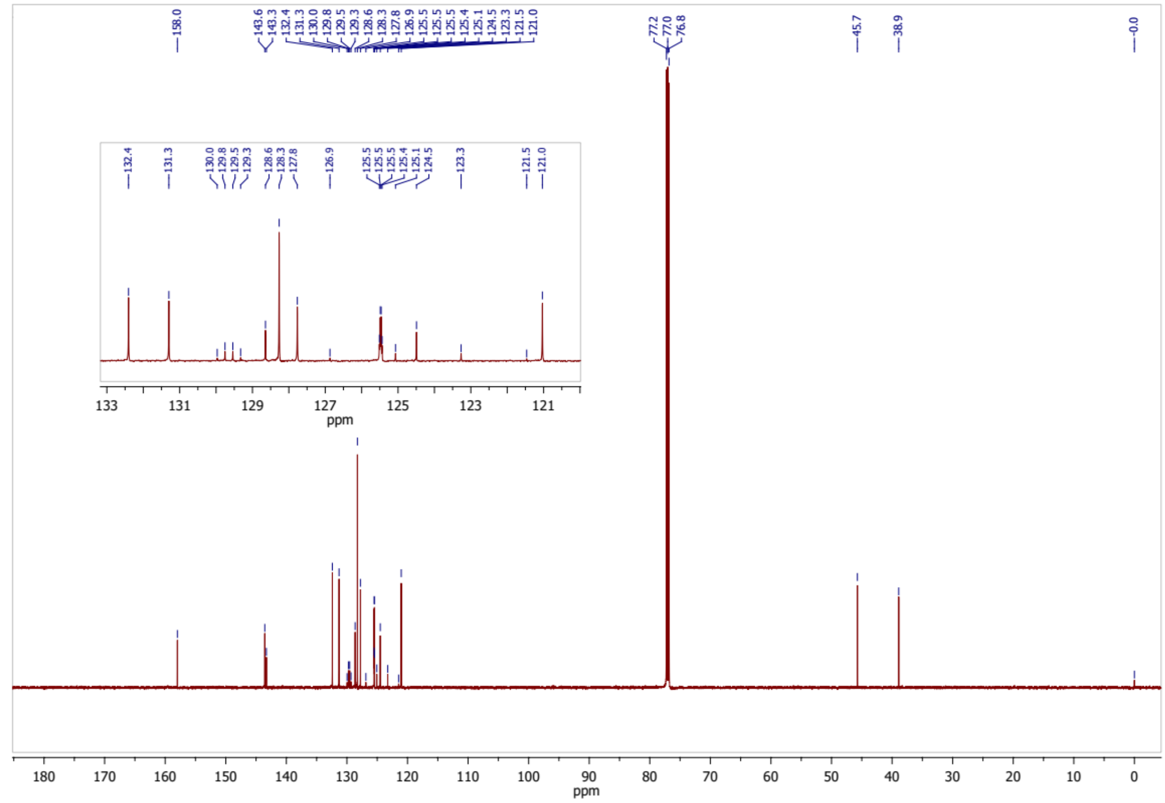

Figure S35: ^{13}C NMR spectrum of **5g**.

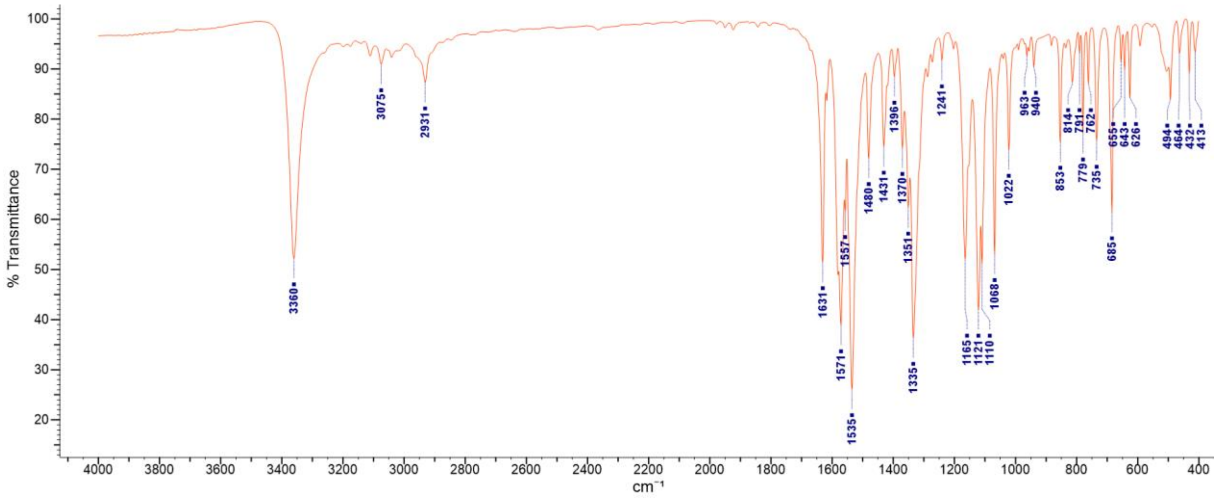

Figure S36: FTIR spectrum of **5g**.

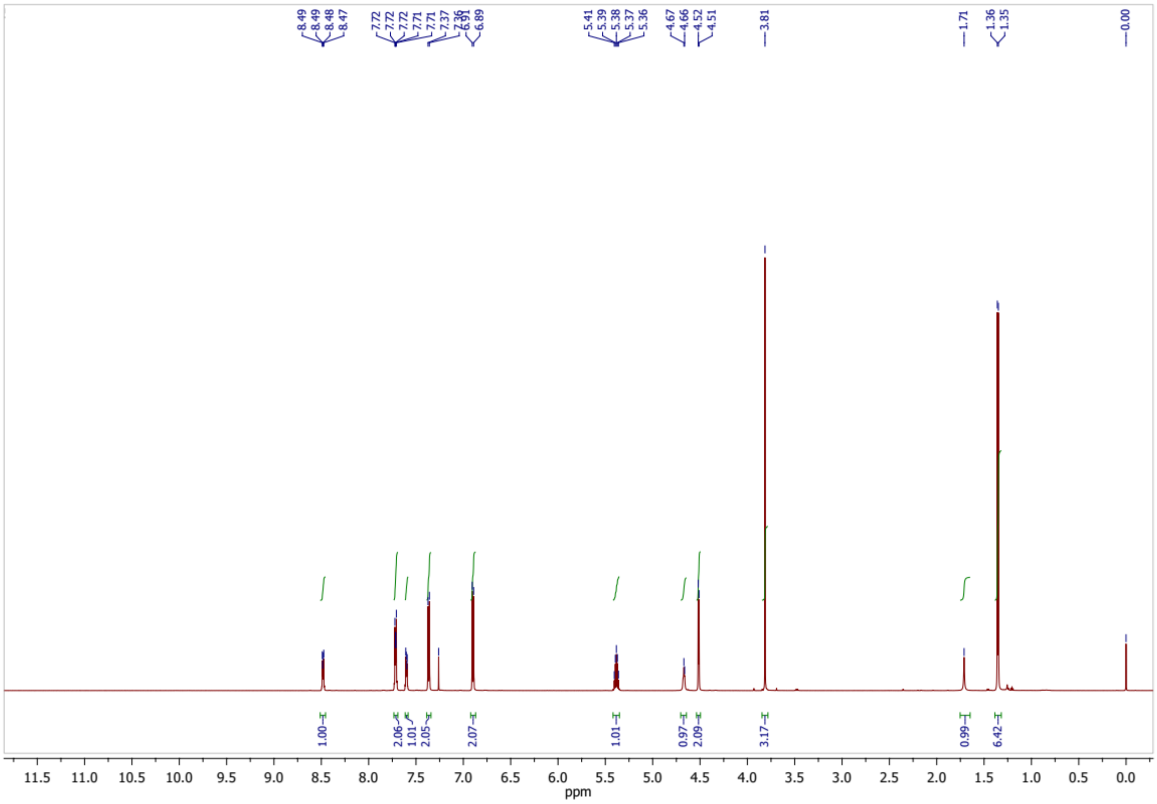

Figure S37: ^{1}H NMR spectrum of **5h**.

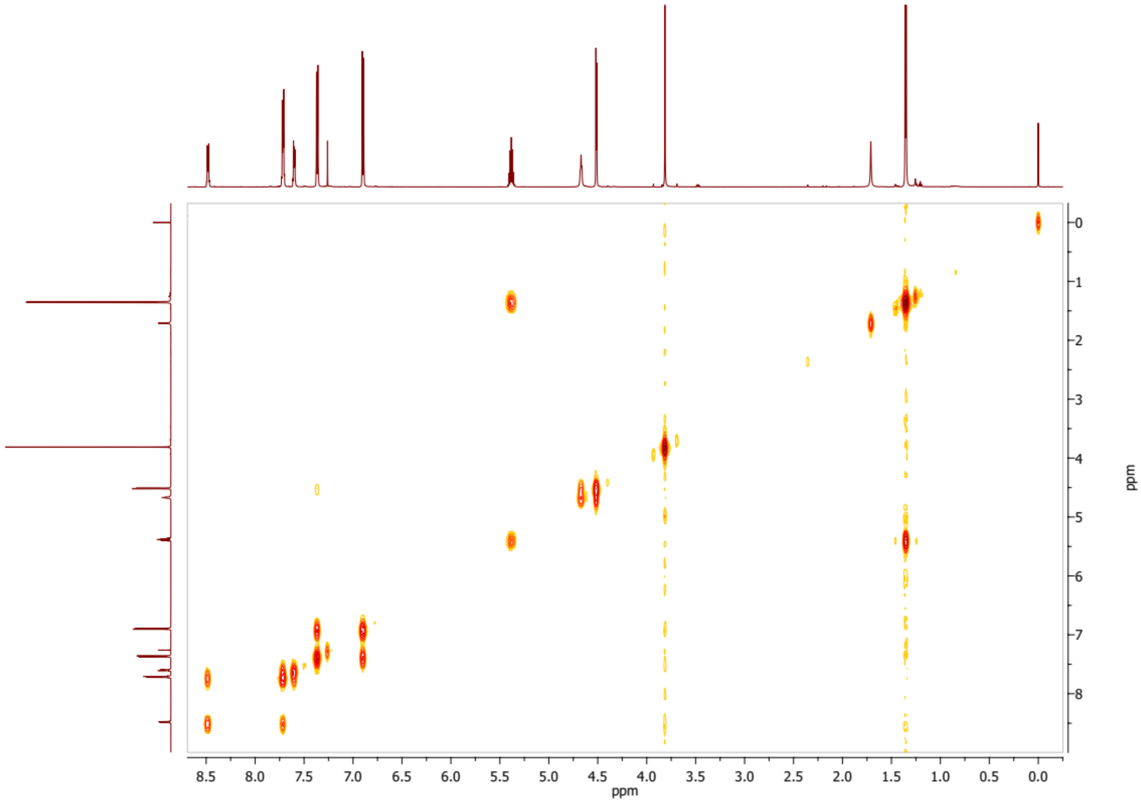

Figure S38: ^{1}H, ^{1}H COSY NMR spectrum of **5h**.

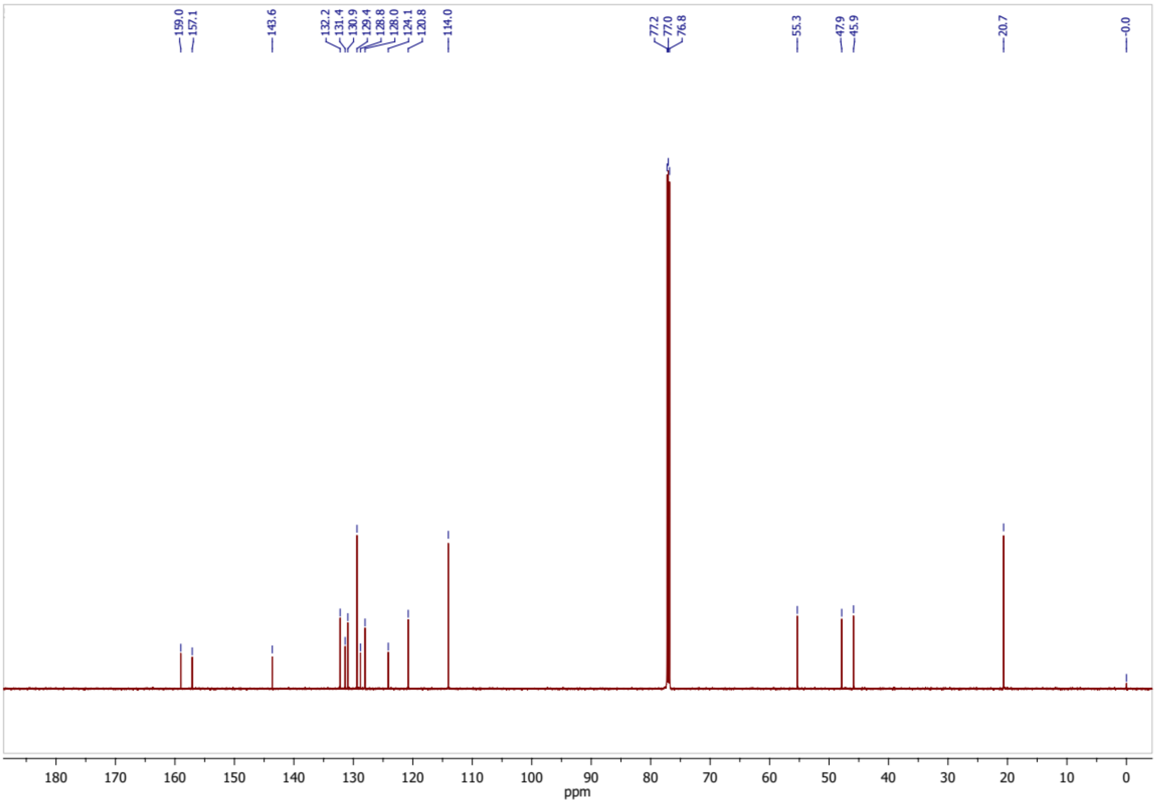

Figure S39: ^{13}C NMR spectrum of **5h**.

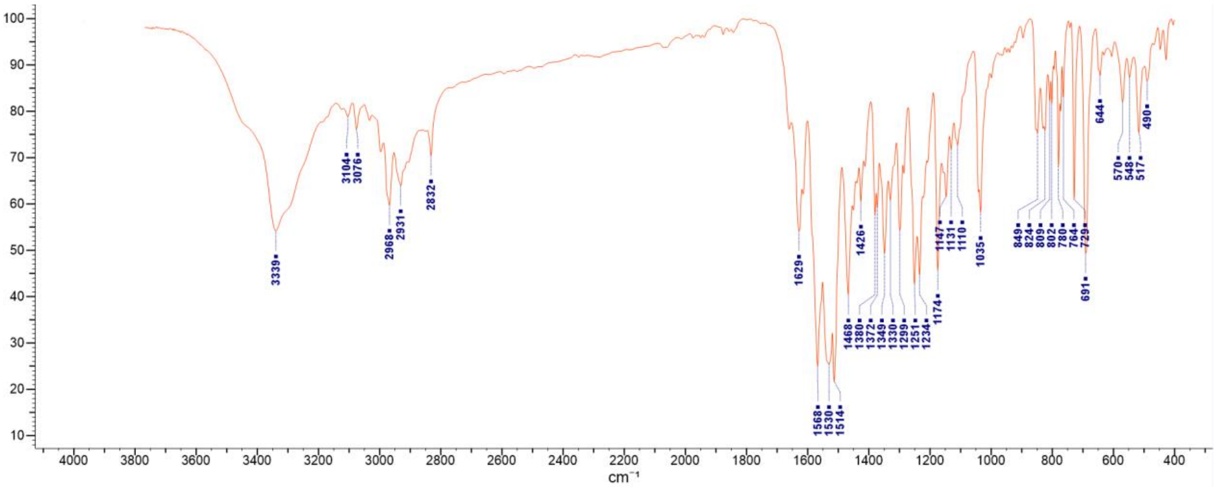

Figure S40: FTIR spectrum of **5h**.

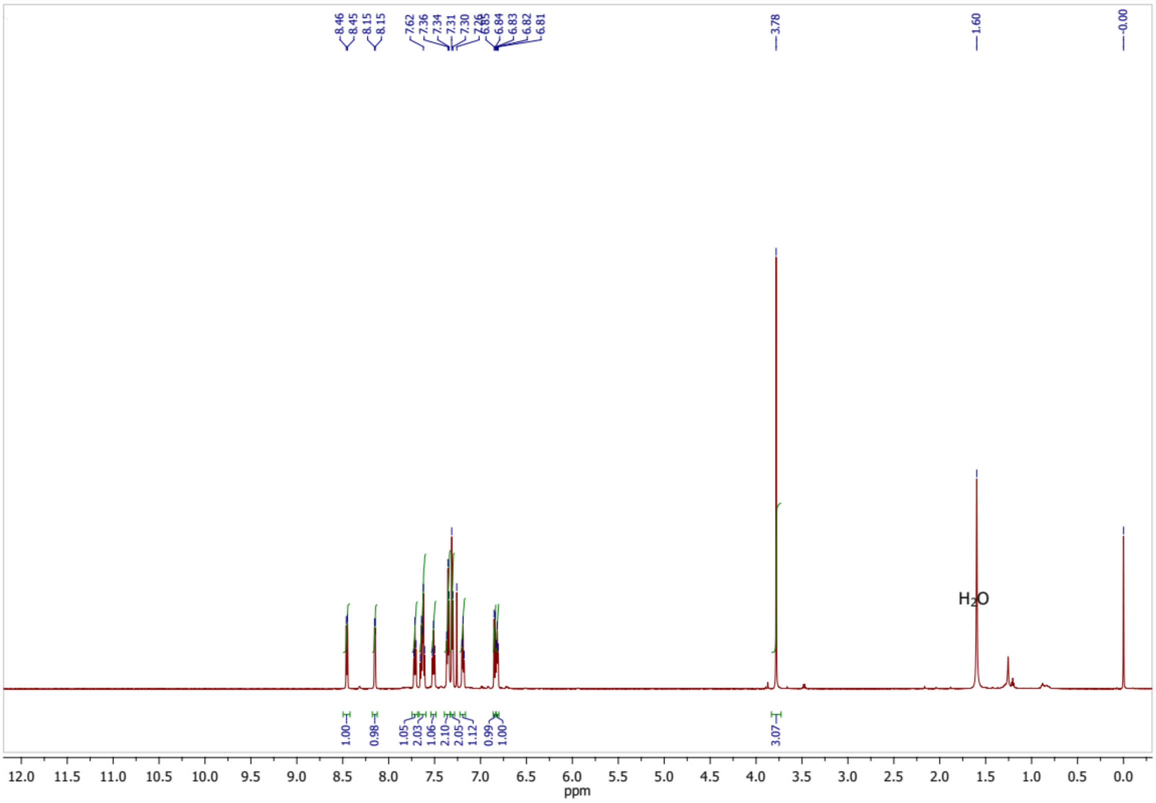

Figure S41: ^{1}H NMR spectrum of **5i**.

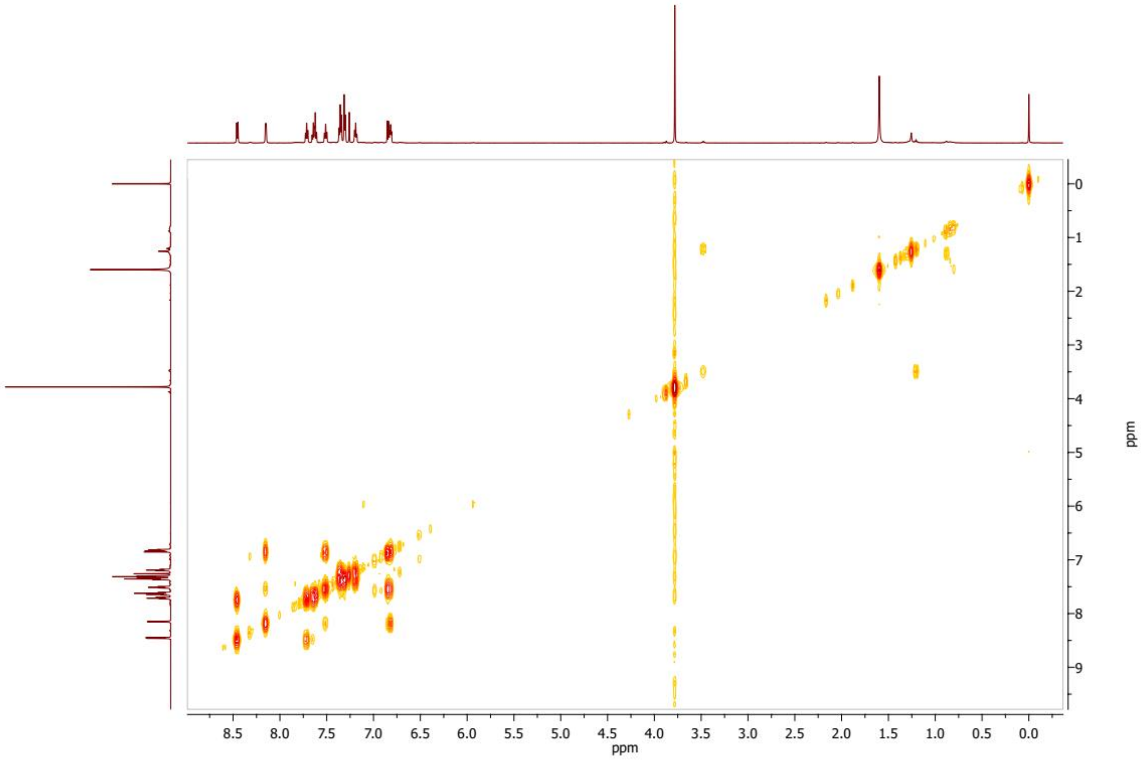

Figure S42: ^{1}H, ^{1}H COSY NMR spectrum of **5i**.

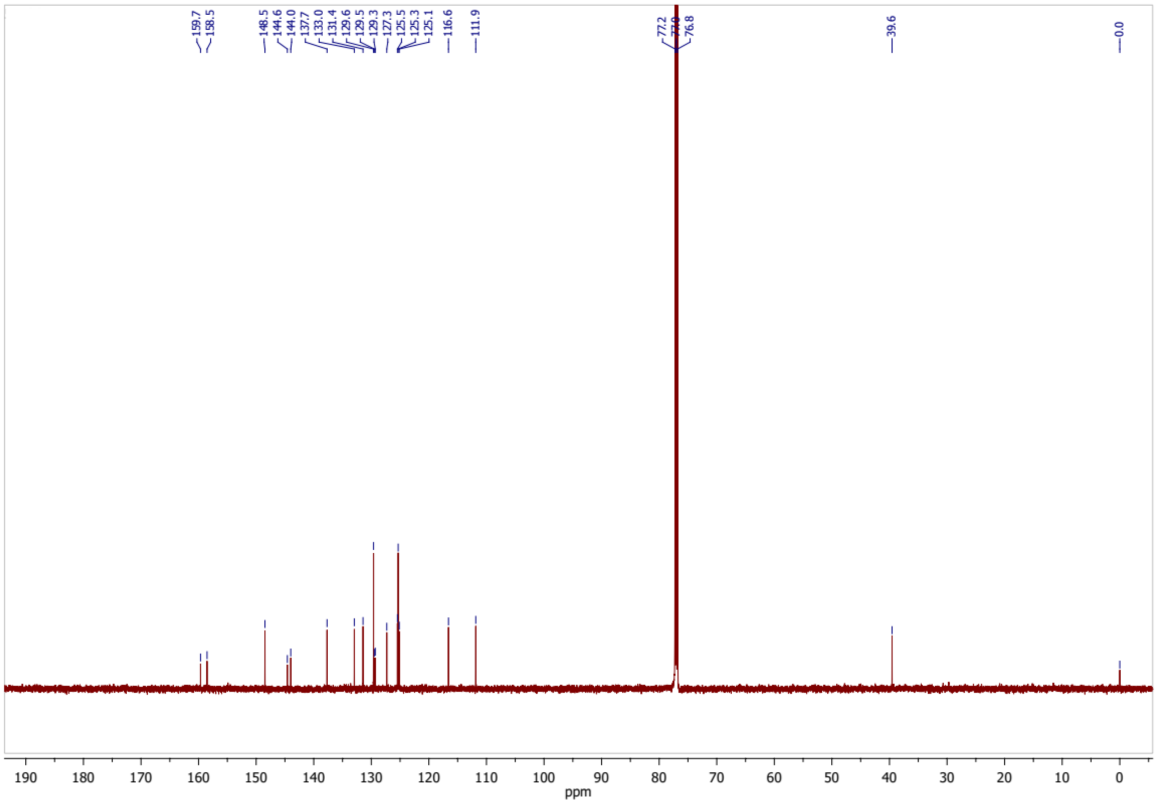

Figure S43: ^{13}C NMR spectrum of **5i**.

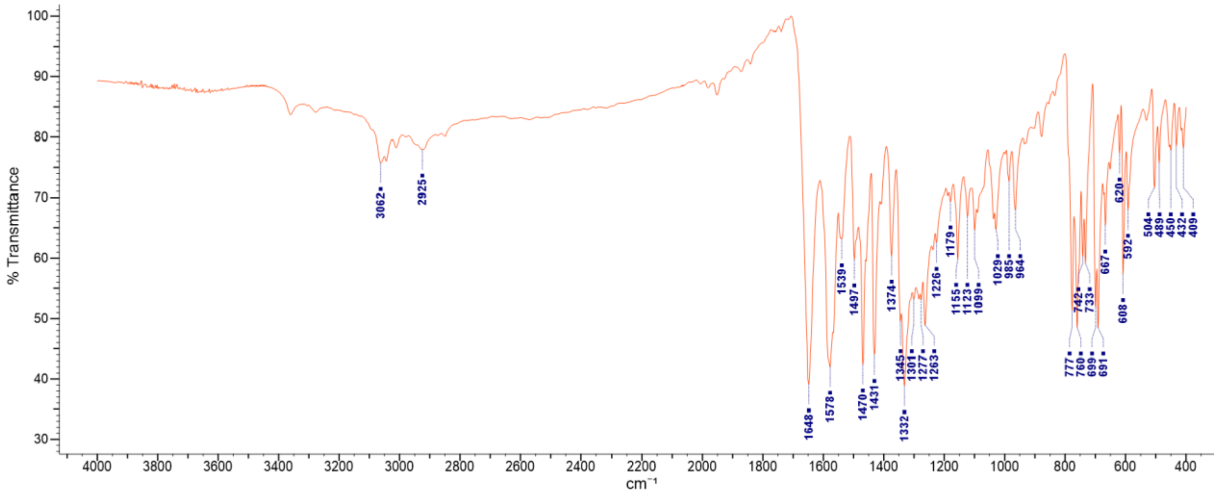

Figure S44: FTIR spectrum of **5i**.

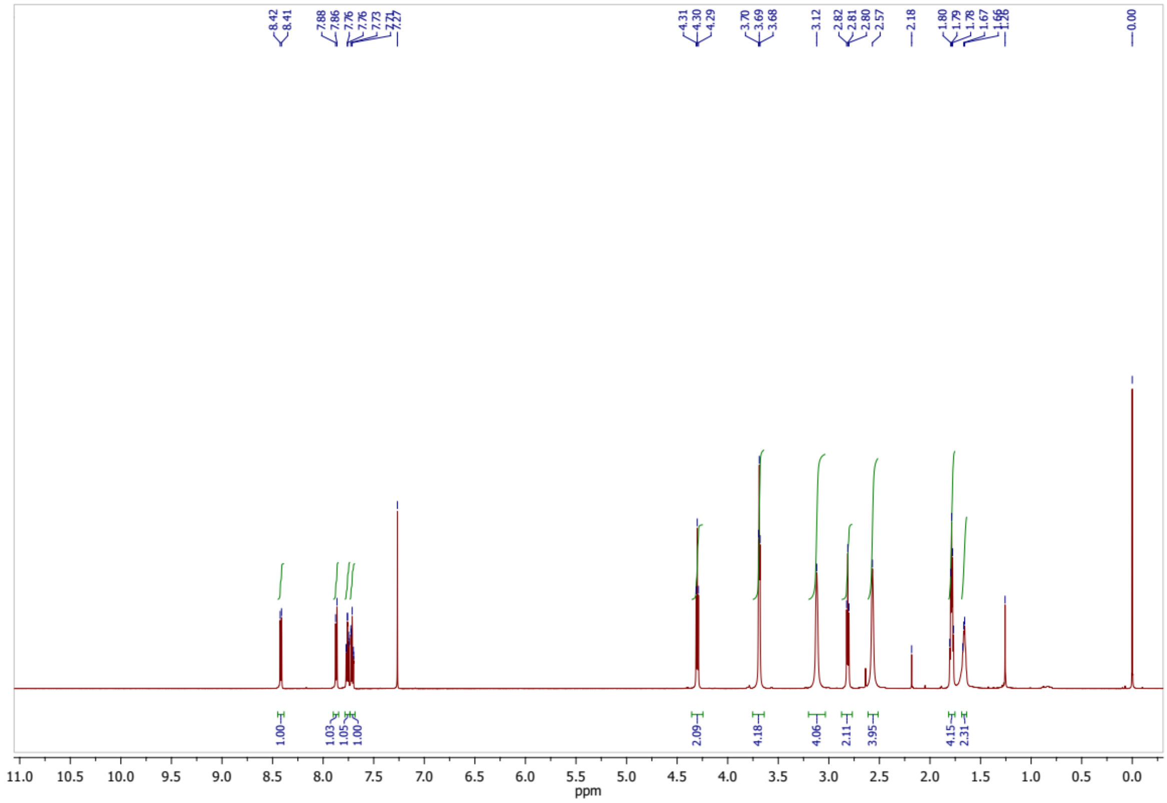

Figure S45: ^{1}H NMR spectrum of **5j**.

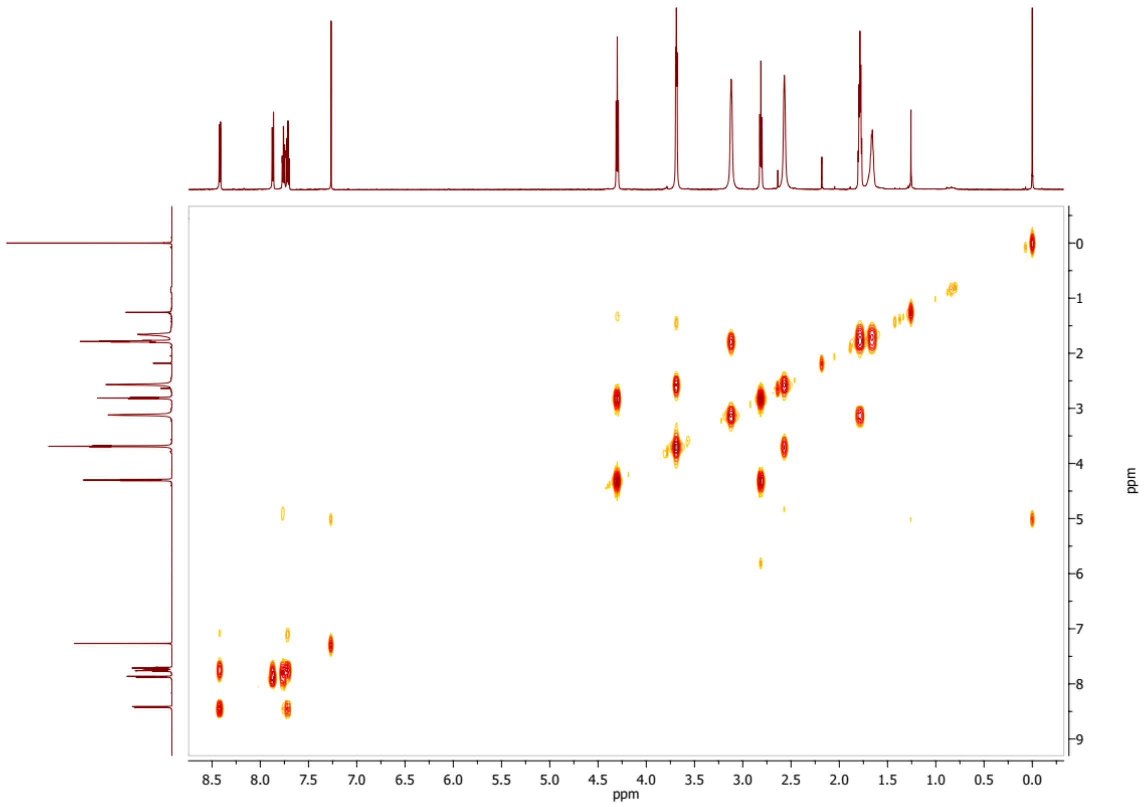

Figure S46: ^{1}H, ^{1}H COSY NMR spectrum of **5j**.

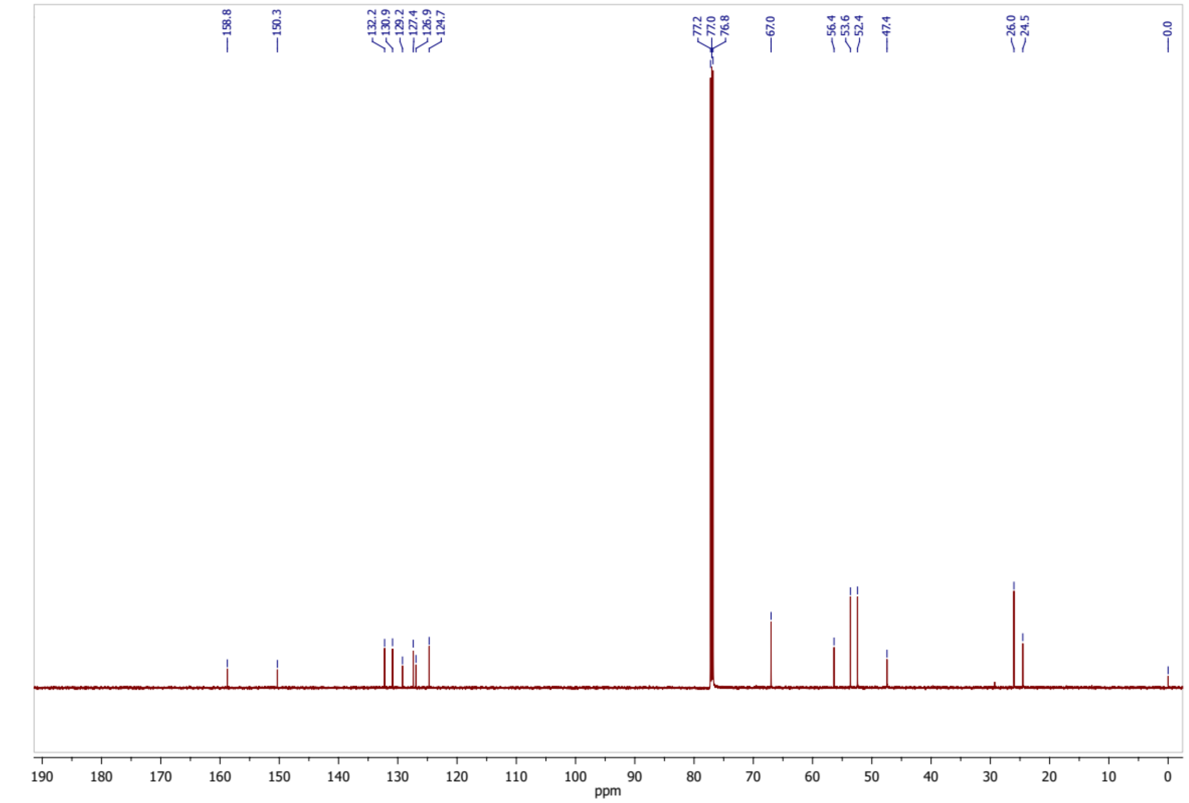

Figure S47: ^{13}C NMR spectrum of **5j**.

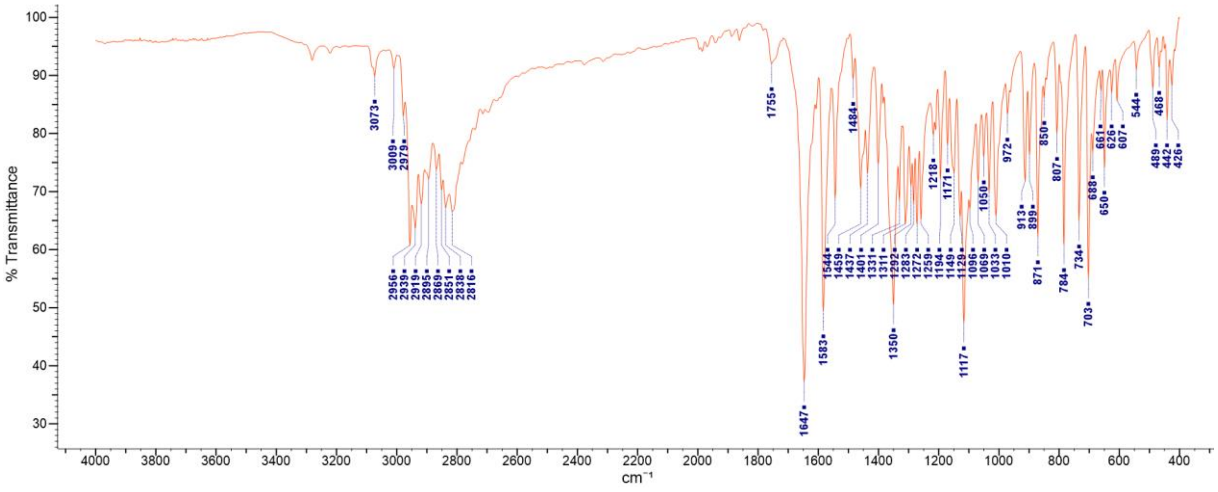

Figure S48: FTIR spectrum of **5j**.

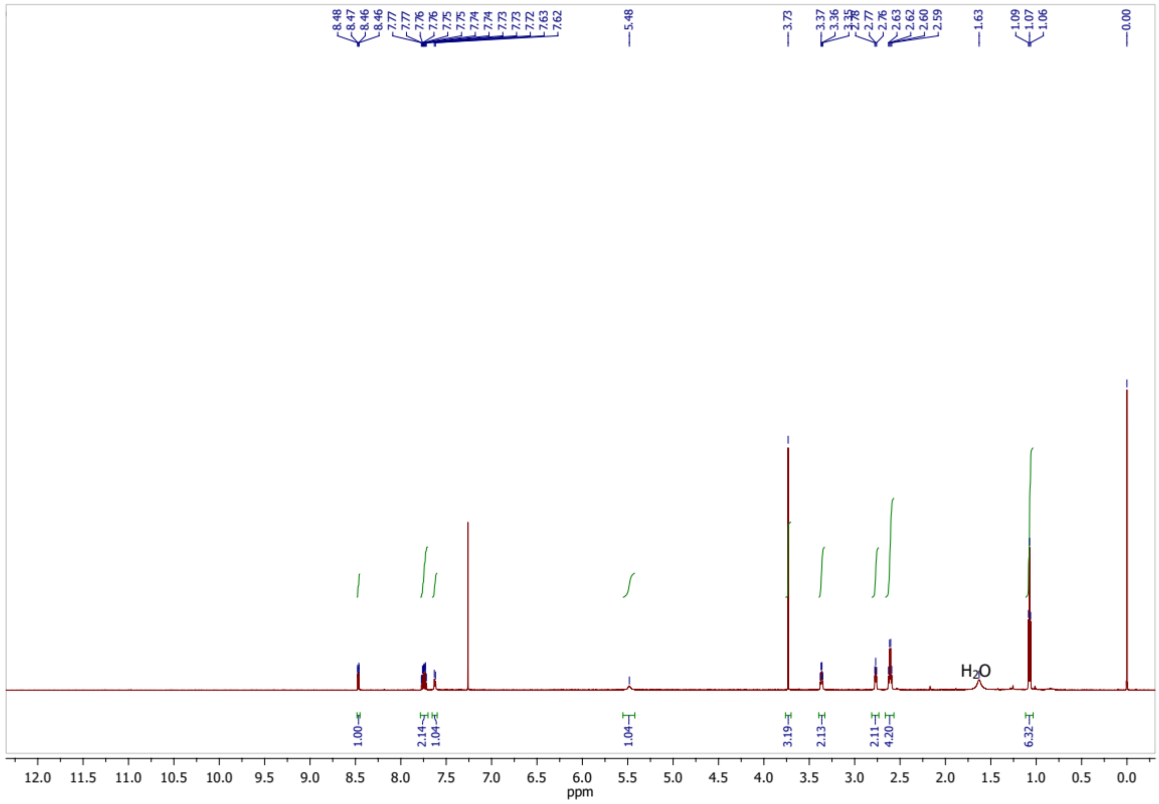

Figure S49: ^{1}H NMR spectrum of **6a**.

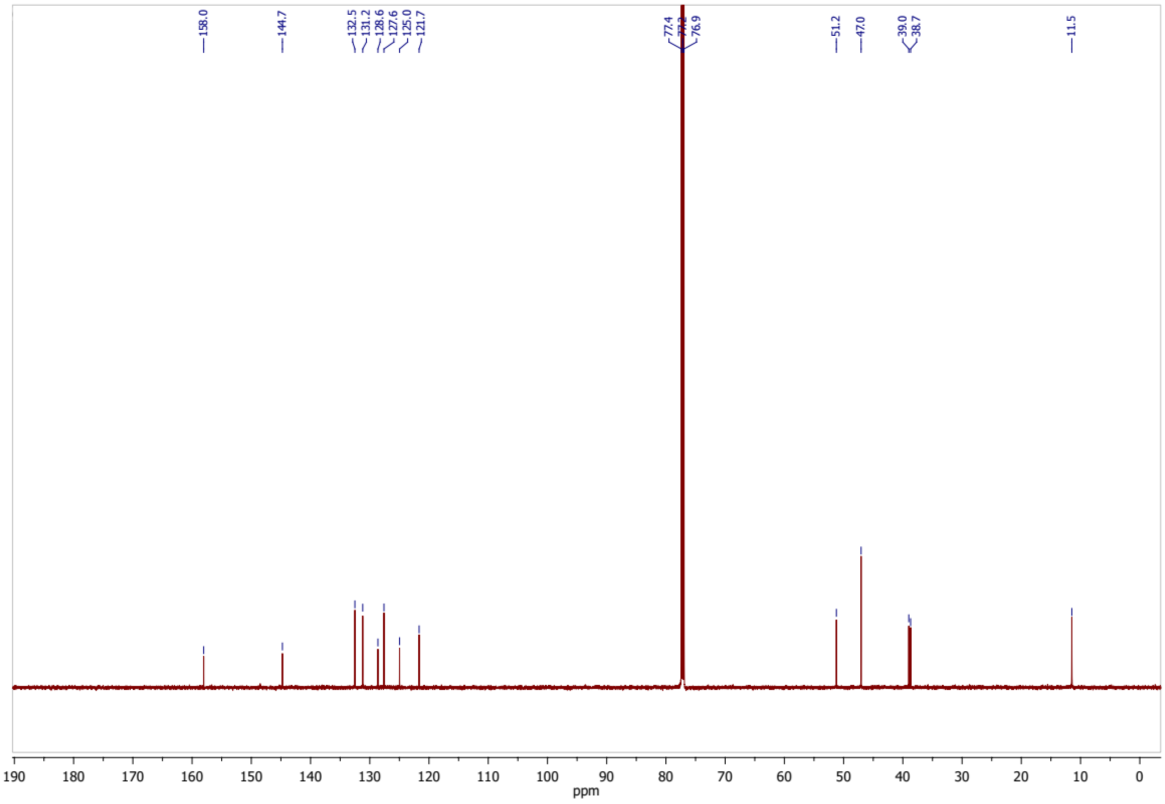

Figure S50: ^{13}C NMR spectrum of **6a**.

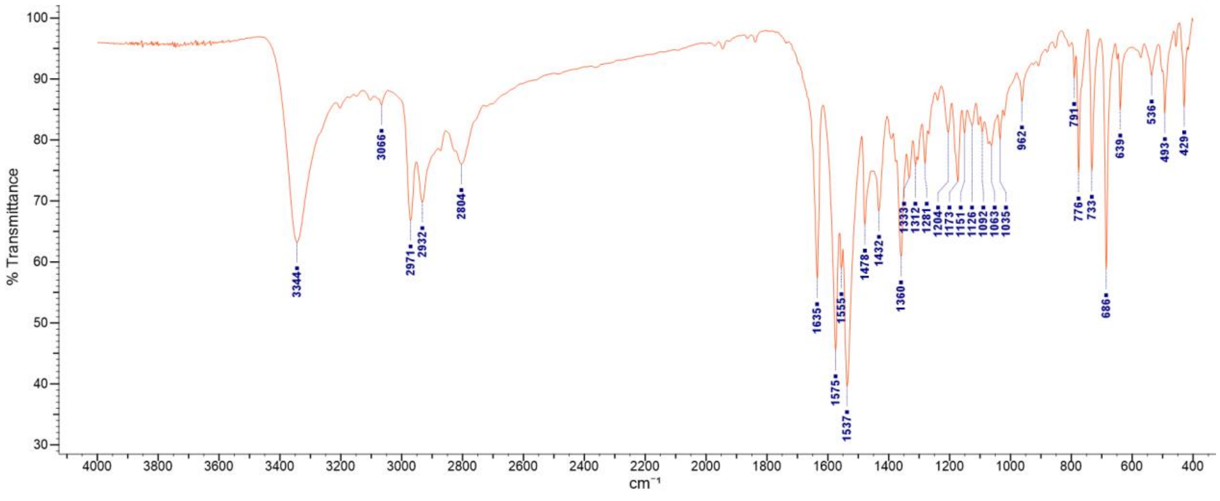

Figure S51: FTIR spectrum of **6a**.

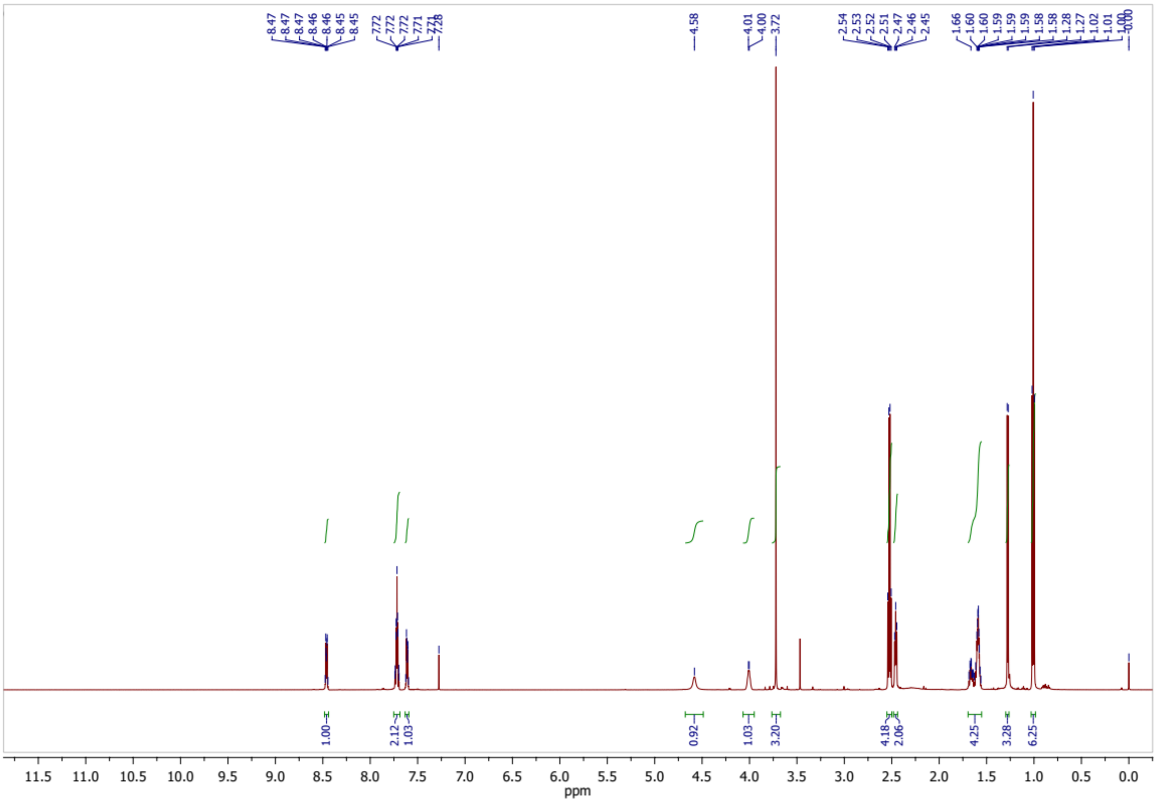

Figure S52: ^{1}H NMR spectrum of **6b**.

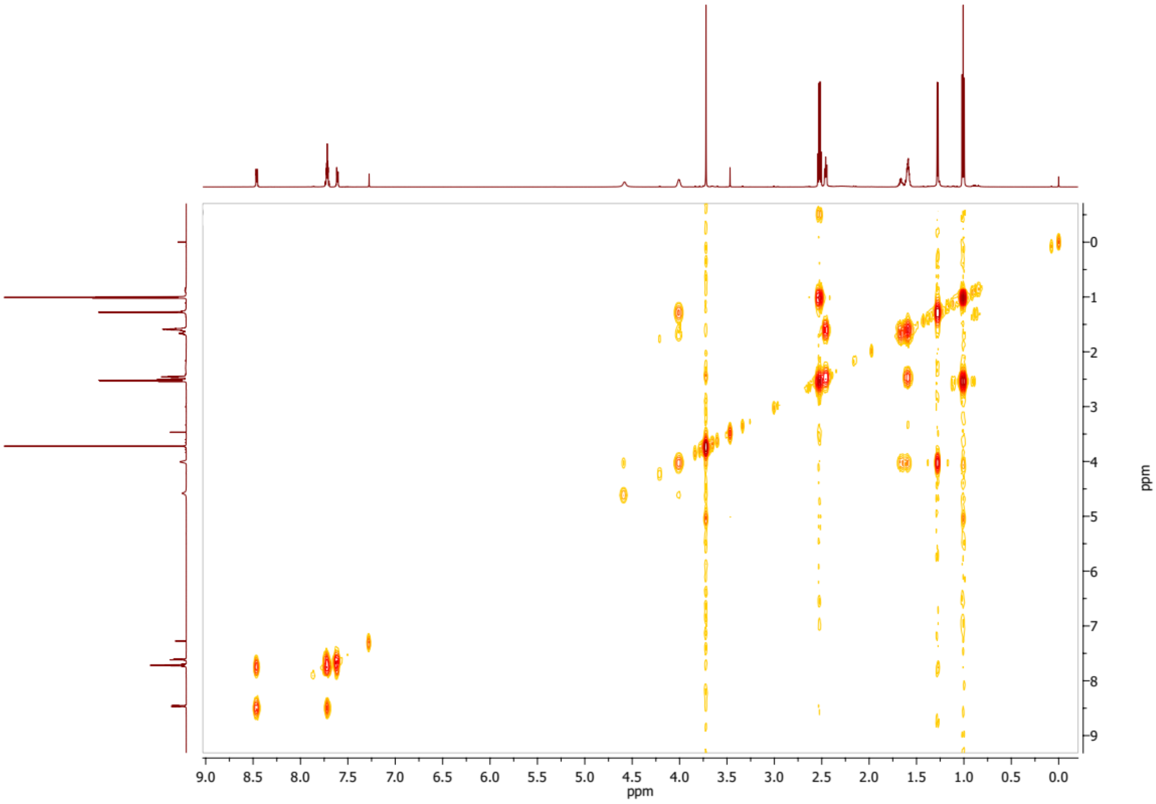

Figure S53: ^{1}H, ^{1}H COSY NMR spectrum of **6b**.

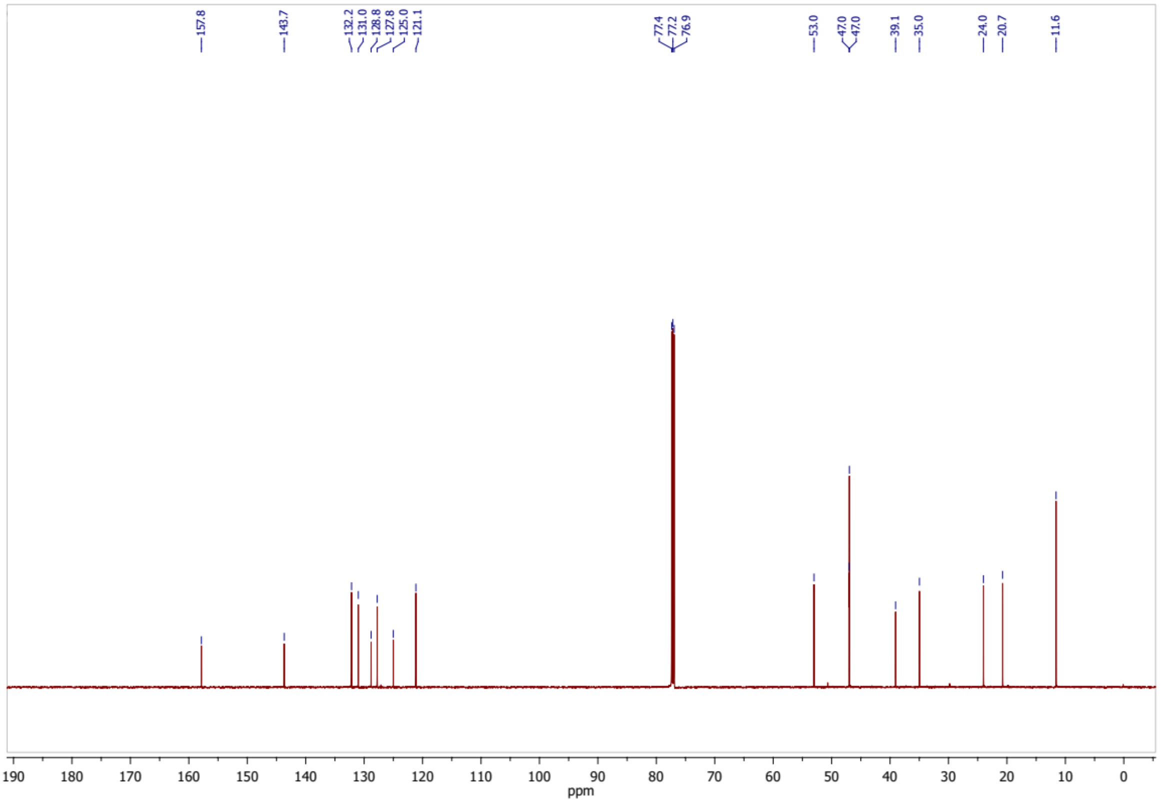

Figure S54: ^{13}C NMR spectrum of **6b**.

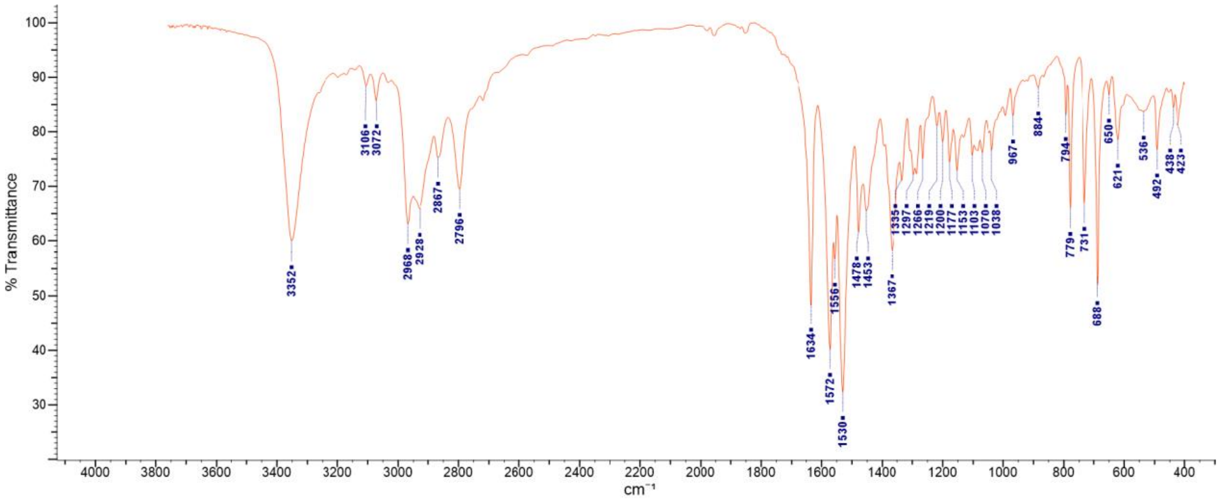

Figure S55: FTIR spectrum of **6b**.

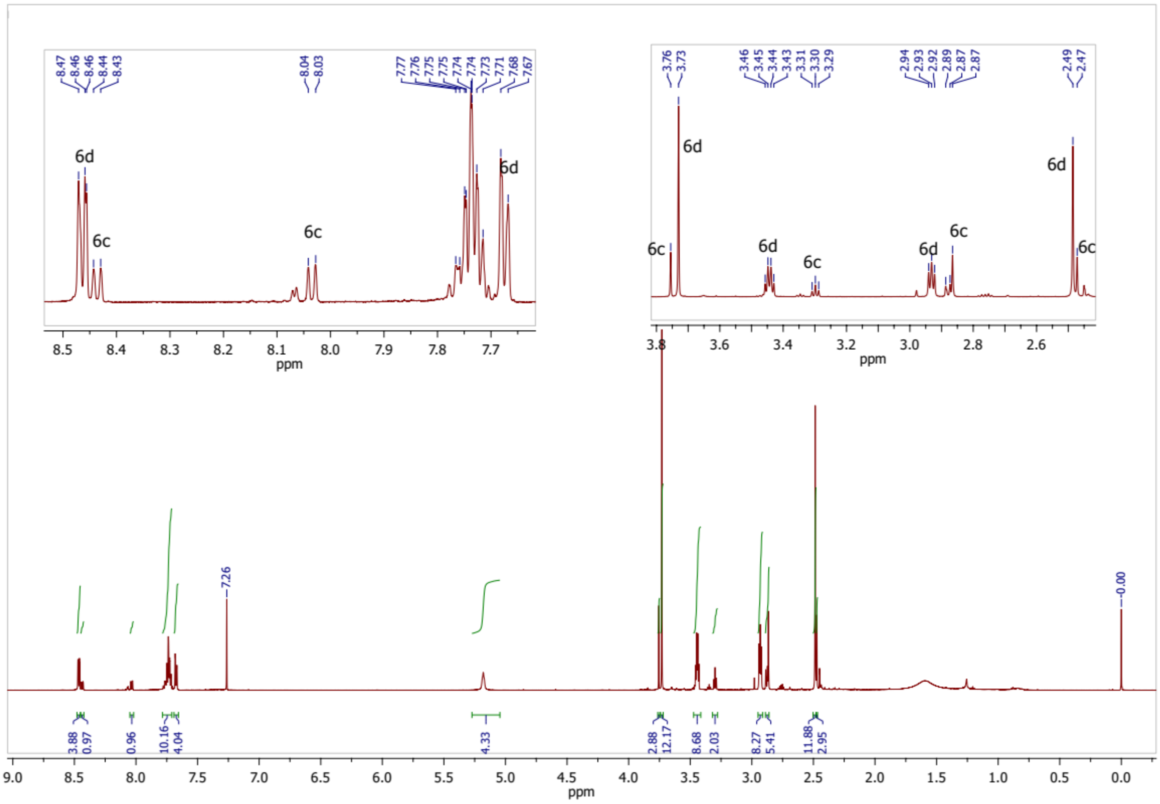

Figure S56: ^{1}H NMR spectrum of **6c** with **6d**.

.

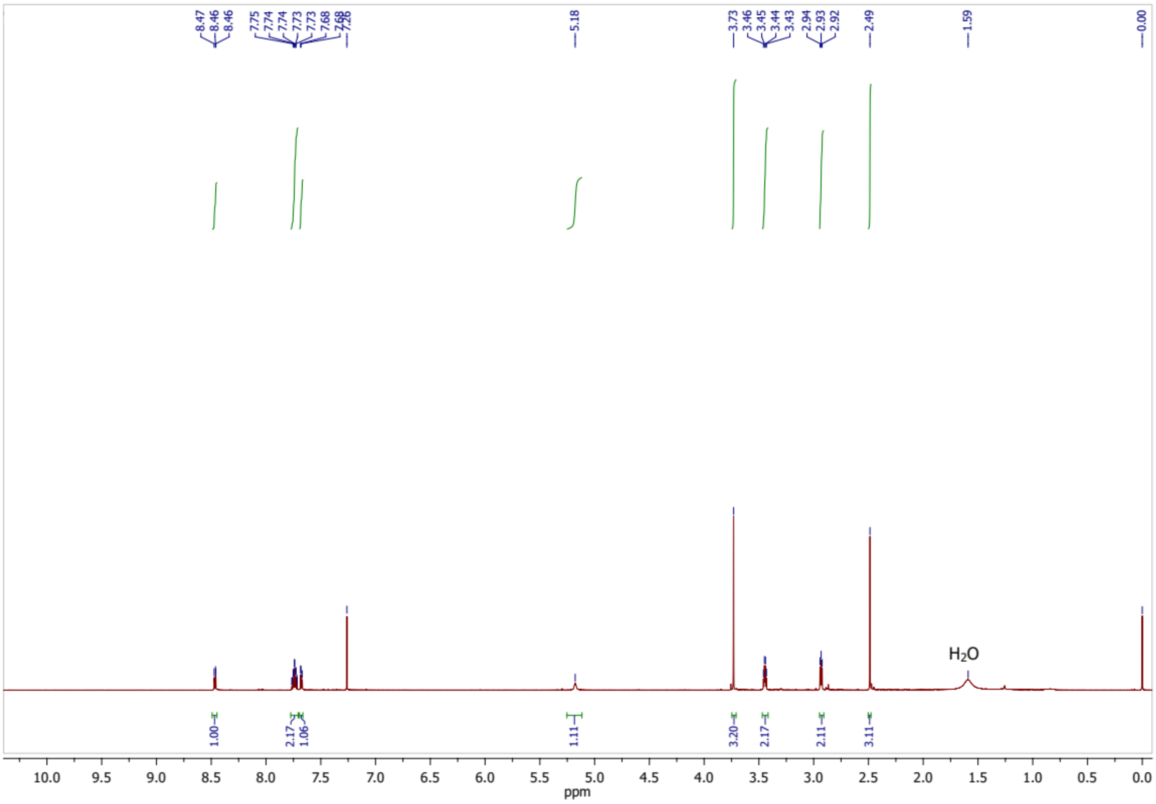

Figure S57: ^{1}H NMR spectrum of **6d**.

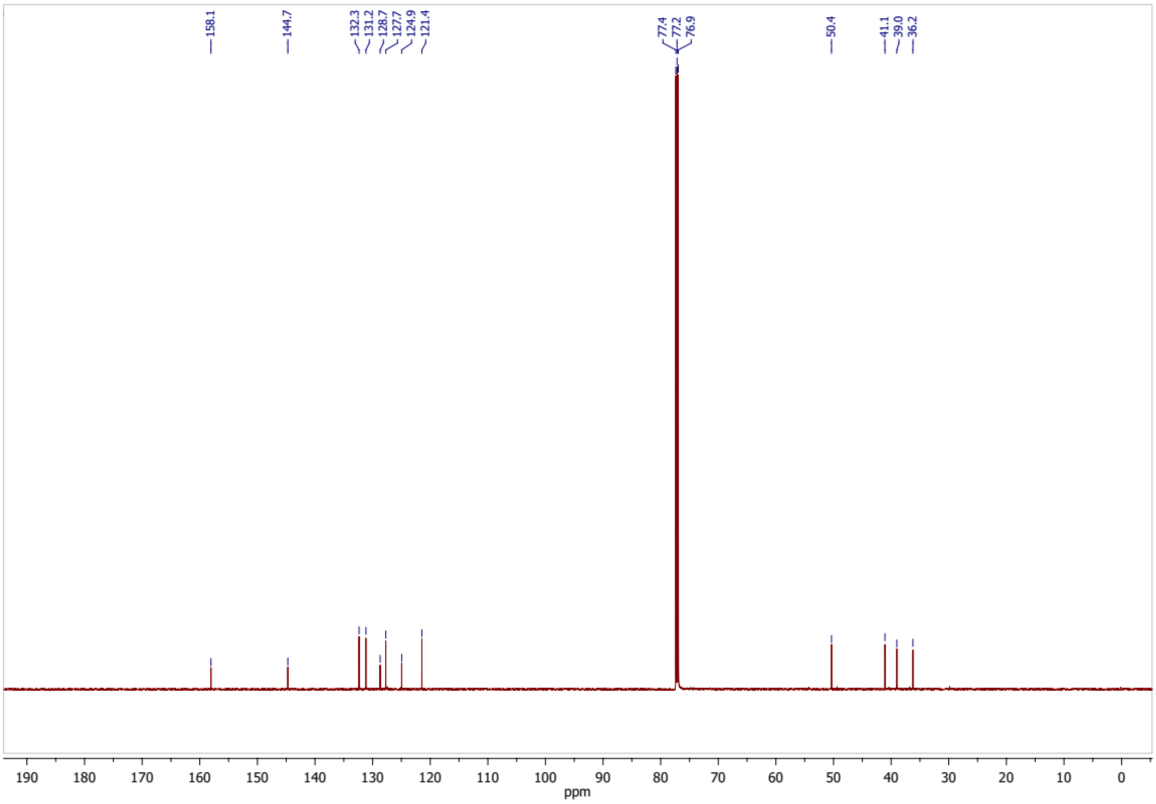

Figure S58: ^{13}C NMR spectrum of **6d**.

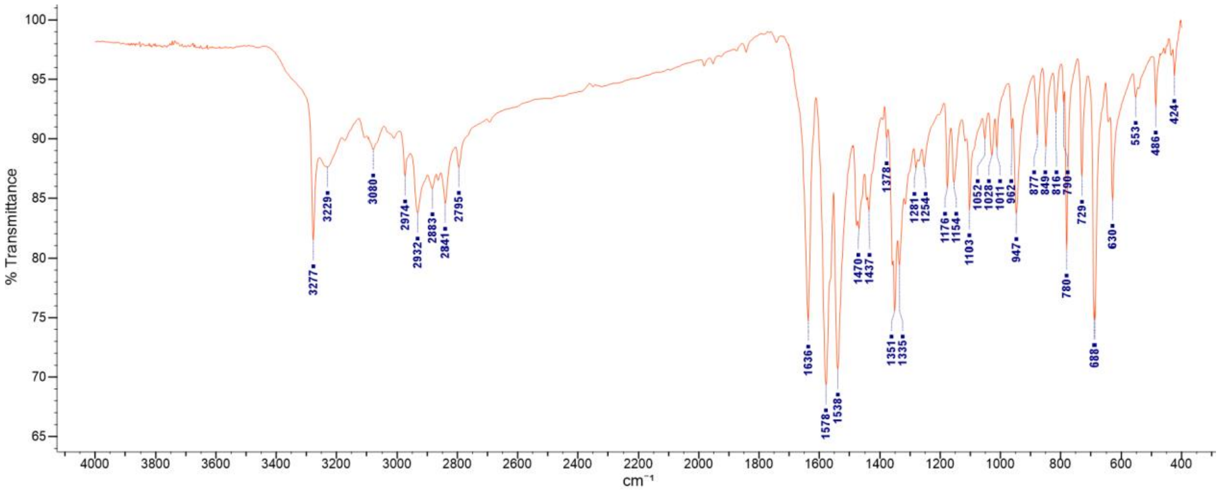

Figure S59: FTIR spectrum of **6d**.

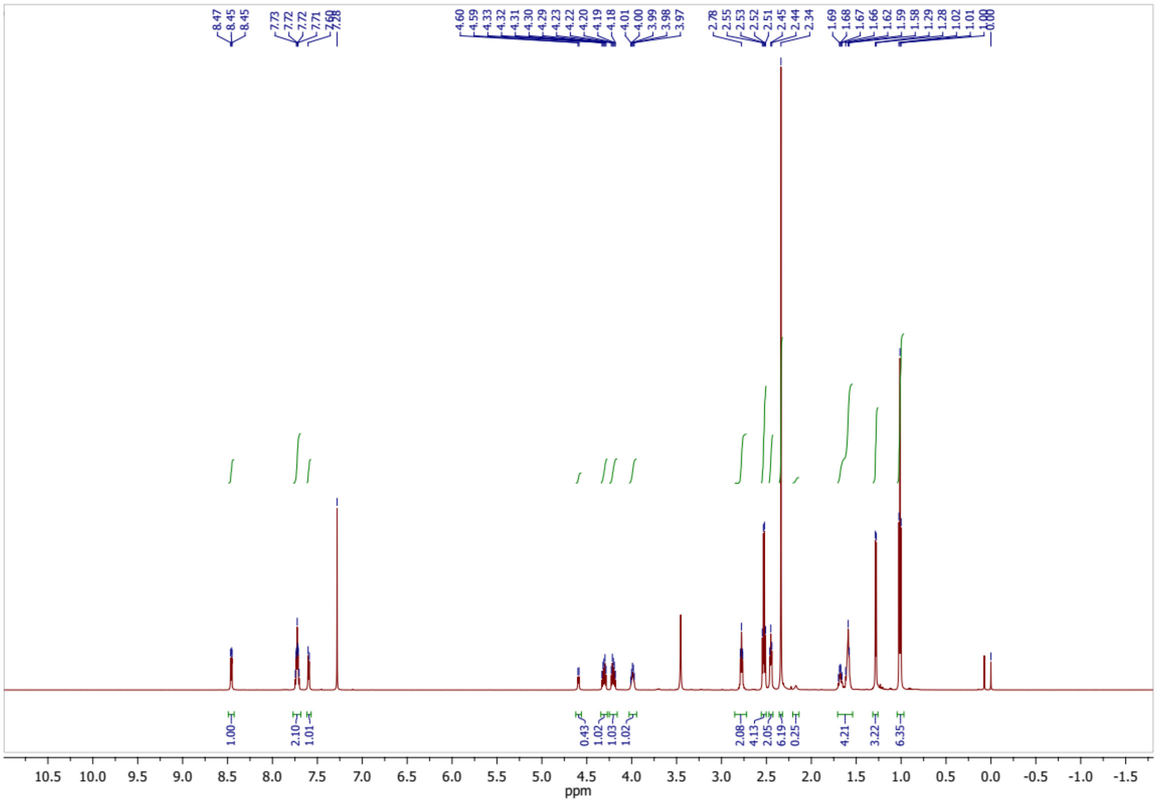

Figure S60: ^{1}H NMR spectrum of **6e**.

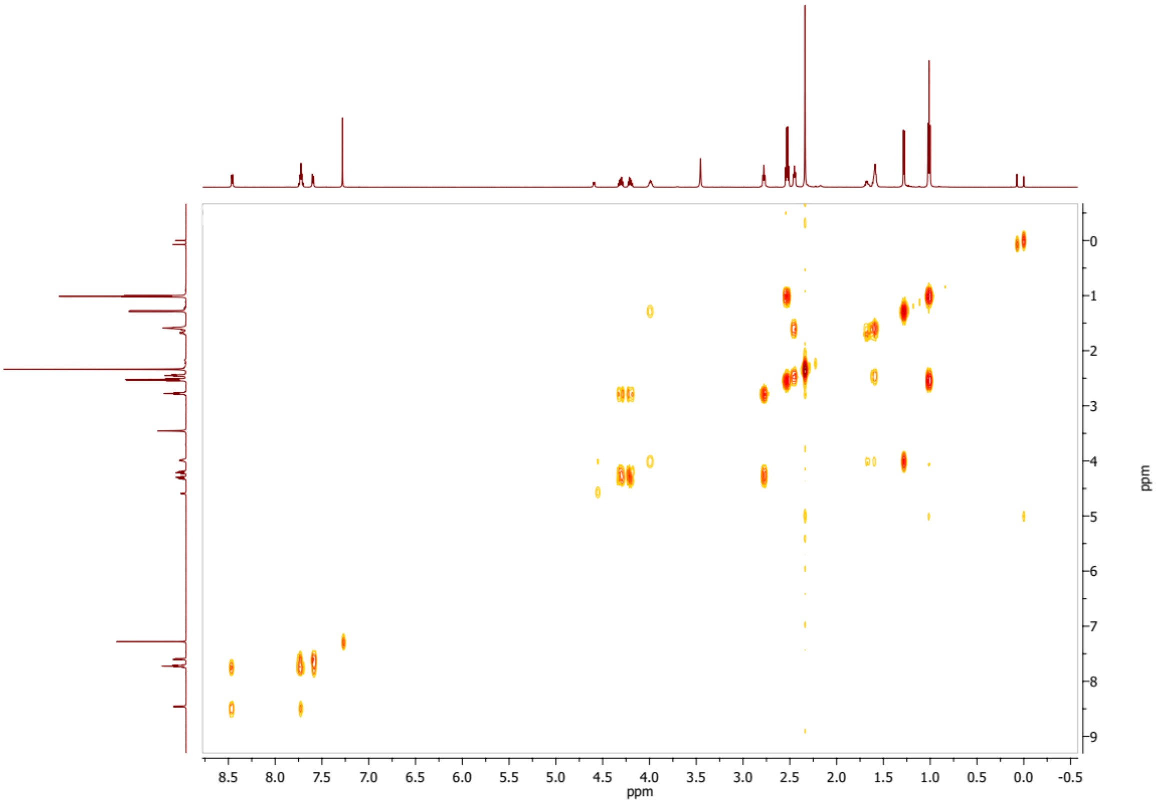

Figure S61: ^{1}H, ^{1}H COSY NMR spectrum of **6e**.

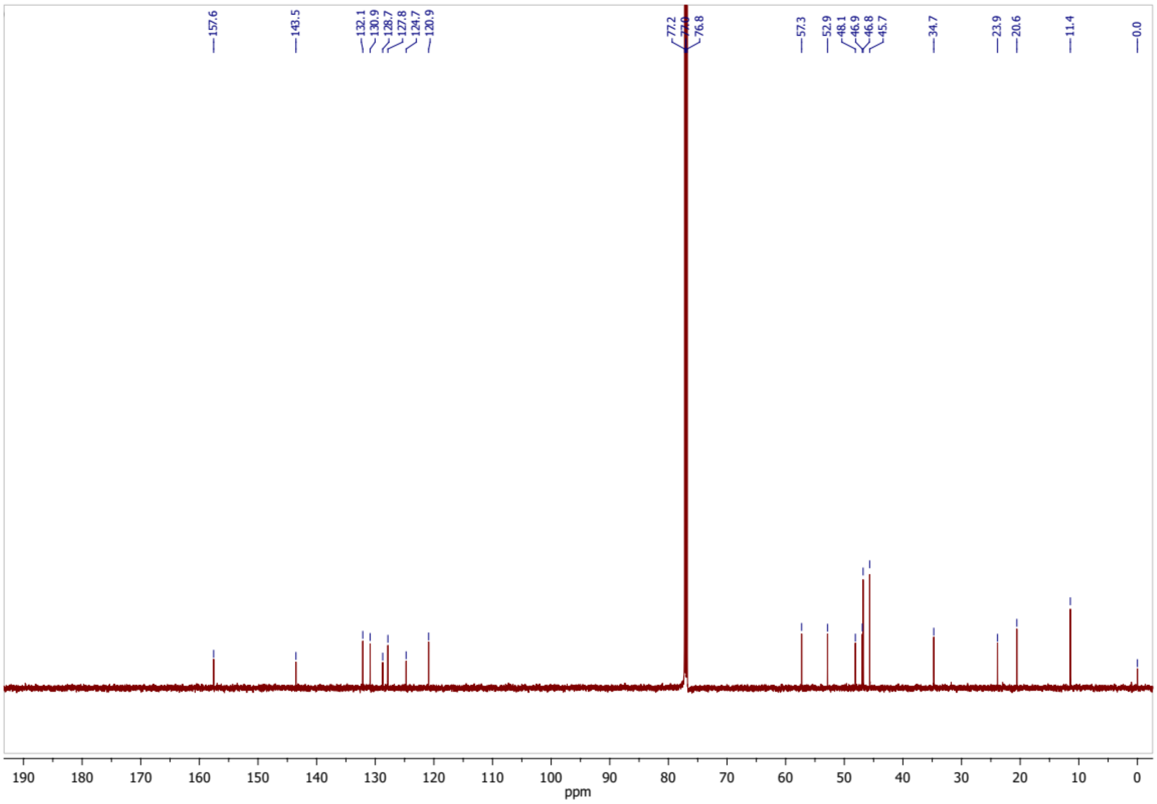

Figure S62: ^{13}C NMR spectrum of **6e**.

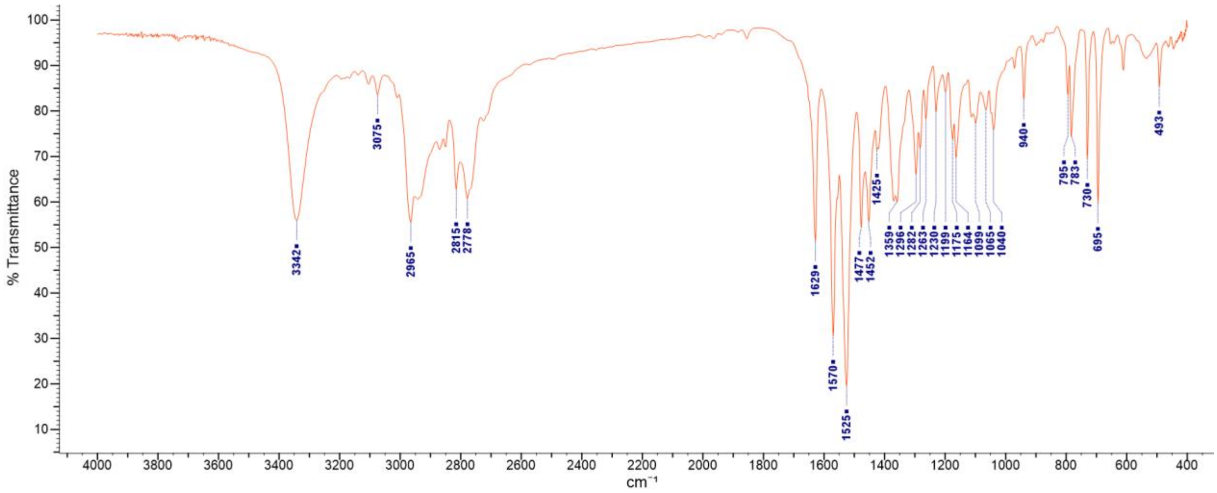

Figure S63: FTIR spectrum of **6e**.
