# Supplementary material for: Amino- and polyaminophthalazin-1(2H)-ones: synthesis, coordination properties, and biological activity
Source: Beilstein J Org Chem. 2021 Feb 25;17:558–68. doi: 10.3762/bjoc.17.50 (PMC7934800; doi:10.3762/bjoc.17.50)
Supplement: File 2 — Experimental details, computational details, X-ray crystallographic data, FTIR and vis–NIR spectra, and cyclic voltammetry for compounds 7 and 17. [file Beilstein_J_Org_Chem-17-558-s002.pdf]

BEILSTEIN JOURNAL OF ORGANIC CHEMISTRY

Supporting Information

for

Amino- and polyaminophthalazin-1(2*H*)-ones: synthesis, coordination properties, and biological activity

Zbigniew Malinowski, Emilia Fornal, Agata Sumara, Renata Kontek, Karol Bukowski, Beata Pasternak, Dariusz Sroczyński, Joachim Kusz, Magdalena Małecka and Monika Nowak

Beilstein J. Org. Chem. **2021,** *17,* 558–568. doi:10.3762/bjoc.17.50

Experimental details, computational details, X-ray crystallographic data, FTIR and vis–NIR spectra, and cyclic voltammetry for compounds 7 and 17

License and Terms: This is a supporting information file under the terms of the Creative Commons Attribution License (https://creativecommons.org/licenses/by/4.0). Please note that the reuse, redistribution and reproduction in particular requires that the author(s) and source are credited and that individual graphics may be subject to special legal provisions.
The license is subject to the *Beilstein Journal of Organic Chemistry* terms and conditions: (https://www.beilstein-journals.org/bjoc/terms)

Table of Contents

1. General information.. S2

2. Synthesis.. S4

3. Crystallography S5

4. FTIR and Vis-NIR spectroscopy S11

5. Cyclic voltammetry S17

3. References... S18

1. General information

X-Ray data for complex **17** were measured using an Agilent SuperNova diffractometer with an Atlas detector at T = 100(2)K with monochromatic MoKα radiation (λ = 0.71073 Å). Multi-scan absorption correction was applied for all data [1]. All structures were solved by direct methods using SHELX and further refined on F^2 using SHELXL-2014/7 [2].

The FTIR spectrum was recorded from a KBr pellet in the range of 4000–400 cm^{-1} with an optical resolution of 4 cm^{-1} using a Nexus FTIR spectrometer (Thermo Nicolet Co., USA).

Vis-NIR spectra were recorded in methanol in the wavelength range of 380–880 nm in a quartz cuvette with the optical path length of 1.0 cm with a Jasco V-630 UV/VIS/NIR spectrophotometer (Jasco Inc., USA).

The cyclic voltammetry measurements were performed in methanol using the three-electrode system with a platinum disk as a working electrode, a platinum gauze as a counter electrode, and a saturated calomel electrode (SCE) as the reference electrode in which the inner solution of KCl was replaced with a saturated NaCl solution to avoid the precipitation of $KClO_4$ in the frit. The electrochemical cell was connected to the potentiostat PGSTAT10 Autolab controlled with GPES v. 4.9 software (both from Eco Chemie BV, The Netherlands). Tetraethylammonium chlorate(VII) was used as a supporting electrolyte, and before each measurement the tested solution was purged with nitrogen gas.

The geometry of complex **17** was fully optimized in vacuum using the DFT method with its crystal structure coordinates as the input geometry. The subsequent vibrational analysis has proved that the stationary point on the potential energy surface was found since no negative frequencies were detected. The calculated vibrational modes were

identified using the Vibrations extension of the Avogadro software [3], and they were scaled with a factor of 0.9688 [4]. The CAM-B3LYP hybrid exchange–correlation functional with the long-range correction was applied [5], and to take into account the relativistic effects, the Lanl2DZ effective core potential basis set [6] was used for the Cu atom, and the standard all-electron 6-311++(d,p) basis set was chosen for H, C, N, O, and Cl atoms (hereafter denoted as 6-311++(d,p)/Lanl2DZ(Cu)). After full geometry reoptimization of complex **17** in implicit methanol solvent, its vis-NIR spectrum was simulated using the TD-DFT method through the calculation of the vertical transition energies to the first 30 low-lying singlet excited states in methanol as well. The implicit methanol environment in the DFT and TD-DFT calculations was approximated using the Integral Equation Formalism of the Polarizable Continuum Model (IEFPCM) [7]. The atomic orbital contributions to molecular orbitals were calculated using the AllOrbitals option of the Gaussian Population keyword. All DFT and TD-DFT calculations were performed using Gaussian 09 (Rev. D.01) package [8].

Potassium bromide, copper(II) chloride dihydrate, tetraethylammonium chlorate(VII), and methanol were purchased from Sigma-Aldrich or Chempur (Poland) and used without further purification.

2. Synthesis

2.1 Synthesis of 2-[2-(Dimethylamino)ethyl]-6-methoxy-4-(pyridin-2-yl)phthalazin-1(2*H*)-one (**7**)

Compound **7** (yellow solid; yield 64%.) was synthesized according to the procedure described in our previous report [9]. The ^{1}H, ^{13}C NMR and FTIR data are in agreement with those reported in the literature [9].

2.2 Synthesis of complex **17**

A solution of $CuCl_2 \cdot 2H_2O$ (0.0171 g, 0.1 mmol) in methanol (20 mL) was added dropwise to a stirred solution of phthalazinone derivative **7** (0.0324 g, 0.1 mmol) in methanol (50 mL). The stirring was continued at ambient temperature for 4 h and after this time solution was allowed at this temperature to a very slow evaporation of the solvent (ca. two weeks). In this way the light green crystals suitable for X-ray analysis were obtained. Yield: 0.0298 g, 65%. FT-IR (KBr): ν = 3439, 3075, 1660, 1608, 1578, 1531, 1472, 1382, 1331, 1289, 1241 cm^{-1}. Vis-NIR (methanol): λ_{max} = 710 nm, $\varepsilon = 3067$ dm^3 / (mol cm).

3. Crystallography

All non-hydrogen atoms were refined anisotropically. The positions of hydrogen atoms were calculated from the known geometry (C–H bond lengths at 0.95, 0.98, and 0.99 Å for aromatic CH, methyl Me, and methylene CH_2 atoms, respectively) and treated as riding where the isotropic thermal parameters of these hydrogen atoms were fixed as $U_{iso}(H) = 1.5U_{eq}(C)$ for H atoms from methyl group and $U_{iso}(H) = 1.2U_{eq}(C)$ for all remaining atoms.

3.1 X-Ray single-crystal structure of complex **17**.

Table S1: Experimental and theoretical parameters for molecules A and B of complex **17**.

		Exp.	Calc.[a]
Molecule A		Bond lengths (Å)	
	Cu1-Cl1	2.2905(17)	2.39417
	Cu1-Cl2	2.4175(19)	2.34360
	Cu1-N1	2.176(5)	2.39417
	Cu1-N3	1.995(5)	2.07000
	Cu1-N4	2.008(5)	2.14137
		Bond angels (°)	
	Cl1-Cu1-Cl2	117.2(2)	146.36652
	Cl1-Cu1-N1	143.7(2)	105.32247
	Cl1-Cu1-N3	92.8(2)	91.08575
	Cl1-Cu1-N4	93.4(2)	96.35414

	Cl2-Cu1-N1	97.6(2)	107.02706
	Cl2-Cu1-N3	86.7(2)	88.98975
	Cl2-Cu1-N4	97.0(2)	94.79740
	N1-Cu1-N3	78.6(2)	74.22834
	N1-Cu1-N4	92.1(2)	86.05216
	N3-Cu1-N4	170.4(2)	160.15522

Molecule B		Bond lengths (Å)	
	Cu2-Cl3	2.2838(17)	2.28783
	Cu2-Cl4	2.4250(17)	2.31713
	Cu2-N5	2.005(5)	2.05167
	Cu2-N6	2.025(5)	2.10403
	Cu2-N7	2.178(5)	2.36625
		Bond angels (°)	
	N6-Cu2-N7	91.0(2)	86.11765
	Cl3-Cu2-N5	94.2(2)	90.39575
	Cl3-Cu2-N6	93.5(2)	96.58238
	Cl3-Cu2-N7	143.8(2)	103.21391
	Cl4-Cu2-N5	89.0(2)	88.78492
	Cl4-Cu2-N6	95.5(2)	94.93956
	Cl4-Cu2-N7	102.3(2)	108.31446
	Cl3-Cu2-Cl4	113.0(2)	147.02828
	N5-Cu2-N7	78.0(2)	74.70961

	N5-Cu2-N6	168.8(2)	160.65503

[a]Theoretical geometrical parameters were calculated at the CAM-B3LYP/6–311++G(d,p)/LanL2DZ(Cu) level of theory in vacuum.

The calculated five-coordinate geometry index $\tau_5 = (\beta-\alpha)/60°$ [10], where β and α are two greatest bond angles X–M–Y (X, Y = donor atoms of ligand), for the CuN_3Cl_2 coordination center of molecules A and B is equal to 0.45 (β(N3-Cu1-N4) = 170.4°, α(Cl1-Cu1-N1) = 143.7°) and 0.42 (β(N5-Cu2-N6) = 168.8°, α(Cl3-Cu2-N7) = 143.8°), respectively. Since the calculated τ_5 are almost in the middle of the range for an ideal square pyramid (τ_5 = 0.0) and for an ideal trigonal bipyramid (τ_5 = 1.0), the geometry of the CuN_3Cl_2 coordination center of the complex **17** can be considered as the strongly distorted square pyramidal. The basal plane of the square-pyramid is formed by N1, N3, N4, and Cl1 atoms in the molecule A, and by N5, N6, N7, and Cl3 atoms in the molecule B, and the apex positions are occupied by Cl2 and Cl4 anions, respectively. In molecule A the Cl1 anion is deviated from the plane formed by N1, N3 and N4 atoms by 1.253 Å, and in molecule B the Cl3 atom is deviated from the plane formed by N5, N6 and N7 atoms by 1.257 Å. In both molecules A and B the axial Cu-Cl distances, Cu1-Cl2 = 2.4175(19) Å and Cu2-Cl4 = 2.4250(17) Å, are longer than the equatorial Cu-Cl distances, Cu1-Cl1 = 2.2905(17) Å and Cu2-Cl3 = 2.2838(17) Å, and the corresponding Cu-N bond lengths follow the same order $Cu\text{-}N_{azomethine}$ > $Cu\text{-}NMe_2$ > Cu-N(2Py). In molecules A and B the corresponding N-Cu-N, N-Cu-Cl, and Cl-Cu-Cl bond angles also display a high similarity as their differences are in the range of 0.6–1.6, 0.1–4.7, and equal to 4.2°, respectively.

The crystal structure of the complex **17** is stabilized with a 3D intermolecular hydrogen bond network (Figure S1a). In this hydrogen bond system the aliphatic (H13A, H15C, and H36B) or aromatic (H2, H10, and H26) hydrogen atoms act as proton donors,

whereas the chloride coligands (Cl1 and Cl3) and methoxy (O3) and carbonyl (O4) oxygen atoms function as proton acceptors. These weak C–H⋯O and C–H⋯Cl hydrogen bonds are characterized by the H⋯A lengths in the range of 2.40–2.59 and 2.73–2.82 Å, respectively (Table S2). They also display a middle donor directionality, i.e., preference for linearity, as their bond angles are in the range of 147–174° as expected for hydrogen-bond donors with a middle electronegativity [11].

Complex **17** in a solid state is also stabilized by π–π stacking interactions between the 1,2-diazine moieties and the pyridin-2-yl substituents of the ligands (Figure S1b). The calculated Cg1⋯Cg3 and Cg2⋯Cg4 distances between the corresponding centroids are equal to 3.589(2) and 3.536(2) Å, respectively. The moieties defining the Cg1⋯Cg3 and Cg2⋯Cg4 interactions adopt slightly parallel-displaced and sandwich conformations, respectively, and they are almost parallel as the dihedral angles between their calculated mean planes are equal to 3.48 and 1.48°, respectively. The experimental and calculated bond lengths and angles of the CuN_3Cl_2 coordination center of complex **17** are compared in Table S1. In general, the calculated Cu-N and Cu-Cl bond lengths in molecules A and B are overestimated by 0.08–0.22 and 0.05–0.19 Å, respectively, except for the Cu1-Cl2 and Cu2-Cl4 bonds which are underestimated by 0.07 and 0.11 Å, respectively. On the other hand, the calculated the N-Cu-N, N-Cu-Cl, and Cl-Cu-Cl bond angles are overestimated or underestimated, however, the largest deviation from the experimental values display the Cl1-Cu1-N1 and Cl3-Cu2-N7 bond angles which differ by 38.4 and 40.6°, respectively. This can be explained by the fact that in the solid state the Cl1 and Cl3 atoms are involved in intramolecular hydrogen bonding (Figure S1a) which significantly affects the geometry of the coordination center and these interactions were not included in the DFT calculations in vacuum.

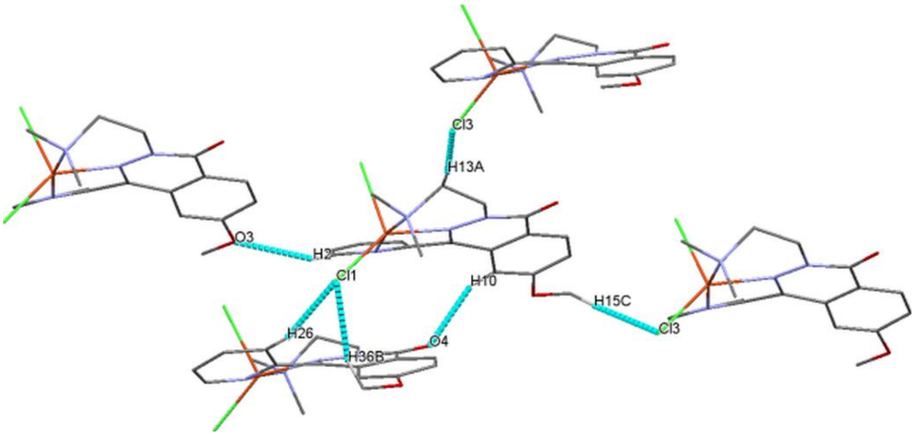

a)

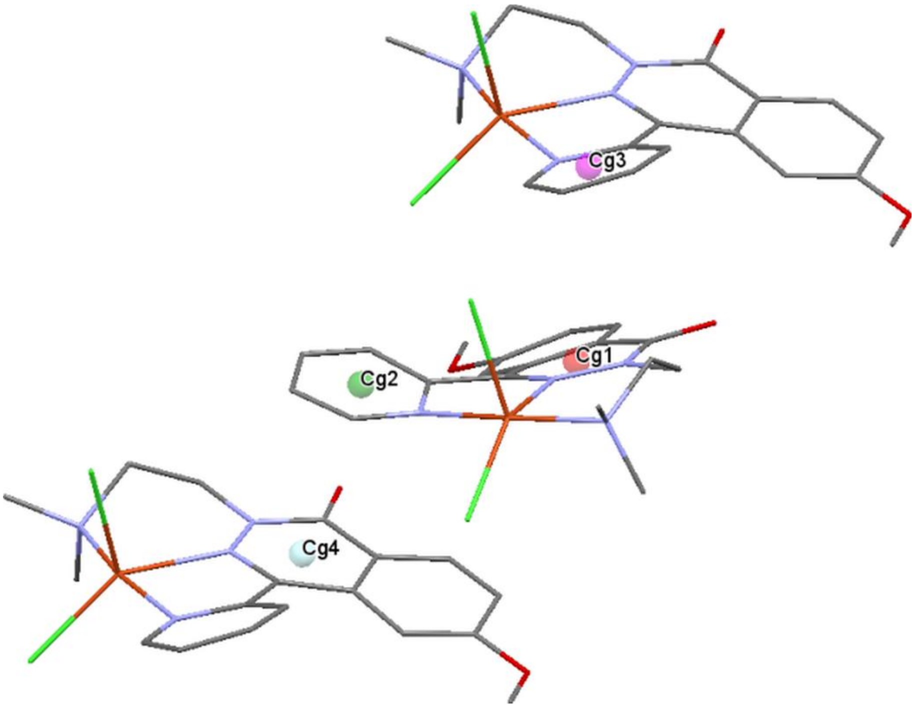

b)

Figure S1: Molecular packing of complex **17**: a) partial molecular packing of complex **17** showing 3D network of hydrogen bonds b) $\pi\cdots\pi$ interaction between rings; Cg1-centroid of the N1/N2/C3/C5/C6/C1 ring, Cg2-centroid of the N3/C2/C14/C8/C7/C4 ring, Cg3-centroid of the N5/C20/C26/C27/C35/C25 ring, and Cg4-centroid of the N7/N8/C23/C22/C21/C19 ring.

Table S2: Hydrogen bond geometry in complex **17** (Å, °).

D-H···A	D-H	H···A	D···A	<D-H···A
C2-H2···O3^{i}	0.95	2.59	3.424(8)	147
C10-H10···O4	0.95	2.40	3.290(7)	155
C13-H13A···Cl3^{ii}	0.99	2.82	3.811(7)	174
C15-H15C···Cl3^{iii}	0.98	2.81	3.782(8)	172
C26-H26···Cl1	0.95	2.82	3.739(6)	163
C36-H36B···Cl1	0.98	2.73	3.622(7)	152

symmetry code: ^{i} *x*, -*y*, 1/2+*z*; ^{ii} 1+*x*,-1+*y*, *z*; ^{iii} 1+*x*,1-*y*,-1/2+*z*.

4. FTIR and vis-NIR Spectroscopy

4.1 FTIR Spectroscopy

The experimental FTIR spectrum of complex **17** was recorded in KBr to confirm a participation of nitrogen atoms of the pyridin-2-yl substituent, 1,2-diazine moiety, and 2-(dimethylamino)ethyl group in a coordination of the copper(II) center (Figure S2). For this purpose, and as the most informative feature, the frequencies of the stretching vibrations of the C-N bond of the pyridin-2-yl substituent, the C=N and N-N bonds of the 1,2-diazine moiety, and the N-Me bond of the 2-(dimethylamino)ethyl group both in the free ligand **7** and its complex with copper(II) were compared (Table S3). The experimental IR absorption bands were assigned based on the theoretical vibrational modes calculated at the CAM-B3LYP/6–311++G(d,p)/LanL2DZ(Cu) level of theory for molecule A of complex **17** in vacuum (Figure S3). In particular, the ν2Py(C-N), ν(C=N), ν(N-N), and ν(N-Me) frequencies for complex **17** were calculated at 1569, 1550, 1211, and 1230 cm^{-1}, respectively, and then they were assigned to the experimental IR absorption bands at 1578, 1531, 1241, and 1241 cm^{-1}, respectively. In the case of the free compound **7**, the stretching frequencies of the 2Py(C-N), C=N, N-N, and N-Me bonds were previously allocated at 1586, 1536, 1274, and 1279 cm^{-1}, respectively [12]. Thus, the observed downshift of the stretching frequencies of the mentioned bonds in complex **17**, compared with those in the free phthalazinone **7**, clearly corroborates the coordination of the copper(II) central ion through nitrogen atoms of the pyridin-2-yl substituent, 1,2-diazine moiety, and 2-(dimethylamino)ethyl group. The analogous lowering of the stretching frequencies of the C-N bond in pyridin-2-yl group and the azomethine C=N bonds in ligands as a result of coordination to copper(II) central ion were observed early [13-15]. Moreover, in the experimental IR spectrum of complex **17** no Cu-N stretching vibrational modes were detected since they occur in the far-

infrared part of the spectrum below 300 cm^{-1} [16]. However, the calculated frequencies of the Cu-N=C, Cu-N_{2Py}, and Cu-NMe stretching vibrations were found at 157, 172, and 270 cm^{-1}, respectively.

Table S3: Comparison of selected experimental and calculated stretching frequencies of ligand **7** and its copper(II) complex **17**.

	Phthalazinone **7**[a]	Complex **17**
	ν_{exp}, ν_{calc} (cm^{-1})	ν_{exp},[b] ν_{calc}[c] (cm^{-1})
2Py(C-N)	1586, 1578	1578, 1569
C=N	1536, 1517	1531, 1550
N-N	1274, 1216	1241, 1211
N-Me	1279, 1257	1241, 1230

[a]Data from [12]. [b]In KBr. [c]Calculated at the CAM-B3LYP/6–311++G(d,p)/LanL2DZ(Cu) level of theory in vacuum and scaled with a factor of 0.9688 [4].

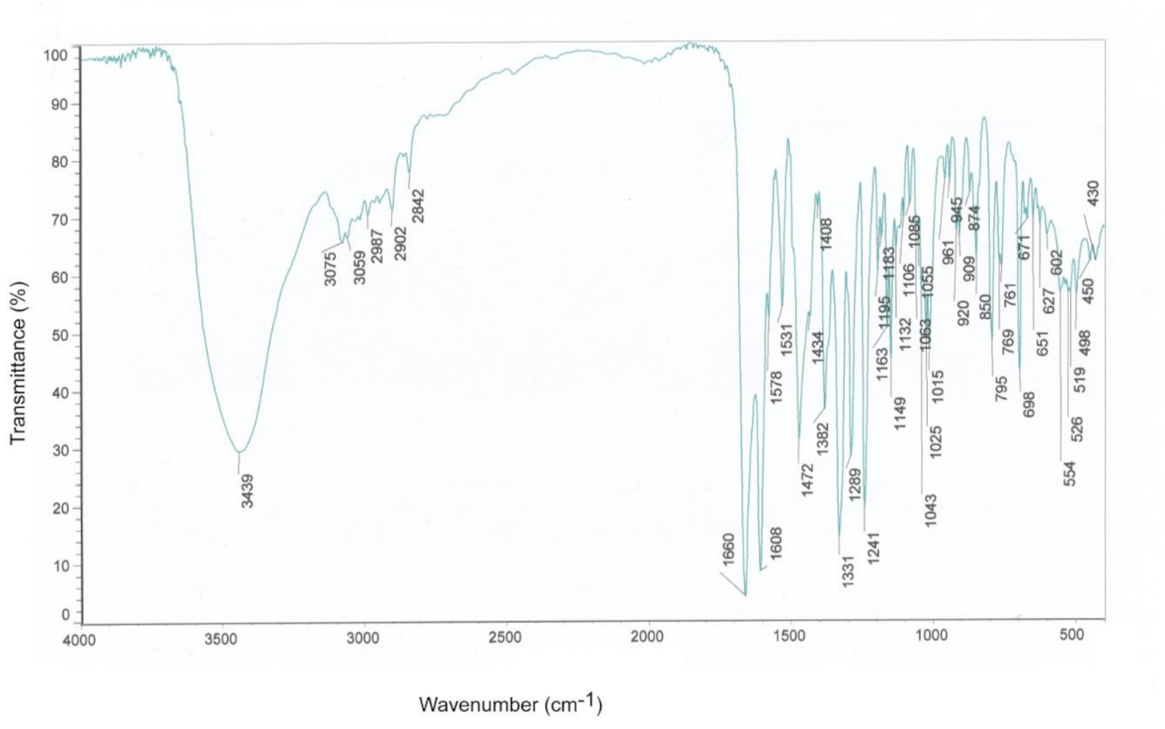

Figure S2: The experimental FTIR spectrum of complex **17** (KBr).

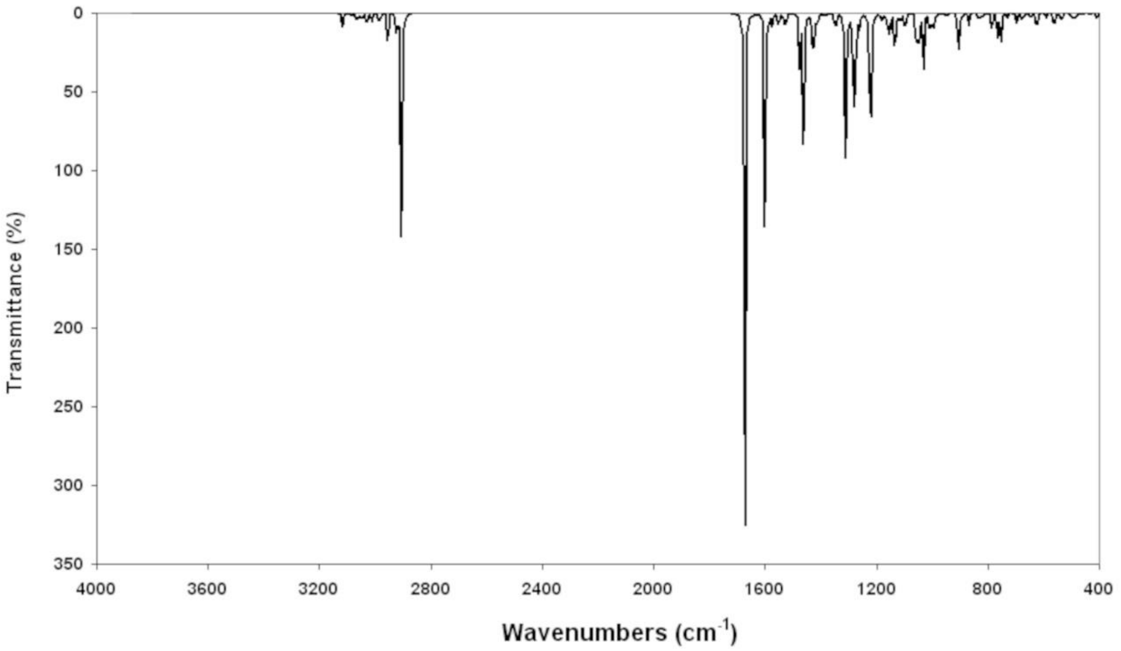

Figure S3: Theoretical vibrational modes calculated at the CAM-B3LYP/6–311++G(d,p)/LanL2DZ(Cu) level of theory for molecule A of complex **17** in vacuum.

4.2 Vis-NIR Spectroscopy

Compound **7** in methanol is not spectroscopically active in the visible region of the electronic spectrum. However, it strongly absorbs in the UV region at wavelengths of 232, 253, and 301 nm [12]. On the other hand, in the vis-NIR spectrum of complex **17** in the same solvent the new absorption bands can be found at 710, 505, 429, and 416 nm (Figure S4, black line). For a detailed analysis of electronic excitations in complex **17**, TD-DFT simulation was performed at the CAM-B3LYP/6–311++G(d,p)/LanL2DZ(Cu) level of theory for molecule A of complex **17** in implicit methanolic environment approximated with the IEF-PCM solvation method (Figure S4, red line). Additionally, the atomic orbital contributions to the molecular orbitals involved in electronic transitions were also calculated.

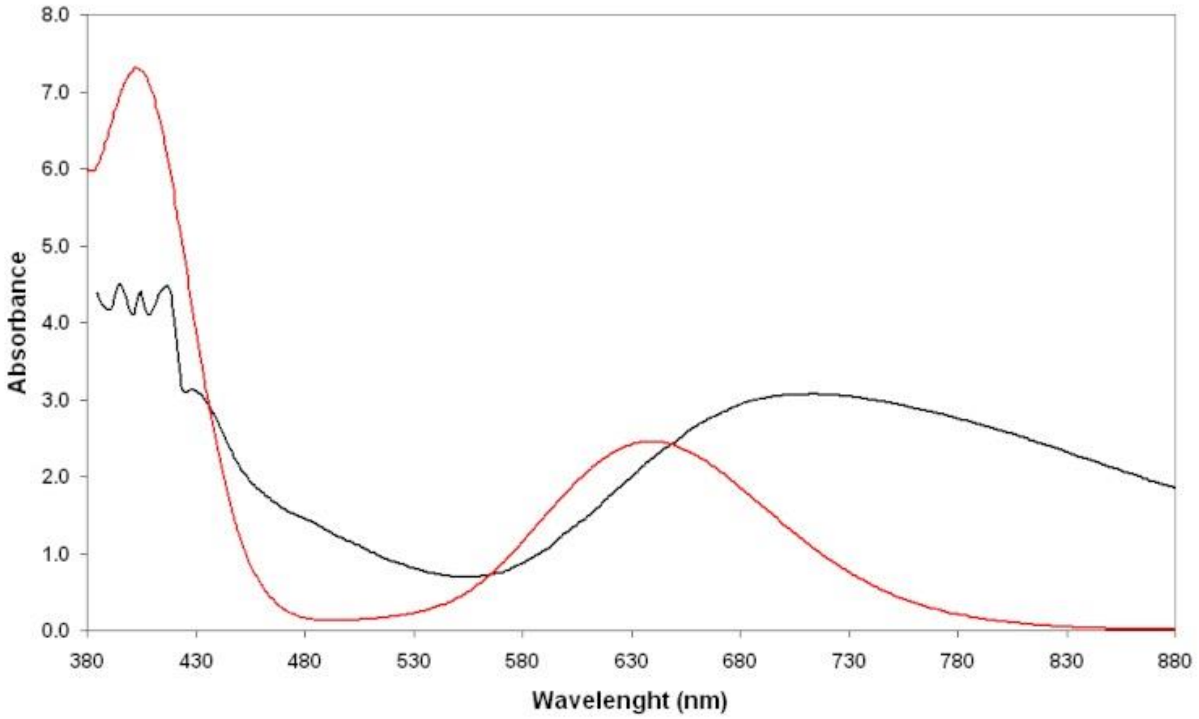

Figure S4: Experimental vis-NIR spectrum of complex **17** in methanol (0.001 mol/dm^3, optical length of 1.0 cm, black line) and theoretical vis-NIR spectrum of complex **17** (red line) calculated at the CAM-B3LYP/6–311++G(d,p)/LanL2DZ(Cu) level of theory in methanol using IEF-PCM solvation model.

The results from TD-DFT calculations given in Table S4 clearly show that the longwave experimental electronic transition at 710 nm can be assigned to the calculated electronic transitions at 649 and 638 nm which mainly display the *d–d* character with some contribution of the MLCT transition type. A similar spectroscopic behavior has been observed in the case of the Cu(II) complex with *N*-methyl-*N*-((6-pivaloylamido-2-pyridyl)-methyl)-*N*-(2-pyridylethyl)amine in methanol with the same coordinate center CuN_3Cl_2 and very similar τ_5 of 0.46 whose longwave *d–d* absorption band could be found at 655 nm [17]. The shortwave absorption bands at 429 and 416 nm were assigned to the theoretical absorption bands at 414, 407, 403, and 392 nm, and taking into account the position of the atomic orbitals which contribute to the ground and

excited states molecular orbitals, these electronic excitations can be denoted as LMCT and ILCT transitions, except that at 392 nm which can be described as a pure LMCT transition. Finally, there is the experimental absorption band at 505 nm which was assigned to the calculated electronic transition at 517 nm, and can be characterized as a pure ILCT state. Moreover, to determine the stoichiometry of complex **17** in methanol solution, the vis-NIR spectra of a series of solutions with an increasing molar ratio of **7** to Cu(II) from 0.0 to 5.0 were recorded (Figure S5). It was found that the absorbance at 710 nm of these solutions reached a clear plateau at a L3:Cu(II) = 1:1 molar ratio, and thus, one can conclude that complex **17** in methanol exhibits the 1:1 metal to ligand stoichiometry.

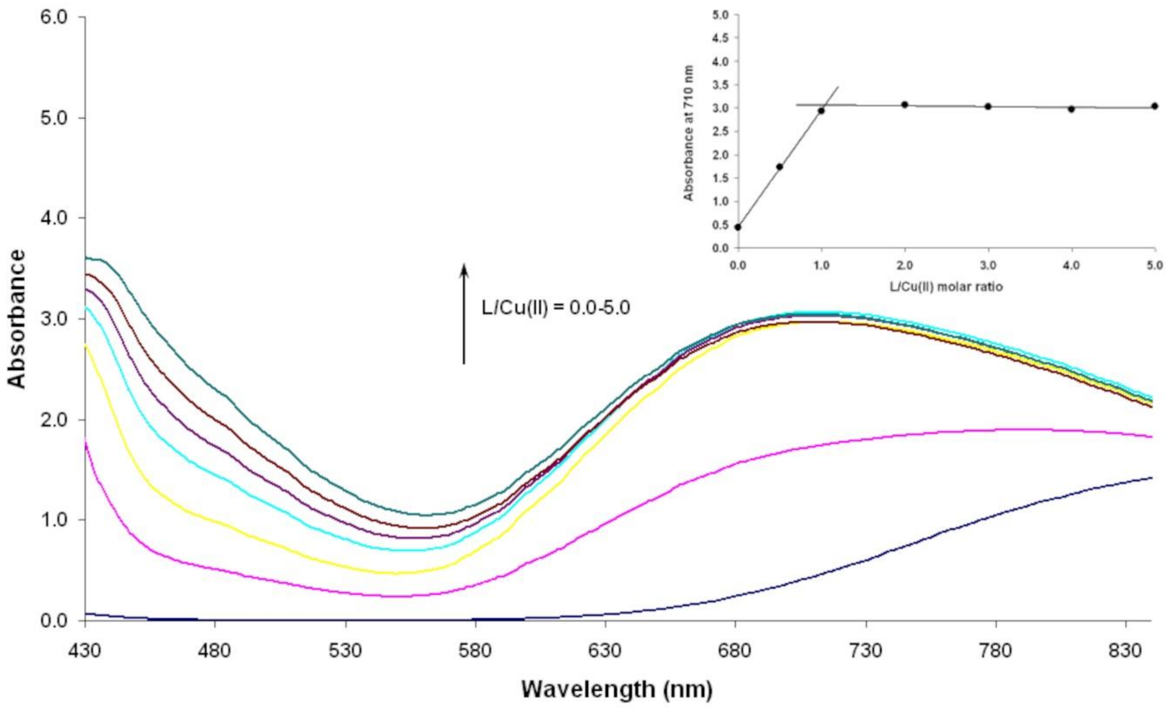

Figure S5: Vis-NIR spectra of a series of solutions with an increasing molar ratio of **7** to Cu(II) from 0.0 to 5.0.

Table S4: Experimental and theoretical spectroscopic parameters of the electronic transitions in complex **17**.

λ_{exp}[a]	λ_{calc}[b]	f[b,c]	Main contribution[d]	Assignment
710	649	0.0002	HOMOβ–23 (d(Cu1) = 44%) → LUMOβ (d(Cu1) = 58%; p(Cl2) = 19%) [31%]	$d \to d$, MLCT
	638	0.0036	HOMOβ–28 (d(Cu1) = 31%; p(C25) = 13%) → LUMOβ ((d(Cu1) = 58%; p(Cl2) = 19%) [15%]	$d \to d$, MLCT
505	517	0.0002	HOMOα (p(N7) = 20%; p(C19) = 16%) → LUMOα (p(N6) = 20%; p(C10) = 17%) [33%]	ILCT
429	414	0.0021	HOMOβ–6 (p(Cl2) = 73%; p(Cl3) = 12%) → LUMOβ (d(Cu1) = 58%; p(Cl2) = 19%) [42%]	LMCT, ILCT
416	407	0.0023	HOMOβ–4 (p(Cl3) = 70%; p(Cl2) = 10%) → LUMOβ (d(Cu1) = 58%; p(Cl2) = 19%) [11%]	LMCT, ILCT
	403	0.0055	HOMOβ–5 (p(Cl2) = 82%) → LUMOβ (d(Cu1) = 58%; p(Cl2) = 19%) [24%]	LMCT, ILCT
	392	0.0013	HOMOβ–5 (p(Cl2) = 82%) → LUMOβ (d(Cu1) = 58%; p(Cl2) = 19%) [37%]	LMCT

[a]In methanol; λ_{exp} in nm. [b]Calculated at the CAM-B3LYP/6–311++G(d,p)/LanL2DZ(Cu) level of theory in methanol using IEF-PCM solvation model; λ_{calc} in nm. [c]f – oscillator strength (dimensionless). [d]The main contribution to electronic transition is given in square brackets; the atomic orbital contribution to the given molecular orbital is provided in round brackets.

5. Cyclic voltammetry

The electrochemical behavior of complex **17** on a platinum disk working electrode in methanol at an ionic strength of 0.1 mol/dm^3 (Et_4NClO_4) was investigated using cyclic voltammetry. Compound **7** itself did not display electrochemical activity on a platinum disk electrode in the potential range from −500 to 750 mV vs. SCE as no oxidation/reduction peaks were found in the voltammogram. However, in the voltammogram of complex **17** (Figure S6, red line) one can find three oxidation peaks at 230, 430, and 545 mV vs. SCE and three reduction peaks at 440, 270, and 115 mV vs. SCE. For comparison purposes, the voltammogram of $CuCl_2 \cdot 2H_2O$ in methanol was also recorded (Figure S6, black line), in which the redox systems Cu(II)/Cu(I) and Cu(I)/Cu(0) in the presence of Cl^- ions can be attributed to the coupled cathodic/anodic peaks at 470/550 mV and 315/390 mV vs. SCE, respectively. Thus, the cathodic peak at 115 mV vs. SCE and the anodic peak at 230 mV vs. SCE in the voltammogram of complex **17** can be assigned to the [(L3)Cu(II)Cl_2] / [(L3)Cu(I)Cl_2] redox couple. This redox system is characterized with a large peak-to-peak separation ($\Delta E_p = E_{pa} - E_{pc}$ = 115 mV) and the peak current ratio much larger then unity (i_{pa}/i_{pc} = 7.54). The observed very low cathodic peak current suggests that [(L3)Cu(II)Cl_2] is involved in the proceeding homogenous chemical reaction which is faster, in the time scale of cyclic voltammetry, than the heterogeneous electron transfer during electroreduction of [(L3)Cu(II)Cl_2] [18].

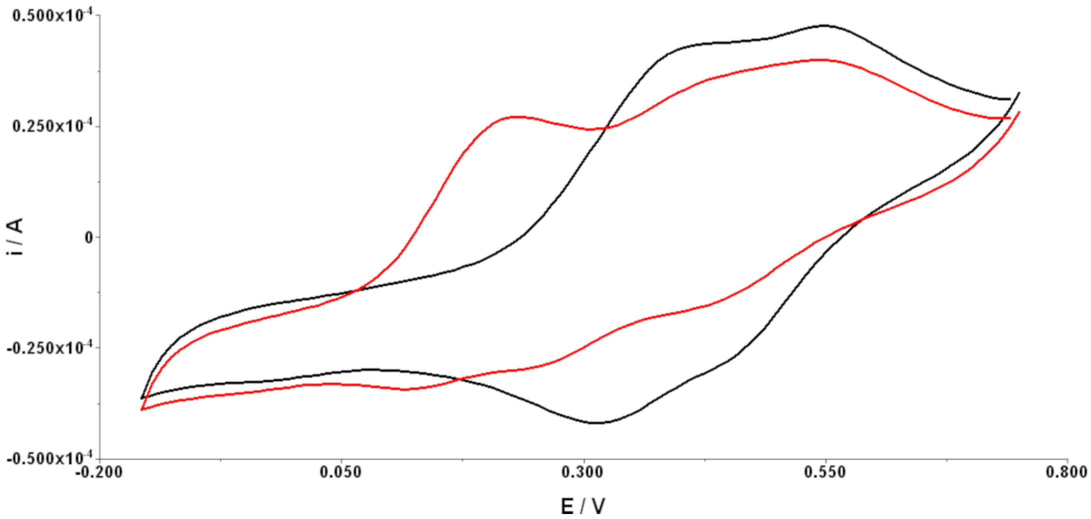

Figure S6: Cyclic voltammograms of complex **17** (0.001 mol/dm^3, red line) and $CuCl_2 \cdot 2H_2O$ (0.001 mol/dm^3, black line) on a platinum disk working electrode in methanol (0.1 mol/dm^3 Et_4NClO_4) at a scan rate of 50 mV s^{-1}. Potentials are given vs. SCE.

6. References

1. CrysAlis PRO, Agilent Technologies, Yarnton, Oxfordshire, England, **2013**.

2. Sheldrick, G. M., *Acta Cryst.* **2015**, *71*, 3-8.
doi: https://doi.org/10.1107/S2053229614024218.

3. Hanwell, M. D., Curtis, D. E., Lonie, D. C., Vandermeersch, T., Zurek, E., Hutchison, G. R. *J. Cheminform*. **2012**, *4*, 17, doi: 10.1186/1758-2946-4-17.

4. Merrick, J. P., Moran, D., Radom, L. *J. Phys. Chem. A* **2007**, *111*, 11683–11700.
doi: https://doi.org/10.1021/jp073974n.

5. Yanai, T., Tew, D., Handy, N. *Chem. Phys. Lett.* **2004**, *393* , 51-57.
doi: https://doi.org/10.1016/j.cplett.2004.06.011.

6. Hay, P. J., Wadt, W. R. *J. Chem. Phys.* **1985**, *82,* 299-310.

doi: https://doi.org/10.1063/1.448975.

7. Cancès, M. T., Mennucci, B., Tomasi, J. *J. Chem. Phys*. **1997**, *107*, 3032–3041. doi: https://doi.org/10.1063/1.474659.

8. Gaussian 09, Revision D.01, Frisch, M. J., Trucks, G. W., Schlegel, H. B., Scuseria, G. E., Robb, M. A., Cheeseman, J. R., Scalmani, G., Barone, V., Mennucci, B., Petersson, G. A., Nakatsuji, H., Caricato, M., Li, X., Hratchian, H. P., Izmaylov, A. F., Bloino, J., Zheng, G., Sonnenberg, J. L., Hada, M., Ehara, M., Toyota, K., Fukuda, R., Hasegawa, J., Ishida, M., Nakajima, T., Honda, Y., Kitao, O., Nakai, H., Vreven, T., Montgomery, Jr., J. A., Peralta, J. E., Ogliaro, F., Bearpark, M., Heyd, J. J., Brothers, E., Kudin, K. N., Staroverov, V. N., Keith, T., Kobayashi, R., Normand, J., Raghavachari, K., Rendell, A., Burant, J. C., Iyengar, S. S., Tomasi, J., Cossi, M., Rega, N., Millam, J. M., Klene, M., Knox, J. E., Cross, J. B., Bakken, V., Adamo, C., Jaramillo, J., Gomperts, R., Stratmann, R. E., Yazyev, O., Austin, A. J., Cammi, R., Pomelli, C., Ochterski, J. W., Martin, R. L., Morokuma, K., Zakrzewski, V. G., Voth, G. A., Salvador, P., Dannenberg, J. J., Dapprich, S., Daniels, A. D., Farkas, O., Foresman, J. B., Ortiz, J. V., Cioslowski, J., Fox, D. J. Gaussian, Inc., Wallingford CT, **2013**.

9. Pakulska, W., Malinowski, Z., Szcześniak, A. K., Czarnecka, E., Epsztajn, J. *Arch. Pharm. Chem. Life Sci*. **2009**, *342*, 41–47.

doi: https://doi.org/10.1002/ardp.200800016.

10 Addison, A. W., Rao, T. N., Reedijk, J., van Rijn, J., Verschoor, G. C. *J. Chem. Soc., Dalton Trans.*, **1984**, 1349-1356. doi: https://doi.org/10.1039/DT9840001349.

11. Steiner, T. *Angew. Chem. Int. Ed*. **2002**, *41*, 48–76.

doi: https://doi.org/10.1002/1521-3773(20020104)41:1%3C48::AID-ANIE48%3E3.0.CO;2-U.

12 Sroczyński, D., Malinowski, Z. *J. Mol. Struct.* **2017**, *1150,* 614–628. doi: https://doi.org/10.1016/j.molstruc.2017.09.020.

13. Mosalkova, A. P., Voitekhovich, S. V., Lyakhov, A. S., Ivashkevich, L. S., Lach, J., Kersting, B., Gaponik, P. N., Ivashkevich, O. A. *Dalton Trans.* **2013**, *42*, 2985–2997. doi: https://doi.org/10.1039/C2DT32512H.

14. Mohan, B., Jana, A., Das, N., Bharti, S., Choudhary, M. *J. Mol. Struct.* **2018**, *1171*, 94-109. doi: https://doi.org/10.1016/j.molstruc.2018.06.016.

15. Milenković, M., Shcherbakov, I. N., D.Popov, L., Levchenkov, S. I., Borodkin, S. A., Alexandrov, G. G. *Polyhedron* **2017**, *121*, 278-284. doi: https://doi.org/10.1016/j.poly.2016.10.020.

16. Saito, Y., Takemoto, J., Hutchinson, B., Nakamoto, K. *Inorg. Chem.* **1972**, *11*, 2003-2011. doi: https://doi.org/10.1021/ic50115a004.

17. Sharma, A. K., Mukherj, R. *Inorganica Chim. Acta* **2008**, *361*, 2768-2776. doi: https://doi.org/10.1016/j.ica.2008.01.047.

18. Zanello, P. *Inorganic Electrochemistry: Theory, Practice and Application*, The Royal Society of Chemistry, Cambridge, **2003**, p. 70.
